# Strategien, Richtlinien und Empfehlungen zur Bewältigung der COVID-19-Pandemie in Einrichtungen der Palliativ- und Hospizversorgung. Ergebnisse eines Scoping-Reviews

**DOI:** 10.1007/s00391-022-02016-8

**Published:** 2022-01-21

**Authors:** Diana Wahidie, Kübra Altinok, Yüce Yılmaz-Aslan, Patrick Brzoska

**Affiliations:** 1 Fakultät für Gesundheit/Department für Humanmedizin, Lehrstuhl für Versorgungsforschung, Universität Witten/Herdecke, Alfred-Herrhausen-Str. 50, 58448 Witten, Deutschland; 2 Fakultät für Gesundheitswissenschaften, AG3 Epidemiologie & International Public Health, Universität Bielefeld, Bielefeld, Deutschland; 3 Fakultät für Gesundheitswissenschaften, AG6 Versorgungsforschung und Pflegewissenschaft, Universität Bielefeld, Bielefeld, Deutschland

**Keywords:** SARS-CoV-2, Maßnahmen, Social Distancing, Infektionskontrolle, Public-Health-Krise, SARS-CoV-2, Measures, Social distancing, Infection control, Public health crisis

## Abstract

**Hintergrund:**

Palliativ- und Hospizeinrichtungen sind durch die COVID-19-Pandemie besonderen Herausforderungen ausgesetzt. Gründe dafür sind insbesondere das hohe Alter und bestehende Vorerkrankungen der Patienten sowie ein körperlich naher Kontakt zwischen Mitarbeitern und Patienten.

**Ziel der Arbeit:**

Ziel der vorliegenden Studie ist es, bestehende Strategien, Richtlinien und Empfehlungen zum Umgang mit den Herausforderungen der COVID-19-Pandemie in der Palliativ- und Hospizversorgung zu ermitteln, die in Deutschland und anderen Ländern zum Einsatz kommen.

**Material und Methoden:**

Im Rahmen eines Scoping Reviews wurden die Datenbanken *PubMed, CINAHL, Web of Science* und *PsycInfo* durchsucht. Zusätzlich wurde „graue Literatur“ über *Google Search* und *Google Scholar* recherchiert. Eingeschlossen wurden alle deutsch- und englischsprachigen Artikel im Zeitraum Januar 2020 bis August 2021, die sich auf die stationäre Palliativ- und Hospizversorgung beziehen.

**Ergebnisse:**

Insgesamt wurden 51 Veröffentlichungen in die Analyse aufgenommen. Die identifizierten Maßnahmen lassen sich 10 verschiedenen Kategorien zuordnen: Maßnahmen zur Infektionskontrolle, strukturelle Maßnahmen, Besuchsregelungen, Kommunikationsstrukturen, Schulung und Aufklärung, psychosoziale Unterstützungsmaßnahmen, spezifische Überlegungen für Menschen mit Demenz, vorausschauende Pflegeplanung, Abschiednahme sowie Maßnahmen nach dem Tod.

**Diskussion:**

Bei der Bewältigung der COVID-19-Pandemie dominieren insbesondere Maßnahmen zur Ermöglichung der Kommunikation zwischen Patienten, Mitarbeitern und Angehörigen, psychosoziale Unterstützungsmaßnahmen, strukturelle Maßnahmen und Empfehlungen zur Schulung und Aufklärung aller Beteiligten. Strategien zur Palliativversorgung von Menschen mit Demenz in Pandemiezeiten sowie zu Abschiednahme und Trauerbewältigung von Hinterbliebenen finden weniger Beachtung.

**Zusatzmaterial online:**

Zusätzliche Informationen sind in der Online-Version dieses Artikels (10.1007/s00391-022-02016-8) enthalten.

## Abstract (en)

**Background:**

Palliative and hospice care facilities face different challenges emerging from the COVID-19 pandemic. In particular, this results from the high age and pre-existing diseases of patients as well as the physically close contact between staff and patients.

**Objective:**

The aim of this study was to identify existing strategies, guidelines and recommendations in Germany and other countries with respect to addressing the challenges of the COVID-19 pandemic in palliative and hospice care.

**Material and methods:**

The databases *PubMed, CINAHL, Web of Science* and *PsycInfo* were searched as part of a scoping review. Additionally, *Google Search* and *Google Scholar* were used to identify “grey literature”. All German and English language articles related to inpatient palliative and hospice care were included which were published between January 2020 and August 2021.

**Results:**

A total of 51 publications were included in the analysis. The identified measures can be clustered into 10 different categories: infection control measures, structural measures, visiting regulations, communication structures, education and training, psychosocial support measures, specific considerations for people with dementia, advance care planning, farewell and measures after death.

**Conclusion:**

With respect to addressing the COVID-19 pandemic, the primary focus is on measures to enable communication between patients, staff and relatives, psychosocial support measures, structural measures and recommendations for training and education of all stakeholders. Measures for the palliative care of people with dementia in times of a pandemic as well as the farewell and grief management of bereaved families received less attention.

## Einleitung

Durch die rasche Ausbreitung des SARS-CoV-2-Virus (Severe acute respiratory syndrome coronavirus type 2) waren und sind v. a. Einrichtungen der Palliativ- und Hospizversorgung herausgefordert [1]. Patienten solcher Versorgungseinrichtungen sind aufgrund ihres häufig höheren Alters und bestehender Vorerkrankungen einem erhöhten Risiko für einen schweren Krankheitsverlauf von COVID-19 (Coronavirus Disease 2019) ausgesetzt [2]. Infolge des engen physischen Kontakts zwischen Mitarbeitern und Patienten bei der Durchführung pflegerischer Leistungen und der Vielzahl an Gemeinschaftsaktivitäten besteht zudem eine hohe Infektionsgefahr [3]. Die zur Senkung des Ansteckungsrisikos eingeführten Infektionsschutzmaßnahmen, wie z. B. Besuchs- und Ausgangsbeschränkungen, erhöhen aufgrund des Wegfalls von Unterstützungspersonen (z. B. Ehrenamtliche) die Arbeitsbelastungen des Personals und tragen gleichzeitig zu einer erschwerten Abschiednahme von sterbenden Patienten bei [4].

Um den aufgezeigten Herausforderungen zu begegnen, müssen Palliativ- und Hospizeinrichtungen innovative Ansätze entwickeln, die den Bedürfnissen aller Akteure Rechnung tragen. Ziel des vorliegenden Beitrags ist es, bestehende Strategien, Richtlinien und Empfehlungen zum Umgang mit der COVID-19-Pandemie in der stationären Palliativ- und Hospizversorgung auf Basis verfügbarer internationaler Literatur zu ermitteln. Die Erkenntnisse des Beitrags können sowohl Leitungskräfte stationärer Einrichtungen als auch politische Akteure dabei unterstützen, ihre bisherigen Strategien und Maßnahmen im Umgang mit der COVID-19-Pandemie zu bewerten und ggf. im Hinblick auf zukünftige Public-Health-Krisen anzupassen.

## Methodik

Zur Erfassung bestehender Strategien, Richtlinien und Empfehlungen wurde ein Scoping-Review aller in deutscher und englischer Sprache verfügbaren Publikationen aus dem Zeitraum 01.01.2020 bis 17.08.2021 in den Datenbanken *PubMed, CINAHL, PsycInfo* und *Web of Science* sowie den Internetsuchmaschinen *Google Search* und *Google Scholar* durchgeführt. Anders als bei einem systematischen Review liegt der Fokus dabei nicht darauf, die Wirksamkeit bestehender Maßnahmen zu beurteilen – da bisher in der Regel kaum analytische Studien zum Thema vorliegen, wäre dies kaum möglich – sondern darauf, zusammenfassend einen Überblick über einen neu entstandenen Forschungsbereich zu ermöglichen. Die Strukturierung erfolgte auf Basis eines fünfstufigen Vorgehens (Zusatzmaterial online) [5–7] und wird entlang der Empfehlungen der „Preferred Reporting Items for Systematic Reviews and Meta-Analyses – Extension for Scoping Reviews“ (PRISMA-ScR) berichtet [8].

## Ergebnisse

**Figure 1:**
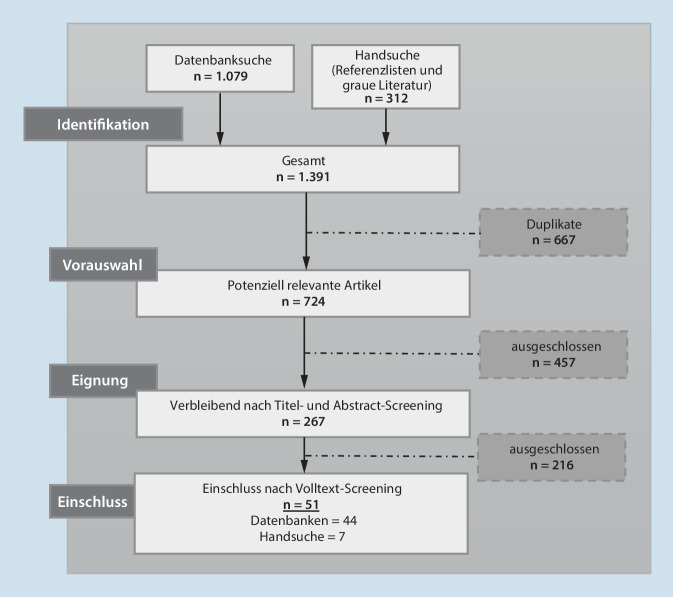

Aus insgesamt 1391 Treffern wurden 51 Artikel final in die Analyse eingeschlossen (Abb. 1). Die identifizierten Strategien, Richtlinien und Empfehlungen lassen sich 10 verschiedenen Ebenen zuordnen, die im Folgenden erläutert werden *(für nähere Informationen: Zusatzmaterial online, Tab. 3 und 4)*.

### Maßnahmen zur Infektionskontrolle

Ein Großteil der Veröffentlichungen (*n* = 20) berichtet von der Einführung grundlegender Maßnahmen zur Infektionskontrolle. Dazu gehören die regelmäßige Reinigung der Stationen, Sprechzimmer und aller Oberflächen [9, 10], die Einführung von zusätzlichen Handwaschvorrichtungen [11, 12] und die strikte Einhaltung von Kontaktbeschränkungen [13, 14]. Darüber hinaus empfehlen die einbezogenen Artikel die regelmäßige Fensterlüftung [15], die Reduktion von Teambesprechungen, die Aussetzung weiterer „face-to-face meetings“, wie beispielsweise Supervisionen und Fortbildungen [16], und die Vermeidung der Versammlung von Personengruppen in engen Räumen [17]. Zudem wird empfohlen, dass nicht das gleiche Personal Patienten mit und ohne COVID-19 behandelt [18]. Weitere Maßnahmen zur Infektionskontrolle stellen das tägliche Gesundheitsscreening von Mitarbeitern und Patienten [19], die regelmäßige Testung der Mitarbeiter, Patienten, Angehörigen und anderer Besucher auf SARS-CoV‑2 [10, 18, 20–22] sowie die Ausstattung mit persönlicher Schutzausrüstung dar [10, 18, 19, 22].

### Strukturelle Maßnahmen

Insgesamt 29 Publikationen berichten von organisationalen und personellen Umstrukturierungsmaßnahmen als Reaktion auf die COVID-19-Pandemie. Zu ersteren gehört die Einteilung der Einrichtung in verschiedene Zonen, die Patienten mit Verdacht auf oder bestätigter COVID-19-Infektion von Patienten trennt, die negativ auf COVID-19 getestet wurden [15, 23–25]. Zu den personellen Umstrukturierungsmaßnahmen gehören die Bildung von festen Mitarbeiterkohorten und die Zuordnung dieser zu festen Patienten, um ggf. auftretende Infektionen auf eine kleinere Gruppe von Kontaktpersonen zu beschränken [15, 19, 26].

Um Personalengpässen entgegenzuwirken, kann auf Zeitarbeitspersonal zurückgegriffen, das Personal innerhalb der Einrichtung umverteilt oder Urlaubssperren verhängt werden [13]. Andere Artikel berichten darüber hinaus von der Rekrutierung von Personal aus der arbeitslosen Bevölkerung [18] oder aus einem Pool von Medizinstudierenden [27]. Strategien zur Bewältigung knapper materieller Ressourcen stellen die kontrollierte Ausgabe und Rationierung von Schutzausrüstung sowie die eigene Herstellung von Schutzmaterialien und Desinfektionsmitteln dar [13]. Zudem können Träger, Verbände, Ämter und Behörden sowie Angehörige bei der Beschaffung von Schutzmaterialien zur Hilfe gezogen werden [13].

### Besuchsregelungen

Zahlreiche Einrichtungen (*n* = 6) berichten von einer Änderung der Besuchsregelungen im Rahmen der COVID-19-Pandemie, die sich sowohl in der Beschränkung der Zahl von Besuchern [22, 28–30] wie auch der Verkürzung von Besuchszeiten widerspiegelt [29, 31]. Einige Publikationen (*n* = 3) empfehlen die Einführung von Ausnahmeregelungen für Patienten am Lebensende [4, 21, 26]. Es kann beispielsweise ein gemeinsamer oder gestaffelter Besuch mehrerer Angehöriger in einem Einzelzimmer mit guter Lüftungsmöglichkeit ermöglicht werden. Falls ein einrichtungsinterner Besuch aufgrund hoher Infektionszahlen nicht möglich ist, sollten Möglichkeiten im Freien geschaffen werden, wie beispielsweise Balkon‑/Fensterbesuche oder Besuche über die Terrasse bzw. den Garten [26]. Besucher sollten während der Besuche einlagige Kleidung tragen, Schmuck ablegen und Wertsachen begrenzen [32].

### Kommunikationsstrukturen

Um einen physischen Kontakt zu vermeiden, aber dennoch soziale Interaktionen zu ermöglichen, wurde vielfach der Einsatz digitaler Kommunikationstools thematisiert (*n* = 28) [4, 9, 12, 13, 17, 19, 21, 22, 24, 26–28, 31, 33–48]. Videokommunikationstools wie Zoom oder Skype können sowohl für die Kommunikation zwischen Personal und Patienten als auch für den Kontakt zwischen Patienten und ihren Angehörigen nützlich sein [4, 21, 43]. Kliniken sollten für die Nutzung solcher Videokommunikationstools Smartphones, Tablets und Laptops sowie einen kostenlosen Internetzugang bereitstellen [21, 33, 34, 37–39]. Eine weitere Möglichkeit des virtuellen Dienstes stellt die virtuelle Realität (VR) dar, die jedoch mit hohen Kosten verbunden ist [43].

Um Hindernissen bei der Kommunikation mittels Mund-Nasen-Bedeckungen entgegenzuwirken, empfehlen einige Artikel (*n* = 3) Mitarbeitern und Besuchern deutlicher, langsamer und/oder lauter zu sprechen, verstärkte gestische Kommunikation anzuwenden [19] oder auch durchsichtige Gesichtsmasken zu verwenden [33, 49].

### Schulung und Aufklärung

Um die Eigenverantwortlichkeit und das Gefahrenbewusstsein der Mitarbeiter zu fördern, führten einige Einrichtungen Schulungen zur praktischen Umsetzung der Hygienemaßnahmen, zum korrekten An- und Ablegen der persönlichen Schutzausrüstung und zum Umgang mit digitalen Kommunikationsmitteln durch [12, 23, 37, 50]. Neben der Schulung des Personals sollten auch Patienten und ihre Angehörigen über die aktuelle Lage der COVID-19-Pandemie und die geltenden Richtlinien aufgeklärt werden [26]. Für die Schulung können beispielsweise E‑Learning-Plattformen [13], Hands-on-Trainings [19], Aushänge in der Einrichtung [26] oder auf die jeweilige Zielgruppe zugeschnittene Aufklärungsmaterialien, z. B. in Form von Flyern oder Videoclips [26, 50], eingesetzt werden.

### Psychosoziale Unterstützungsmaßnahmen

Um einer psychischen und physischen Überlastung des Personals entgegenzuwirken, wird vorgeschlagen, diesen Rückzugsräume in der Einrichtung, genügend Pausen und eine gute Verpflegung bereitzustellen [4]. Die Leitungspersonen der Gesundheitseinrichtungen sollten eine vertrauensvolle Atmosphäre schaffen [19], Kommunikationsbereitschaft signalisieren und Mitarbeiter in Entscheidungen einbeziehen [13, 51]. Um die Motivation der Mitarbeiter zu erhöhen, stellten einige Einrichtungen zusätzliche Getränke, Gutscheine, Verpflegungsgeld, kostenlose Parkplätze, Supervisionen, Kinderbetreuungshilfen und virtuelles Yoga zur Verfügung [13, 24].

Um einer sozialen Isolation und Vereinsamung der Patienten entgegenzuwirken, können diesen aufgezeichnete Hörbücher, Karten/Briefe, gemalte Bilder oder Fotos der Angehörigen zur Verfügung gestellt werden [4, 35, 52, 53]. Weiterhin können die Versorgungsanbieter Konzerte, Gottesdienste oder Geburtstage im Außenbereich der Einrichtung veranstalten, an denen die Patienten vom Fenster oder vom Balkon aus teilnehmen können [19, 26]. Sowohl Patienten und ihren Angehörigen als auch Mitarbeitern sollte ein Zugang zu einrichtungsinternen und -externen Fachkräften, die eine psychosoziale Betreuung anbieten, ermöglicht werden [4, 19, 34, 54].

### Spezifische Überlegungen für Menschen mit Demenz

Demenzerkrankte Patienten sollten gesondert bei der ordnungsgemäßen Anwendung der Hygienemaßnahmen unterstützt werden. Dazu können beispielsweise demenzfreundliche Hinweistafeln, die an die Händehygiene erinnern, angebracht werden. Um die Einhaltung physischer Distanzierungsmaßnahmen bei Menschen mit Demenz sicherzustellen, können Indoor-Hobbys nützlich sein. Zur Vorbeugung von Ängsten im Zusammenhang mit der COVID-19-Pandemie sollten negative Ausdrücke wie „Krise“, „Pandemie“ oder „Isolierung“ in Gegenwart der Demenzerkrankten vermieden werden. Auch bei der Nutzung digitaler Kommunikationstools benötigen Menschen mit Demenz zusätzliche Unterstützung durch die Mitarbeiter [25].

### Vorausschauende Pflegeplanung

Versorgungsanbieter sollten rechtzeitig Gespräche zur Vorausplanung der Pflege mit den Patienten und ihren Angehörigen vereinbaren, um über Therapieziele, Behandlungspräferenzen, den bevorzugten Behandlungs‑/Sterbeort [17, 19, 25, 26, 34, 54], gewünschte Rituale und Bestattungs‑/Gedenkpläne zu sprechen [55] und unerwünschte und belastende Behandlungen zu vermeiden [25, 54]. In Leitlinien wird zudem empfohlen, bei vorhandenen Patientenverfügungen aus der Zeit vor der COVID-19-Pandemie die aktuelle Relevanz zu überprüfen [19, 26, 54].

### Abschiednahme

Mehreren Angehörigen sollte, entweder gemeinsam oder zeitlich versetzt, eine Abschiednahme im nahen Umfeld des Patienten unter Einhaltung grundlegender Hygienemaßnahmen gestattet werden [25, 26]. Sofern dies nicht möglich ist, sollten digitale Kommunikationsmittel [25] oder auch Fotos der Verstorbenen zum Abschiednehmen genutzt werden [21, 52, 56]. Falls es die Infektionslage zulässt, sei es zudem empfehlenswert, Handabdrücke oder ein Herzbild der sterbenden Person anzufertigen. Diese können die Hinterbliebenen nach dem Tod mit den Patienten verbinden [36]. Die Deutsche Gesellschaft für Palliativmedizin empfiehlt darüber hinaus zur Erleichterung des Prozesses der Abschiednahme für die Angehörigen den Rückgriff auf Palliativpsychologen als Vermittler zwischen Klinik und Angehörigen [52].

### Maßnahmen nach dem Tod

Einrichtungen sollten Hinterbliebene proaktiv auf Unterstützungsdienste zur Trauerbewältigung hinweisen [26], ihnen aktuelle Informationen für die Organisation einer Beerdigung bereitstellen und Familien in sozioökonomischen Risikosituationen einen Hinweis auf öffentliche Beihilfen geben [34, 57]. Falls die Teilnahme an der Bestattung aufgrund der Pandemie nur wenigen Personen gewährleistet werden kann, sollte auf digitale Möglichkeiten verwiesen werden [26].

## Diskussion

Die eingeschlossenen Publikationen zeigen auf, dass bei der Bewältigung der Pandemie v. a. Kommunikationsstrategien, Empfehlungen zur psychosozialen Unterstützung aller Akteure, strukturelle Strategien und Schulungs- und Aufklärungsmaßnahmen dominieren. Es ist jedoch festzuhalten, dass bei Personalengpässen eine Aufteilung in kleine Mitarbeiterteams und eine Zuordnung dieser auf einzelne Patienten organisatorisch schwer umsetzbar ist. Auch die Bildung von Isolierstationen kann aufgrund von fehlenden räumlichen Kapazitäten erschwert sein [58]. Die in fast allen Publikationen thematisierte Nutzung digitaler Kommunikationstools kann für körperlich und kognitiv eingeschränkte Patienten schwierig sein. Zudem ist die Anschaffung notwendiger IT-Technik mit erhöhten Kosten verbunden, welche die Einrichtungen im Rahmen der derzeitigen Pandemie möglicherweise nicht abdecken können und daher entsprechend finanziell und strukturell unterstützt werden müssen [59].

Nur eine Minderheit der eingeschlossenen Artikel gibt gesonderte Empfehlungen für die Palliativ- und Hospizversorgung von Menschen mit Demenz in Pandemiezeiten aus. Dies stellt ein Beispiel für eine vulnerable Bevölkerungsgruppe dar, der vergleichsweise wenig Aufmerksamkeit gewidmet wurde [25]. Es bedarf demnach bei der Entwicklung von Strategien und Empfehlungen zur Bewältigung von Public-Health-Krisen einer erhöhten Aufmerksamkeit für besonders vulnerable Zielgruppen. Weiterhin gibt es nur eine geringe Anzahl an Publikationen, die sich mit der Abschiednahme und Maßnahmen nach dem Tod, wie z. B. der Trauerbewältigung von Hinterbliebenen oder Bestattungsvorgängen in Pandemiezeiten, beschäftigen [60]. Nur ein geringer Anteil der eingeschlossenen Artikel gibt Empfehlungen zur vorausschauenden Pflegeplanung aus. Gespräche zur Vorausplanung der Pflege sind insbesondere in Zeiten von COVID-19 unerlässlich, da sie unerwünschte lebenserhaltende Behandlungen verhindern und damit auch Ressourcen des Gesundheitssystems, die in Pandemiezeiten umso beschränkter sind, schonen [61].

## Fazit für die Praxis

- Es besteht ein Bedarf an Studien zu: Palliativversorgung von Menschen mit Demenz in Zeiten von COVID-19, Abschiednahme und Maßnahmen nach dem Tod.
- Experten aus dem Bereich der Palliativ- und Hospizversorgung, Patienten und ihre Angehörigen sollten in die Entwicklung von Leitlinien einbezogen werden.
- Identifizierte Strategien und Empfehlungen sollten in der Praxis erprobt und evaluiert werden.

## Supplementary Information




