# Supplementary material for: Strategies, guidelines and recommendations for coping with the COVID-19 pandemic in palliative and hospice care facilities. Results of a scoping review
Source: Z Gerontol Geriatr. 2022 Jan 21;55(2):151–6. [Article in German] doi: 10.1007/s00391-022-02016-8 (PMC8780045; doi:10.1007/s00391-022-02016-8)
Supplement: Supplementary file 1 [file 391_2022_2016_MOESM1_ESM.docx]

Strategien, Richtlinien und Empfehlungen zur Bewältigung der COVID-19-Pandemie in Einrichtungen der Palliativ- und Hospizversorgung. Ergebnisse eines Scoping Reviews

Supplement: Details zur methodischen Vorgehensweise

*Schritt 1: Identifikation der Forschungsfrage*

Gemäß des Population-Concept-Context-Rahmens (PCC) [7] bezieht sich das vorliegende Scoping Review auf Patienten, deren Angehörige und Mitarbeiter stationärer Einrichtungen der Palliativ- und Hospizversorgung. Das zu untersuchende *Konzept* sind die Strategien, Richtlinien und Empfehlungen zum Umgang mit der COVID-19-Pandemie im *Kontext* der stationären Palliativ- und Hospizversorgung. Die Forschungsfrage des vorliegenden Beitrags lautet entsprechend: Welche bestehenden Strategien, Richtlinien und Empfehlungen gibt es im Umgang mit den Herausforderungen der COVID-19-Pandemie in der stationären Palliativ- und Hospizversorgung?

Zur Beantwortung der Forschungsfrage wurden alle in deutscher und englischer Sprache verfügbaren Publikationen aus dem Zeitraum 01.01.2020 bis 17.08.2021 berücksichtigt. Eingeschlossen wurden Artikel, die sich auf die stationäre Palliativ- und Hospizversorgung beziehen. Es wurden aber auch Publikationen aus anderen Versorgungsbereichen, wie dem akutstationären Sektor, einbezogen, wenn sie auf die Palliativ- und Hospizversorgung übertragbar waren. Gemäß der Definition der Definition der Deutschen Gesellschaft für Palliativmedizin (DGP) werden im Rahmen dieser Untersuchungen mit Palliativ- bzw. Hospizeinrichtungen Einrichtungen verstanden, die einen Schwerpunkt auf die „Behandlung von Patienten mit einer nicht heilbaren progredienten und weit fortgeschrittenen Erkrankung mit begrenzter Lebenserwartung [legen], für die das Hauptziel der Begleitung die Lebensqualität ist.“^[[1]](#footnote-1)^ Bei der Art der Publikation sowie dem Forschungsdesign wurden keine Einschränkungen vorgenommen. Dies dient der breiten Abdeckung der vorhandenen Evidenz. Ausgeschlossen wurden Artikel, die keine nachvollziehbare Beschreibung der betreffenden Strategie, Richtlinie oder Empfehlung enthalten, sich nicht auf die Palliativ- und Hospizversorgung übertragen lassen, aus dem ambulanten Bereich stammen oder nur Empfehlungen zur medikamentösen Symptomkontrolle enthalten.

*Schritt 2: Identifikation relevanter Studien*

Um potenziell relevante Artikel zu identifizieren, wurde eine systematische Recherche in den Datenbanken PubMed, CINAHL, PsycInfo und Web of Science durchgeführt. Dazu wurde eine breit angelegte Suchstrategie genutzt, welche Begriffe für konkrete Strategien sowie Begriffe für die Palliativ- und Hospizversorgung und COVID-19 umfasste (*Tabelle 1)*. Darüber hinaus wurden im Rahmen einer Handsuche auch die Literaturlisten der identifizierten Veröffentlichungen berücksichtigt sowie über die Internetsuchmaschinen Google Search und Google Scholar nach „grauer“ Literatur gesucht. Die Suche bei Google Search und Google Scholar erfolgte auf Basis der ersten 100 Treffer *(Tabelle 2).* Die Veröffentlichungen wurden von zwei Reviewerinnen (DW, KA) unabhängig voneinander auf Basis der Titel- und Abstracts und der Volltexte auf ihren Ein- oder Ausschluss überprüft.

*Schritt 3: Auswahl der Studien*

Insgesamt konnten nach der Entfernung von 667 Duplikaten 724 potenziell relevante Artikel identifiziert werden. In einem ersten Schritt wurden die Titel und Abstracts dieser Artikel auf der Grundlage der Ein- und Ausschlusskriterien überprüft. 457 dieser Artikel wurden ausgeschlossen, da sie nicht zur Forschungsfrage passten. Die Volltexte der verbliebenen 267 Artikel wurden wiederum in einem zweiten Schritt entlang der Ein- und Ausschlusskriterien bewertet. Hierbei wurden 216 dieser Veröffentlichungen ausgeschlossen, da sie entweder nur Empfehlungen zur medikamentösen Symptomkontrolle enthielten, sich auf die ambulante Versorgung bezogen oder nicht auf den Bereich der Palliativ- und Hospizversorgung übertragbar waren. Es verblieben insgesamt 51 Veröffentlichungen, die eingeschlossen wurden (*Abbildung 1 im Artikel*).

*Schritt 4: Extraktion der Daten*

Die wichtigsten Grundmerkmale der Publikationen wurden von einer Reviewerin (DW) in eine Tabelle extrahiert *(Tabelle 3).* Zu den extrahierten Merkmalen gehörten Angaben zu Autoren, Datum der Veröffentlichung, Land/Region, Publikationsart, Studiendesign, Zielsetzung der Studie sowie die primär für die Fragestellung relevanten Strategien, Richtlinien und Empfehlungen.

*Schritt 5: Zusammenstellen, Zusammenfassen und Berichten der Ergebnisse*

Die eingeschlossenen Publikationen wurden von einer Reviewerin (DW) im Hinblick auf bestehende Strategien, Richtlinien und Empfehlungen zum Umgang mit pandemiebedingten Herausforderungen untersucht. Auf der Grundlage der 51 einbezogenen Publikationen wurden induktiv zehn Kategorien *(Tabelle 4)* abgeleitet, die von allen Autoren gemeinsam überprüft wurden.

*Charakteristika der Publikationen*

Von den 51 eingeschlossenen Artikeln stammen 14 aus den USA (9, 14, 28, 36, 38, 39, 42, 44, 45, 48-50, 55, 62), elf aus Deutschland [4, 13, 15-17, 19, 21, 26, 46, 52, 56], neun aus dem Vereinigten Königreich [10, 24, 27, 31, 34, 35, 41, 47, 51], drei aus den Niederlanden [25, 37, 54], zwei aus Afrika [11, 12], zwei aus Italien [20, 30] und zwei aus Australien [22, 32]. Andere Länder mit nur einer Studie waren Israel [18], Singapur [33], China [23], Spanien [57], Kanada [43], Frankreich [53], Brasilien [40] und Taiwan [29]. Nur zwölf der eingeschlossenen Publikationen stellen Erhebungen auf der Basis einer empirischen Untersuchung dar [11-13, 22, 24, 28-30, 35, 37, 47, 49]. Bei den restlichen Artikeln handelt es sich um Erfahrungsberichte von Krankenhäusern [21, 23, 27, 42, 50, 53, 57, 62], Hospizen [48] sowie Palliativstationen und -diensten [9, 20, 39, 44], Diskussions- bzw. Positionspapiere [18, 32, 51, 55], Übersichtsarbeiten [10, 25, 31, 34, 36, 38, 40, 41, 43, 46], Letter to the Editor [33], Meinungsartikel [45] und Empfehlungen verschiedener wissenschaftlicher Fachgesellschaften und Institutionen [4, 14-17, 19, 26, 52, 54, 56]*.*

*Reflektion der Methodik*

Zu den Stärken des vorliegenden Beitrages zählt die Nutzung einer breiten Suchstrategie in zwei Sprachen, welche in sechs Datenbanken eingesetzt wurde und auch „graue Literatur“ berücksichtigte. Dadurch ist von einer umfassenden Identifikation relevanter Publikationen auszugehen. Eine wesentliche Limitation der Untersuchung ergibt sich aus der fehlenden Überprüfung der Wirksamkeit und Durchführbarkeit identifizierter Empfehlungen, Richtlinien und Strategien. Dies ist jedoch auch nicht das Ziel eines Scoping Reviews. Eine weitere Einschränkung, die sich aus der Dynamik der COVID-19-Pandemie ergibt, besteht darin, dass die identifizierten Strategien, Richtlinien und Empfehlungen des vorliegenden Beitrags mit zunehmenden Erfahrungen im Umgang mit COVID-19 und der ansteigenden Zahl von geimpften Personen voraussichtlich weiterentwickelt werden oder an Relevanz verlieren. Gleichwohl sind Erkenntnisse des vorliegenden Reviews für den Umgang mit zukünftigen Krisen relevant. Zukünftige Studien sollten die Wirksamkeit und Durchführbarkeit der bisher identifizierten Maßnahmen überprüfen, um Entscheidungsträger in Palliativ- und Hospizeinrichtungen zu einer evidenzbasierten Entscheidung zu verhelfen. Zur Evaluation der Wirksamkeit ausgewählter Maßnahmen können Anbieter der stationären Palliativ- und Hospizversorgung, Patienten und ihre Angehörigen als Experten aus der Praxis einbezogen werden.

| Englischsprachige  Suchbegriffe | (concept* OR strateg* OR measure* OR programme* OR management* OR managing OR instrument* OR tool* OR guideline* OR recommendation* OR preparedness* OR response*) AND (hospice* OR palliative* OR “palliative care” OR “hospice care” OR end-of-life OR “terminal care” OR “end-of-life care”) AND (SARS-CoV-2 OR COVID-19 OR COVID19 OR “corona virus” OR “corona pandemic” OR pandemic* OR “corona infection” OR “severe acute respiratory syndrome coronavirus 2” OR “coronavirus disease 2019” OR 2019-nCoV OR “novel coronavirus” OR “Wuhan coronavirus” OR “Wuhan seafood market pneumonia virus”) |
| --- | --- |
| Deutschsprachige  Suchbegriffe | (Konzept* OR Maßnahme* OR Strategie* OR Instrument* OR Tool* OR Management* OR Plan OR Umgang* OR Richtlinie* OR Empfehlung* OR Leitlinie* OR Vorschlag OR Leitfaden OR Vorbereitung* OR Reaktion*) **AND** (Hospiz* OR Palliativ* OR Hospizversorgung OR Palliativversorgung OR End-of-life OR “Versorgung am Lebensende”) **AND (**SARS-CoV-2 OR COVID-19 OR COVID19 OR Coronavirus OR “Corona Pandemie” OR Corona-Pandemie OR Pandemie OR “Corona Infektion” OR “severe acute respiratory syndrome coronavirus 2” OR “Coronavirus Krankheit 2019” OR 2019-nCoV OR “neuartiges Coronavirus” OR “Wuhan Coronavirus”) |

***Tabelle 1: Im Rahmen des Scoping Reviews verwendete englisch- und deutschsprachige Suchbegriffe***

***Tabelle 2: Dokumentation der Datenbank- und Handsuche***

| **Datenbank** | **Suchbegriffe** | **Datum der Durchführung** | **Anzahl der Treffer** | **Anzahl der Duplikate** | | **Anzahl der Treffer nach Duplikatentfernung** | | **Filter** | |
| --- | --- | --- | --- | --- | --- | --- | --- | --- | --- |
| **PubMed** | **(((concept*[Title/Abstract] OR strateg*[Title/Abstract] OR measure*[Title/Abstract] OR programme*[Title/Abstract] OR management*[Title/Abstract] OR managing[Title/Abstract] OR instrument*[Title/Abstract] OR tool*[Title/Abstract] OR guideline*[Title/Abstract] OR recommendation*[Title/Abstract] OR preparedness*[Title/Abstract] OR response*[Title/Abstract]) AND (hospice*[Title/Abstract] OR palliative*[Title/Abstract] OR "palliative care"[Title/Abstract] OR "hospice care"[Title/Abstract] OR end-of-life[Title/Abstract] OR "terminal care"[Title/Abstract] OR "end-of-life care"[Title/Abstract])) AND (SARS-CoV-2[Title/Abstract] OR COVID-19[Title/Abstract] OR COVID19[Title/Abstract] OR "corona virus"[Title/Abstract] OR "corona pandemic"[Title/Abstract] OR pandemic*[Title/Abstract] OR "corona infection"[Title/Abstract] OR "severe acute respiratory syndrome coronavirus 2"[Title/Abstract] OR "coronavirus disease 2019"[Title/Abstract] OR 2019-nCoV[Title/Abstract] OR "novel coronavirus"[Title/Abstract] OR "Wuhan coronavirus"[Title/Abstract] OR "Wuhan seafood market pneumonia virus"[Title/Abstract])) AND ("2020/01/01"[pdat] : "2021/08/17"[pdat])** | 17.08.2021 | 251 | 0 | | 251 | | - 01.01.2020-17.08.2021 - Free Full Text - Full Text - Humans - English - German | |
| **CINAHL** | **AB ( concept* OR strateg* OR measure* OR programme* OR management* OR managing OR instrument* OR tool* OR guideline* OR recommendation* OR preparedness* OR response* ) AND AB ( hospice* OR palliative* OR “palliative care” OR “hospice care” OR end-of-life OR “terminal care” OR “end-of-life care” ) AND AB ( SARS-CoV-2 OR COVID-19 OR COVID19 OR “corona virus” OR “corona pandemic” OR pandemic* OR “corona infection” OR “severe acute respiratory syndrome coronavirus 2” OR “coronavirus disease 2019” OR 2019-nCoV OR “novel coronavirus” OR “Wuhan coronavirus” OR “Wuhan seafood market pneumonia virus” )** | 17.08.2021 | 73 | 39 | | 285 | | - **Limiters** - Published Date: 20200101-20210831; Human - **Expanders** - Apply equivalent subjects - **Narrow by Language:**- English; German - **Search modes** - Boolean/Phrase | |
|  | AB ( Konzept* OR Maßnahme* OR Strategie* OR Instrument* OR Tool* OR Management* OR Plan OR Umgang* OR Richtlinie* OR Empfehlung* OR Leitlinie* OR Vorschlag OR Leitfaden OR Vorbereitung* OR Reaktion* ) AND AB ( Hospiz* OR Palliativ* OR Hospizversorgung OR Palliativversorgung OR End-of-life OR “Versorgung am Lebensende” ) AND AB ( SARS-CoV-2 OR COVID-19 OR COVID19 OR Coronavirus OR “Corona Pandemie” OR Corona-Pandemie OR Pandemie OR “Corona Infektion” OR “severe acute respiratory syndrome coronavirus 2” OR “Coronavirus Krankheit 2019” OR 2019-nCoV OR “neuartiges Coronavirus” OR “Wuhan Coronavirus” ) | 17.08.2021 | 49 | 43 | | 291 | | - **Limiters** - Published Date: 20200101-20210831; Human - **Expanders** - Apply equivalent subjects - **Narrow by Language:**- English; German - **Search modes** - Boolean/Phrase | |
| **PsycInfo** | AB ( concept* OR strateg* OR measure* OR programme* OR management* OR managing OR instrument* OR tool* OR guideline* OR recommendation* OR preparedness* OR response* ) AND AB ( hospice* OR palliative* OR “palliative care” OR “hospice care” OR end-of-life OR “terminal care” OR “end-of-life care” ) AND AB ( SARS-CoV-2 OR COVID-19 OR COVID19 OR “corona virus” OR “corona pandemic” OR pandemic* OR “corona infection” OR “severe acute respiratory syndrome coronavirus 2” OR “coronavirus disease 2019” OR 2019-nCoV OR “novel coronavirus” OR “Wuhan coronavirus” OR “Wuhan seafood market pneumonia virus” ) | 17.08.2021 | 22 | 9 | | 304 | | - **Limiters** - Published Date: 20200101-20210831; Population Group: Human - **Expanders** - Apply equivalent subjects - **Search modes** - Boolean/Phrase | |
|  | AB ( Konzept* OR Maßnahme* OR Strategie* OR Instrument* OR Tool* OR Management* OR Plan OR Umgang* OR Richtlinie* OR Empfehlung* OR Leitlinie* OR Vorschlag OR Leitfaden OR Vorbereitung* OR Reaktion* ) AND AB ( Hospiz* OR Palliativ* OR Hospizversorgung OR Palliativversorgung OR End-of-life OR “Versorgung am Lebensende” ) AND AB ( SARS-CoV-2 OR COVID-19 OR COVID19 OR Coronavirus OR “Corona Pandemie” OR Corona-Pandemie OR Pandemie OR “Corona Infektion” OR “severe acute respiratory syndrome coronavirus 2” OR “Coronavirus Krankheit 2019” OR 2019-nCoV OR “neuartiges Coronavirus” OR “Wuhan Coronavirus” ) | 17.08.2021 | 11 | 11 | | 304 | | - **Limiters** - Published Date: 20200101-20210831; Population Group: Human - **Expanders** - Apply equivalent subjects - **Search modes** - Boolean/Phrase | |
| **Web of Science** | **concept* OR strateg* OR measure* OR programme* OR management* OR managing OR instrument* OR tool* OR guideline* OR recommendation* OR preparedness* OR response***(Abstract) and**hospice* OR palliative* OR “palliative care” OR “hospice care” OR end-of-life OR “terminal care” OR “end-of-life care”**(Abstract) and**SARS-CoV-2 OR COVID-19 OR COVID19 OR “corona virus” OR “corona pandemic” OR pandemic* OR “corona infection” OR “severe acute respiratory syndrome coronavirus 2” OR “coronavirus disease 2019” OR 2019-nCoV OR “novel coronavirus” OR “Wuhan coronavirus” OR “Wuhan seafood market pneumonia virus”**(Abstract) and **2020-01-01/2021-08-17**(Publication Date) and**English**or**German(**Languages) | 17.08.2021 | 408 | 232 | | 480 | | - Publication Date: 01.01.2020-17.08.2021 - Languages: English or German | |
|  | **Konzept* OR Maßnahme* OR Strategie* OR Instrument* OR Tool* OR Management* OR Plan OR Umgang* OR Richtlinie* OR Empfehlung* OR Leitlinie* OR Vorschlag OR Leitfaden OR Vorbereitung* OR Reaktion***(Abstract) and**Hospiz* OR Palliativ* OR Hospizversorgung OR Palliativversorgung OR End-of-life OR “Versorgung am Lebensende”**(Abstract) and**SARS-CoV-2 OR COVID-19 OR COVID19 OR Coronavirus OR “Corona Pandemie” OR Corona-Pandemie OR Pandemie OR “Corona Infektion” OR “severe acute respiratory syndrome coronavirus 2” OR “Coronavirus Krankheit 2019” OR 2019-nCoV OR “neuartiges Coronavirus” OR “Wuhan Coronavirus”** (Abstract) and**2020-01-01/2021-08-17**(Publication Date) and**English**or**German**(Languages) | 17.08.2021 | 265 | 231 | | 514 | | - Publication Date: 01.01.2020-17.08.2021 - Languages: English or German | |
| **Google Scholar** | (recommendation* OR guideline* OR strateg* OR measure* OR response* OR management* OR managing) AND (palliative* OR hospice* OR end-of-life) AND (COVID-19 OR SARS-CoV-2) | 17.08.2021 | 100 | 58 | 42 | | - 2020-2021 - English, German - Sortiert nach Relevanz - Bis einschließlich Seite 10 durchsucht | |  |
|  | (Empfehlung* OR Leitlinie* OR Richtlinie* OR Leitfaden OR Strategie* OR Maßnahme* OR Reaktion* OR Management*) AND (Palliativ* OR Hospiz* OR End-of-life) AND (COVID-19 OR SARS-CoV-2) | 17.08.2021 | 100 | 12 | 130 | | - 2020-2021 - English, German - Sortiert nach Relevanz - Bis einschließlich Seite 10 durchsucht | |  |
| **Google Search** | (recommendation* OR guideline* OR strateg* OR measure* OR response* OR management* OR managing) AND (palliative* OR hospice* OR end-of-life) AND (COVID-19 OR SARS-CoV-2) | 17.08.2021 | 100 | 27 | 203 | | - 1.1.2020-17.8.2021 - Bis einschließlich Seite 10 durchsucht | |  |
|  | (Empfehlung* OR Leitlinie* OR Richtlinie* OR Leitfaden OR Strategie* OR Maßnahme* OR Reaktion* OR Management*) AND (Palliativ* OR Hospiz* OR End-of-life) AND (COVID-19 OR SARS-CoV-2) | 17.08.2021 | 9 | 5 | 207 | | - 1.1.2020-17.8.2021 | |  |
| **Referenzlisten-Suche** |  |  | 3 Treffer | 0 | 3 Treffer | |  | |  |

***Tabelle 3: Übersicht der eingeschlossenen Publikationen***

| **Autor*innen und Datum der Veröffentlichung** | **Land/**  **Region** | **Publikationsart** | **Studiendesign** | **Ziel** | **Strategien/Maßnahmen/Empfehlungen/Richtlinien**  (rot: Kategorie; s. Tabelle 4) |
| --- | --- | --- | --- | --- | --- |
| Abdihamid O et al.; September 2020 | China | Bericht einer onkologischen Abteilung eines Krankenhauses |  | Schaffung eines Überblicks über Prävalenz und Auswirkungen von COVID-19 auf Krebspatient*innenen sowie über institutionelle Empfehlungen zur Eindämmung des Corona-Virus.  Vorstellung des einrichtungsinternen Organisationsmodells zum Schutz von Krebspatient*innen vor COVID-19 | - Schulung des gesamten medizinischen Personals hinsichtlich aktueller Informationen über COVID-19 und darüber, wie sie in den jeweiligen Abteilungen nach COVID-19-Fällen suchen können **(5.)** - Einberufung eines COVID-19-Ad-hoc-Sachverständigenausschusses bestehend aus Expert*innen für Infektionskrankheiten, Hämatolog*innen und Onkolog*innen, Pharmazeut*innen und Radiolog*innen **(2.)** - Zutritt von Personal zu anderen Abteilungen außer ihren eigenen ohne Erlaubnis nicht möglich, um Kreuzinfektionen zu minimieren **(2.)** - Einteilung der Einrichtung in vier Zonen, um Patient*innen vor der Aufnahme in die Einrichtung zu überprüfen: **(2.)** - Zone 1 (Screening- und Überwachungszone): Für Patient*innen vorgesehen, die nach Anweisung des Expertenausschusses überwacht werden müssen, um eine mögliche Infektion auszuschließen **(2.)** - Zone 2 (Quarantänezone für Verdachtsfälle): Für Verdachtsfälle vorgesehen, so dass jede*r Patient*in in einem Einzelzimmer unter Quarantäne gestellt wird **(2.)** - Zone 3 (Quarantänezone für bestätigte Fälle): Behandlungszone für Patient*innen mit bestätigter COVID-19-Infektion **(2.)** - Zone 4 (onkologische Station): Behandlungszone für Krebspatient*innen, die nicht an COVID-19 erkrankt sind **(2.)** |
| Adams C; März 2020 | USA | Bericht eines Hospizes |  | Beschreibung der Erfahrungen eines Hospizes im Rahmen der COVID-19-Pandemie und Ausgabe von Empfehlungen zum Umgang mit der Pandemiesituation | - Selbstfürsorge praktizieren und vorleben: jeden Morgen bei der Teambesprechung Beschreibung einer positiven Veränderung durch die Pandemie **(6.)** - Planung, eine Person mit Sammeln und Verteilen wichtiger E-Mail-Benachrichtigungen zu beauftragen, um die ständige Berührung mit nationalen Nachrichten zu vermeiden **(4.)** - Festlegung eines einzelnen Teammitglieds, das täglich Kontakt mit Familienmitgliedern aufnimmt **(4.)** - Planung, Videokommunikationstools anzuschaffen, da Kommunikation per Telefon nur begrenzt möglich ist **(4.)** - Palliativteams sollten einen Notfallplan für die Personalbesetzung entwickeln **(2.)** - Empfehlung der Verlegung einiger Teammitglieder ins Home-Office, um Belastung des Krankenhauses zu verringern und Arbeitskräfte zu schonen **(2.)** |
| Apoeso O et al.; April 2021 | USA | Bericht einer Palliativstation |  | Beschreibung der Umwandlung einer Palliativstation in eine COVID-19-Station für Patient*innen am Lebensende | - Räumliche Umstrukturierung: **(2.)** - Sichtfenster an den Türen zur Verbesserung der Sicht auf den/die Patient*in **(2.)** - Einsatz und Positionierung von Zimmerkameras zur Überwachung des Patientenkomforts **(2.)** - Einführung der Einzelbettbelegung **(2.)** - Verwendung von iPads für FaceTime/Zoom-Familientreffen und die Kommunikation mit dem*r Patient*in **(4.)** - Ausbildung des Personals: **(5.)** - Schulung des Personals sollte die Beurteilung von Symptomen am Lebensende, Isolationsmaßnahmen und Aufklärung über Verabreichung von Medikamenten umfassen **(5.)** - Die Einrichtung hat dem Personal COVID-19-Updates über die Anzahl der Fälle, Informationen über sich ändernde Richtlinien und Praktiken, Protokolle für die Medikamentenverabreichung und den Einsatz persönlicher Schutzausrüstung (PSA) zur Verfügung gestellt **(5.)** - Einstellung eines zusätzlichen geriatrischen Oberarztes, eines Assistenzarztes und eines Krankenpflegers **(2.)** - Pflegestationen und alle Oberflächen der Station wurden während der Geschäftszeiten alle zwei Stunden gereinigt, nachts mit reduzierter Häufigkeit **(1.)** - Sozialarbeiter*innen und Seelsorger*innen richteten wöchentliche „Kaffegespräche“ für Mitarbeiter*innen ein, um emotionaler und physischer Belastung durch die Pandemie entgegenzuwirken und ein sicheres und unterstützendes Forum für die Mitarbeiter*innen bereitzustellen **(6.)** - Für die persönlichen Gespräche wurde ein ruhiger Raum eingerichtet **(2.)** - Leitung der Palliativstation rief Mitarbeiter*innen wöchentlich an und bot ihnen Unterstützung und Wellness-Ressourcen an **(6.)** - Es ist empfehlenswert, einen Aufenthaltsraum für das Personal mit sanfter Musik, angemessener Beleuchtung und Büchern bereitzustellen **(6.)** |
| Bains J et al.; August 2020 | USA | Bericht einer Notaufnahme |  | Beschreibung des Einsatzes von Telemedizinwagen in COVID-19-Isolierzimmern der Notaufnahme, um eine häufigere Kommunikation zwischen Personal und Patient*innen zu ermöglichen sowie PSA zu schonen | - Bereitstellung von 19 Avizia C250 Telemedizinwagen **(4.)** - Ausstattung der Wagen mit Tablets und hochauflösenden Schwenk-Neige-Zoom-Kameras von Sony, die eine Videokommunikation ermöglichen **(4.)** - Jedes Isolierzimmer wurde mit einem Festnetztelefon als Backup für die Wagen ausgestattet **(4.)** - Log-in in ein gemeinsames Avizia-Benutzerkonto, Start des Videoanrufs und Verwendung eines Headsets **(4.)** - Eingehende Anrufe werden automatisch auf dem Bildschirm des*r Patient*in angezeigt und beantwortet, so dass Patient*innen das System nicht berühren müssen **(4.)** - Nach der Verbindungsherstellung konnten die Mitarbeiter*innen den Zoom und die Positionierung der Kamera steuern, indem sie die gewünschte Stelle auf dem Bildschirm anklickten **(4.)** - Kurze 5-minutüge Schulung des Personals zur Navigation des Telemedizinwagens durch Personal mit Erfahrung in der Nutzung der Plattform **(5.)** - Peer-to-Peer-Schulung: Jede*r geschulte Mitarbeiter*in wurde gebeten, zwei andere Personen in der Nutzung des Systems zu unterweisen **(5.)** |
| Bausewein C und Simon S; Juni 2021 | Deutschland | Empfehlungen des Forschungsverbunds Palliativmedizin im Netzwerk Universitätsmedizin (NUM) |  | Entwicklung einer Nationalen Strategie für die Betreuung von schwerkranken und sterbenden Menschen und ihren Angehörigen in Pandemiezeiten (PallPan) | Patient*innen und Angehörige unterstützen:   - Versorger*innen sollen Menschen, die schwerkrank sind oder zur Risikogruppe für schweren Infektionsverlauf gehören und ihren Angehörigen Gespräche über Therapieziele und Behandlungspräferenzen (u.a. zu Krankenhauseinweisung, Behandlung auf einer Intensivstation und Reanimation) frühzeitig anbieten **(8.)** - Versorger*innen sollen den aktuell erklärten, vorausverfügten oder mutmaßlichen Willen der infizierten und nicht-infizierten schwerkranken oder sterbenden Menschen bezüglich einer indizierten Therapie erfassen und berücksichtigen **(8.)** - Prüfen, ob bereits verfasste Patientenverfügungen und weitere Willensbekundungen aktualisiert sind **(8.)** - Leiter*innen der Einrichtungen/Dienste dürfen den sterbenden Menschen nicht den Besuch und die Begleitung durch ihre Angehörigen verweigern **(3.)** - Der Zugang ist frühzeitig zu gewährleisten (bei Verdacht auf eine nahende Sterbephase und möglichst bevor eine Kommunikation mit dem schwerkranken Menschen nicht mehr möglich ist) **(3.)** - Besuch von mehreren nahen Angehörigen gemeinsam oder gestaffelt ermöglichen **(3.)** - Gemeinsame Abschiednahme in Einzelzimmern mit guter Lüftungsmöglichkeit unter Einhaltung der Hygienemaßnahmen **(1.);** **(9.)** - Ausnahmeregelungen für Besuche für alle Teammitglieder transparent kommunizieren und im Dokumentationssystem eintragen **(3.)** - Das Feiern von Festen, z.B. Geburtstagen, ermöglichen (mit begrenzter Personenzahl oder draußen) zur Steigerung der Lebensqualität **(6.)** - Einrichtung von Infektionszimmern und -stationen **(2.)** - Einlass durch den Neben-/Terrasseneingang **(2.)** - Möglichkeiten im Freien schaffen, z.B. Balkon-/Fensterbesuche, Spaziergänge, Terrasse, Garten **(3.)** - Leiter*innen der Einrichtungen sollen für Besuche der Angehörigen ausreichend Schutzausrüstung sowie geschultes Personal zur sachgerechten Anleitung der Besucher*innen und Sicherstellung der Einhaltung der Regelungen bereitstellen **(3.); (1.)** - Leiter*innen sollen für den Fall, dass persönliche Gespräche nicht oder nur eingeschränkt möglich sind, den schwerkranken und sterbenden Menschen und ihren Angehörigen Alternativen der Begleitung anbieten, z.B. durch Mitarbeiter*innen, Seelsorger*innen oder Ehrenamtliche/Hospizdienst **(6.)** - Versorger*innen und Leiter*innen der Einrichtungen sollen für den Fall, dass persönliche Gespräche durch Angehörige nicht oder nur eingeschränkt möglich sind, andere Kommunikationsmittel bereitstellen **(4.)** - Bei der Nutzung ggf. unterstützen und hierfür Mitarbeiter*innen schulen **(5.)** - Art der Medien nach Wünschen der Patient*innen und ihrer Angehörigen richten (Telefon, digitale Medien) **(4.)** - Video-Telefonate ermöglichen **(4.)** - Tablets und Smartphones bereitstellen **(4.)** - Entwicklung und Implementierung einer Telefon-Hotline für Fragen zur palliativmedizinischen Versorgung, Gesprächen mit Angehörigen und Coaching für Mitarbeiter*innen **(4.)** - Bei Kontaktbeschränkungen sollen Versorger*innen regelmäßig Kontakt mit Angehörigen aufnehmen, um sie über die Situation der Patient*innen zu informieren und sie in Entscheidungen einzubeziehen: **(4.)** - Feste Ansprechpartner*innen, klare Zuständigkeiten und feste Zeiten verabreden **(4.)** - Empathische Kommunikation u.a. durch Wertschätzung und Verständnis sowie Vermittlung von Sicherheit **(4.)** - Digitale Kommunikationswege proaktiv nutzen und technische Voraussetzungen hierfür erbringen **(4.)** - Ein medizinisch informierter zusätzliche*r Mitarbeiter*in auf der Station (in Gesprächsführung mit Angehörigen geschult) kann den Kontakt zu Angehörigen aufbauen **(4.); (5.)** - Niedrigschwellige Angebote an das Personal mit Hinweisen zur empathischen Kommunikation in schwierigen Situationen z.B. Postkarten oder „Bierdeckel“ mit typischen Sätzen in kritischen Kommunikationssituationen **(4.)** - Leiter*innen sollen Patient*innen und Angehörige über lokale Pandemielage und geltenden Regelungen regelmäßig informieren **(5.)** - Kommunikationsverantwortliche in Pandemieplänen festlegen und benennen **(4.)** - Tagesaktuell Informationen niederschwellig (einfacher Zugang und leichte Sprache) zur Verfügung stellen (5.) - Mögliche Kommunikationswege: Homepage, Newsletter, Aushänge, Hotline, Pressemitteilung **(5.); (4.)** - Informationsmaterial für Patient*innen und Angehörige: Flyer, Beitrag in gesundheitsspezifischen Medien, Videoclips etc. **(5.)** - Bildung von Task Forces, die pandemiebezogene Anweisungen tagesaktuell veröffentlichen **(2.)** - Das Abschiednehmen von Verstorbenen (infiziert/nicht-infiziert) soll am Sterbeort oder im nahen Umfeld des Sterbens ermöglicht werden **(9.)** - Versorgende und Leiter*innen ermöglichen gemeinsames oder gestaffeltes Abschiednehmen und informieren Angehörige frühzeitig über bestehende Regelungen **(9.)** - Bestatter*innen ermöglichen den Abschied **(9.)** - Abschiedsraum auf Infektionsstation, auf dem Leichnam aufgebahrt und hergerichtet wird (letzte Fotos für Angehörige machen, Verabschiedung durch Team) **(9.)** - Broschüre mit Anregungen für alternative Abschiedsrituale **(9.)** - Religionsgemeinschaften und kommunale Verwaltungen ermöglichen Teilnahme an Bestattungen unter Einhaltung der Hygienevorschriften sowie individuelles Totengedenken auf Friedhöfen: **(10.)** - Als Alternative oder Ergänzung digitale Übertragungsmöglichkeiten von Bestattungen bereithalten **(10.)** - Trauerfeier/Totengedenken wird z.B. ins Freie verlegt oder die Trauernden in Gruppen aufgeteilt, um allen den Abschied zu ermöglichen **(10.)** - Kultursensibler Umgang mit unterschiedlichen Abschiedsritualen **(10.)** - Versorgende und Einrichtungen sollen Hinterbliebene über Angebote zur Unterstützung in der Trauer proaktiv hinweisen und bedarfsorientierte Angebote machen: **(10.)** - Trauerbank: Zeiten, zu denen Trauernde mit Ehrenamtlichen auf einer Bank Gespräche führen können **(10.)** - Angehörige erhalten besondere Trauer-/Beileidskarte mit Ansprechpersonen/Kontaktdaten **(10.)** - Trauerhotline **(10.)**   Mitarbeitende unterstützen:   - Leiter*innen sollen bestmöglichen Infektionsschutz für ihre Mitarbeitenden auf Basis des Erreger-spezifischen Standards (RKI-definiert) gewährleisten: (1.) - Mitarbeiter*innen wiederholt zum Umgang mit infizierten Personen und zum Gebrauch der persönlichen Schutzausrüstung schulen, um Sicherheit zu vermitteln und Ängste zu reduzieren **(5.)** - Ausreichend Schutzausrüstung und Testmöglichkeiten zur Verfügung stellen **(1.)** - Bei Ressourcenmangel: Priorisierung bei der Verteilung von Schutzausrüstung **(2.)** - Personalplanung in getrennten Clustern (feste Mitarbeiterkohorten) **(2.)** - Um potentielle Infektionsausbrüche überschaubar zu halten, werden Mitarbeiter*innen in festen Zimmern/Patient*innen zugeordnet **(2.)** - Leiter*innen sollen regelmäßig Mitarbeiter*innen über die Pandemiesituation vor Ort und die aktuellen Regelungen informieren **(5.)** - Regelmäßige Mitarbeitertreffen/Besprechungen, Rundmail, Homepage, Aushänge, Hotline, Briefings der Infektionsstation zur Lage in der Einrichtung **(5.)** - Alltagstaugliche, zielgruppenspezifische, wissenschaftlich fundierte Informationsplattformen **(5.)** - Personal in Krisenkommunikation schulen und einsetzen, um Informationen zu aktuellen Regelungen aufzubereiten und an Fachpersonal im Gesundheitswesen weiterzuleiten **(5.)** - Betriebsärzt*in für Fragerunde der Mitarbeiter*innen ins Haus holen **(5.)** - Leiter*innen sollen regelmäßig Mitarbeiter*innen ermuntern, pandemiebezogene Probleme, Hindernisse, Bedarfe und eigene Bedürfnisse zu berichten, um Abhilfe bzw. Lösungen zu finden **(6.)** - Pinnwand zum Sammeln von Themen anstelle von Teamsitzungen, um Ansammlung zu vieler Mitarbeiter*innen in einem Raum zu vermeiden **(6.)** - Einrichtung einer „Pandemie-E-Mail-Adresse“ **(6.)** - Leiter*innen sollen für Mitarbeiter*innen in einer Pandemiesituation Unterstützungsangebote schaffen: **(6.)** - Niederschwellige Angebote, z.B. Hotlines bereitstellen **(6.)** - Psychosoziale/seelsorgerische Unterstützung anbieten **(6.)** - Informations-/Unterstützungsmaterial bereithalten **(6.)** - Gespräche anbieten **(6.)** - Supervision unter Einhaltung der Hygienevorschriften (z.B. mehrmals in Kleingruppen, draußen, digital, hybrid) **(6.)** - Proaktiv informieren, persönliches Gespräch suchen, Präsenz zeigen, offene Kommunikation **(6.)** - Jede*r Mitarbeiter*in kann einen (bemalten) Stein zur Erinnerung an eine*n Patient*in an einem Ort im Garten der Einrichtung ablegen **(6.)** - Wertschätzende Gesten und Leistungen, wie Gutscheine, Prämien, Karten **(6.)** - Tägliche Unterstützungsmaßnahmen in Hochphasen der Pandemie (gratis Wasser, Obst und Mittagessen, gratis Parkangebote) **(6.)** |
| Beneria A et al.; Mai 2021 | Spanien | Bericht eines Universitätskrankenhauses |  | Beschreibung der Struktur, des Ablaufs und der Funktionen eines End-of-Life-Interventionsprogramms, das während der COVID-19-Pandemie in einem Universitätskrankenhaus eingeführt wurde | - Sechs Gesundheitsozialarbeiter*innen und 18 klinische Psycholog*innen waren am Programm beteiligt **(6.)** - Hauptaufgaben der Gesundheitssozialarbeiter*innen bestanden darin, soziale Schwachstellen und Bedürfnisse zu ermitteln, um diese zu beheben (z.B. Informationen über Bestattungsvorgänge, Weiterleitung an soziale Einrichtungen) **(6.); (10.)** - Die klinischen Psycholog*innen sollten den emotionalen Zustand und die Bedürfnisse der Angehörigen einschätzen und entsprechend intervenieren (z.B. Erleichterung des emotionalen Ausdrucks, Überweisung an spezialisierte Trauer- oder Familienhilfeprogramme) **(6.),** - Die vom EOL-Team durchgeführten Interventionen wurden in zwei Arten von Fällen unterteilt: (1) EOL-Situationen und (2) familiäre Interventionen zur Mitteilung eines Verlustes an eine*n Patient*in - (1) EOL-Situationen: - Phase 1: Aktivierung und Koordinierung - Telematische Koordinierung zwischen Gesundheitsteam und EOL-Team nach Erkennung einer EOL-Situation **(4.)** - Sammeln von Basisinformationen (Prognose des*r Patient*in, Anzahl der Angehörigen, Welche Informationen haben die Angehörigen?, Kontaktinformationen der Angehörigen) - Phase 2: Soziale Bewertung **(6.)** - Telematischer Kontakt zwischen Sozialarbeiter*innen und Angehörigen - Soziale Bewertung (Unterstützungsnetzwerk des*r Patient*in, wichtigste Bezugspersonen, sozioökonomische Situation) - Vereinbarung eines Termins für einen Besuch im Krankenhaus - Phase 3: Psychologische Bewertung und Vorintervention **(6.),** - Persönliches Treffen des EOL-Teams mit den Angehörigen in der Krankenhaushalle - Erste grundlegende psychologische Beurteilung (Ermittlung des Bedarfs an psychologischer Unterstützung, emotionaler Zustand, emotionale Bedürfnisse, psychopathologischer Hintergrund) - Phase 4: Schlechte Nachrichtenübermittlung - Persönliches Treffen des EOL- und Gesundheitsteams mit den Angehörigen in einem privaten Raum **(6.),** - Übermittlung von schlechten Nachrichten (Erklärung der Entwicklung, Erläuterung der Prognose) - Psychologische Unterstützung (Förderung des emotionalen Ausdrucks, Förderung des Ausdrucks von Zweifeln und Ängsten in Bezug auf den Krankheitsverlauf und den Tod) - Erleichterung des Abschhieds, wenn Schwierigkeiten damit verbunden sind - Phase 5: Abschiednahme **(9.)** - Persönliche und private Verabschiedung im Patientenzimmer - Gewährleistung der Privatsphäre bei der Verabschiedung - Schutzmaßnahmen zur Vermeidung von Ansteckung vorsehen - Phase 6: Nach der Intervention **(10.)** - Persönliches Treffen des EOL-Teams mit den Angehörigen in einem großen privaten Raum - Psychologische Unterstützung (Förderung des emotionalen Ausdrucks, Validierung der Verlusterfahrung, der Erfahrung einen Sinn geben, gemeinsame Trauerreaktionen validieren, identifizierte Schutzfaktoren fördern, Psychoedukation über die Phasen der Trauer, Information über Trauerrituale und deren Anpassung an den COVID-19-Kontext, Ratschläge über die Übermittlung der Nachricht an Kinder, Spezifische Angsttechniken (z.B. Entspannung) **(6.)** - Abschließende psychologische Beurteilung (Schutzfaktoren: Unterstützung durch soziale Netzwerke, adaptive Reaktionen, Antizipation alternativer Trauerrituale; Risikofaktoren: psychopathologischer Hintergrund, andere Symptome, die auf eine besondere Aufmerksamkeit schließen lassen) **(6.)** - Soziale Informationen (Bestattungswesen, bürokratische Aspekte, öffentliche Beihilfen in sozioökonomischen Risikosituationen, spezialisierte Kontaktinformationen: Verweis auf spezifische Ressourcen wie z.B, EOL-Kontakt, Familienunterstützungsprogramm,, trauerspezifisches Programm) **(10.)** - (2) ) Familiäre Interventionen zur Mitteilung eines Verlustes an eine*n Patient*in: - Phase 1: Aktivierung und Koordinierung - Telematische Koordinierung zwischen dem Gesundheits- und EOL-Team nach der Feststellung des Todes eines nahestehenden kranken Menschen **(4.)** - Koordinierung des Informationsflusses zwischen Patient*innen, Gesundheitsteam und Angehörigen - Phase 2: Erstkontakt - Telematischer Kontakt zwischen dem EOL-Team und den Angehörigen **(4.)** - Bewertung der Bedürfnisse (Zweifel an Sicherheit im Krankenhaus; Zweifel, wie die schlechte Nachricht zu überbringen ist, Gefühlslage und Bedürfnisse) - Vereinbarung eines Termins für die Überbringung der schlechten Nachricht - Phase 3: Treffen mit dem EOL-Team - Persönliches Treffen des EOL-Teams mit dem Angehörigen in einem großen privaten Raum **(6.)** - Psychologische Unterstützung (Förderung des emotionalen Ausdrucks; Normalisierung der damit verbundenen Gefühle (z.B. Schuldgefühle); Beratung, wie die schlechte Nachricht zu überbringen ist; Informationen darüber, welche Reaktionen zu erwarten sind; Informationen über die Möglichkeit einer psychologischen Intervention beim Patienten) - Phase 4: Schlechte Nachrichtenüberbringung - Persönliche Übermittlung der schlechten Nachricht an Patient*in durch seinen Angehörigen im Patientenzimmer **(6.)** - Gewährleistung der Privatsphäre bei der Übermittlung schlechter Nachrichten - Schutzmaßnahmen zur Verhinderung einer Ansteckung vorsehen - Koordinierung mit Gesundheitsteam - Phase 5: Nach der Intervention **(6.)** - Angebot eines persönlichen Treffens mit dem EOL-Team für die Angehörigen - Psychologische Unterstützung - Nachbesprechung und Abschluss |
| Berthold D et al.; April 2020 | Deutschland | Empfehlung der Deutschen Gesellschaft für Palliativmedizin |  | Beschreibung der Aufgaben und Tätigkeitsfelder von Palliativpsycholog*innen im Rahmen der COVID-19-Pandemie | - Palliativpsychologen können einen wertvollen Beitrag zur Bewältigung der Herausforderungen der COVID-19-Pandemie leisten, indem sie: - Präsenz zeigen und sich als Unterstützer anbieten **(6.)** - Gefühlen Raum geben (ohne Wertung, ohne Ratschläge, ohne Analyse) **(6.)** - Unklare Situationen durch Begriffe begreifbar machen **(6.)** - Hilfreiche Metaphern nutzen **(6.)** - Vermittlung relevanter Informationen in einfacher, präziser und ruhiger Sprache **(6.)** - Katastrophierendes Denken relativieren, stattdessen Plan zur Krisenbewältigung **(6.)** - Anleitung von Achtsamkeits- und Entspannungsübungen (Entlastung) **(6.)** - Strukturierung des Alltags und Aufrechterhaltung von Routinen **(6.)** - Erarbeitung von Ressourcen zur Situationsbewältigung **(6.)** - Damit Palliativpatienten aufgrund der eingeschränkten Besuchsmöglichkeiten nicht in in ein Erleben sozialer Isolation kommen kann man folgende Interventionsmöglichkeiten einsetzen: **(3.)** - Erhöhter telefonischer Kontakt oder andere Formen der digitalen Kommunikation **(4.)** - Angebote in Form digitaler Medien (CD, eigene gemeinsam erarbeitete Imaginationsübungen, von Angehörigen aufgezeichnete Hörbücher) **(6.)** - Zu einem bestimmten Zeitpunkt „in Gedanken“ verabreden und einen Text „miteinander“ lesen **(6.)** - Den Angehörigen imaginativ „ans Bett holen“ **(6.)** - Hypnotherapeutische bzw. imaginative Interventionen z.B. „Was würde Ihre Tochter wohl sagen, wenn sie hier wäre? Wie würden Sie sich begegnen? Wo würde die Person sitzen?“ **(6.)** - COVID-19-Patient*innen sind belastet aufgrund der hohen situativen Ungewissheit (fehlende Informationen und Ansprechpartner*innen, Begrenzung von Personal etc.). Stärkung des Sicherheitsgefühls durch: - Orientierungshilfen in Zeit und Raum z.B. analoge Uhr organisieren mit großem Ziffernblatt **(6.)** - Imaginationsübungen z.B. innerer sicherer Ort **(6.)** - Übungen zur Erdung z.B. Symbolgegenstände oder -bilder (etwas von zu Hause, Glücksbringer) **(6.)** - Palliativpsycholog*innen können auf sensible Weise Informationen über den Zustand des Sterbenden an Angehörigen weitergeben.   Interventionsmöglichkeiten:   - Tonglen-Meditation (Transformation von Leid in Mitgefühl), sich im Herzen nah fühlen auch bei räumlicher Trennung **(6.)** - Rituale anregen, die die Liebe und das Wohlwollen ausdrücken **(6.)** - Eingehen auf Sinnfragen **(6.)** - Wenn sich Angehörige nicht mehr vom Verstorbenen verabschieden konnten aufgrund der Isolationsmaßnahmen, können Palliativpsycholog*innen als Vermittler*innen zwischen Klinik und Familie wirken, um ein Abschiednehmen trotz Isolation zu ermöglichen.   Interventionsmöglichkeiten: **(9.); (10.)**   - „Was hätte der Betroffene gerne noch gesagt? Was hätten Sie gerne noch gehört?“ **(10.)** - Einen fiktiven Brief an den Verstorbenen schreiben: „Wie hätte ich mir die Situation gewünscht?“ **(10.)** - Imaginationsarbeit: Sich an die letzte Berührung erinnern. Wie hat sich der Kontakt angefühlt? **(10.)** - Gibt es ein Foto vom Verstorbenen? Kliniken können angefragt werden, ob sie Foto des Verstorbenen machen. Das kann auch in Rituale und zum Abschiednehmen genutzt werden **(10.)** - Auf die Möglichkeit von Trauergruppen/-beratung hinweisen (Flyer in ausreichender Zahl bereithalten) **(10.)** |
| Blinderman C et al. 2021 | USA | Bericht eines Palliativdienstes |  | Überblick über klinische und gesundheitspolitische Maßnahmen eines Palliativteams während des Höhepunkts der COVID-19-Pandemie in New York City | - Palliativpflegeteam rief proaktiv Familienmitglieder an, um sie über den aktuellen Stand der Prognose und des medizinischen Managements zu informieren und ihnen die dringend benötigte unterstützende Beratung zukommen zu lassen **(4.)** - Ermöglichung virtueller Familienbesuche mit iPads am Patientenbett **(3.); (4.)** - Zentrale Rolle der Sozialarbeiter*innen und der Seelsorger*innen im Team bei der Unterstützung der Familie in ihrer Trauer und bei der Vermittlung von Gebeten und Trauergottesdiensten **(10.)** - Schulung der Ärzt*innen, die COVID-19-Patient*innen betreuen, durch Palliativversorgungsteam: Schulung konzentrierte sich auf (1) Familienkommunikation (2) Symptommanagement und (3) verfügbare Ressourcen für die Palliativversorgung. Die Informationen wurden per E-Mail und durch virtuelle Zoom-Präsentationen bei ärztlichen Führungstreffen verbreitet **(5.); (4.)** - Palliativteam der Notaufnahme führte Gespräche über Ziele der Versorgung mit Patient*innen und Familien und nutzte dabei die Gesprächsressourcen von VITALtalk COVID-19, die dazu anregten, klar und einfach zu kommunizieren, die Ziele und Werte der Patient*innen zu erkunden und Empfehlungen auf der Grundlage der angegebenen Ziele auszusprechen **(8.)** - Hospizstationen ermöglichten häufige Videobesuche der Familie und einen persönlichen Besuch der Familie vor dem Tod **(3.)** - Ruhige Umgebung auf Hospizstationen: Musik, die auf die individuellen Vorlieben der Patient*innen zugeschnitten sind, Kunstwerke, stimmungsvolle Beleuchtung und spirituelle Angebote **(6.)** - Fotos der Patient*innen am Bett angebracht **(6.)** - Künstlerische Darstellung der Eigenschaften, Vorlieben und Tugenden jedes*r Patient*in auf der Grundlage der von der Familie bereitgestellten Informationen erstellt und am Bett des*r Patient*in angebracht **(6.)** - die Familien teilten mit, dass diese Bemühungen ihnen halfen, das Leben ihres geliebten Menschen zu feiern und große Freude, Heilung und Seelenfrieden brachten **(6.)** - Um erhöhtes Beratungsaufkommen während Höhepunkt der Pandemie zu bewältigen: Erweiterung des Palliativteams durch Schulung anderer Mitarbeiter*innen (z.B. Assistenzärzt*innen der Psychiatrie, Dermatologe*innen, Geriater*innen, Sozialarbeiter*innen, Seelsorger*innen) in der Kommunikation mit Familienangehörigen und Rekrutierung freiwilliger Palliativmediziner*innen aus anderen Bundesstaaten **(2.), (5.)** - Schulung bestand aus schriftlichem Material und virtuellen Workshops zur Kommunikation, die auf COVID-19-Patient*innen zuschnitten waren **(5.)** |
| Bloomer M et al.; März 2021 | Australien | Diskussionspapier |  | Ziel der Stellungnahme ist es, Krankenschwestern und -pfleger*innen auf der Intensivstation eine praktische Hilfestellung zu bieten, um die Anwesenheit von Angehörigen bei Patient*innen, die an COVID-19 sterben, zu erleichtern | - Besuche sollte idealerweise auf eine Person beschränkt sein, die als nächster Angehöriger benannt wird **(3.)** - Ausgewählte Person sollte als gesund und fit gelten, sich nicht aufgrund einer COVID-19-Exposition selbst isolieren und derzeit nicht COVID-19-positiv sein **(3.)** - Beschränkungen der Besuchsdauer sollten erläutert werden **(3.)** - Wenn möglich, sollte der Sterbende in einem Einzelzimmer auf der Intensivstation untergebracht werden, um ein Höchstmaß an Privatsphäre für die Familie zu gewährleisten und Exposition gegenüber anderen Patient*innen zu begrenzen **(2.)** - Besuch sollte zu einem für beide Seiten günstigen Zeitpunkt angesetzt werden, wobei sicherzustellen ist, dass die Leitung der Intensivstation über den Besuch informiert ist und ein*e Mitarbeiter*in der Intensivstation zur Verfügung steht, um zu helfen **(3.)** - Besuch sollte mindestens 30 Minuten nach einem aerosolerzeugenden Verfahren angesetzt werden **(3.)** - Die Angehörigen sollten direkt zum Krankenhaus fahren, um die mögliche Exposition anderer zu begrenzen, einlagige Kleidung zu tragen, die heiß gewaschen werden kann, Schmuck ablegen und Wertsachen auf ein Minimum beschränken (z.B. nur Telefon und Autoschlüssel mitnehmen) **(3.)** - Bei der Ankunft sollten die Angehörigen darauf vorbereitet sein, was sie beim Betreten der Intensivstation sehen werden, was sie tun und was sie nicht tun dürfen **(3.)** - Die Angehörigen sollten angewiesen werden, Wertgegenstände abzuwischen und sich mindestens 20 Sekunden lang die Hände zu waschen **(3.)** - Mit Hilfe des Intensivpflegepersonals sollten die Angehörigen beim Anlegen der PSA (Kittel, OP-Maske, Schutzbrille und Handschuhe) unterstützt werden **(3.)** - Die Angehörigen sollten angewiesen werden, die Vorderseite der Maske zu keinem Zeitpunkt während des Besuchs abzunehmen oder zu berühren **(3.)** - Wenn der Tod unmittelbar bevorsteht und ein Besuch innerhalb von 30 Minuten nach einem aerosolerzeugenden Verfahren zwingend erforderlich ist, müssen die Angehörigen eine N95-Maske tragen (anstelle einer chirurgischen Maske) **(3.)** - Wenn möglich, sollten die Angehörigen Zeit mit der sterbenden Person allein verbringen können, wobei sie angewiesen werden sollten, die Rufglocke zu benutzen, um das Personal um Hilfe zu bitten **(3.)** - Nach Beendigung des Besuchs sollte der*die Mitarbeiter*in der Intensivstation den Angehörigen helfen, die gesamte PSA abzulegen und sicherstellen, dass sie ordnungsgemäß entsorgt wird **(3.)** - Die Angehörigen sollten angewiesen werden, sich die Hände zu waschen, die Station zu verlassen und direkt nach Hause zu gehen (3.) - Nach der Rückkehr nach Hause sollte der*die Besucher*in angewiesen werden, seine Kleidung in einer heißen Waschmaschine zu waschen **(3.)** - Falls erforderlich, kann der*die für die Betreuung des*r Besucher*in zuständige Mitarbeiter*in der Intensivstation sofortige emotionale Unterstützung leisten. Der*die Besucher*in sollte auch über Unterstützungsdienste informiert werden, die ihm zur Verfügung stehen, wie z.B. Sozialarbeiter*innen, Seelsorger*innen oder der Beratungsdienst, der über den Gesundheitsdienst oder die örtlichen Gemeindedienste verfügbar ist **(3.); (6.)** |
| Bolt SR et al.; Januar 2021 | Niederlande | Übersichtsarbeit |  | Formulierung von Praxisempfehlungen für das Pflegepersonal im Hinblick auf die Bereitstellung palliativer Demenzpflege in Zeiten von COVID-19 | Advance Care Planning   - Rechtzeitige Gespräche zur Vorausplanung der Pflege einleiten, indem das Thema COVID-19 sorgfältig eingeführt wird. Pflegekräfte könnten erklären, dass es im Rahmen von COVID-19 ratsam ist, mögliche (realistische) Szenarien und Optionen für die Pflege am Lebensende proaktiv zu besprechen, um unerwünschte Behandlungen (wie unerwünschte Krankenhauseinweisungen, lebenserhaltende Behandlungen oder Krankenhausaufenthalte) zu vermeiden **(7.); (8.)** - Es sollte ein Folgegespräch oder ein Anruf geplant werden, um auf psychosoziale oder spirituelle Bedürfnisse oder Fragen einzugehen, die sich aus der Diskussion über die Pflegeplanung ergeben können **(7.); (8.)** - Pflegepersonal sollte mit den Patient*innen mit Demenz den bevorzugten Sterbeort besprechen, bevor es zu einer COVID-19-Ansteckung kommt. Eine vertraute Umgebung wird wahrscheinlich einem Krankenhaus vorgezogen **(7.); (8.)**   Körperliche Aspekte der Pflege   - Angebot zusätzlicher Unterstützung und Erklärungen bei der richtigen Handhygiene für demenzkranke Bewohner*innen **(7.)** - Anbringen demenzfreundlicher Hinweistafeln oder Schilder in den Badezimmern oder an anderen Stellen, um daran zu erinnern, sich die Hände 20 Sekunden lang mit Seife zu waschen **(7.)** - Demonstration, wie man sich gründlich die Hände wäscht **(7.)** - Verwendung von Handdesinfektionsmitteln oder antibakteriellen Handtüchern als Alternative für Menschen mit Demenz, die zum Händewaschen nicht ohne weiteres an ein Waschbecken gelangen können **(7.)** - Ermutigung von Menschen mit Demenz in ein Taschentuch zu niesen und zu husten (und es anschließend zu entsorgen) oder in den Ellbogen statt in die Hände **(7.)** - Erinnerung der demenzerkrankten Menschen, sich sozial und körperlich zu distanzieren und Ermutigung dieser in ihrem Zimmer zu bleiben, z.B., indem sie mit Indoor-Hobbys gelockt werden **(7.)** - Veränderungen des Gesundheitszustands, der Stimmung oder des Verhaltens gelten als potenzielle Indikatoren für eine Infektion und sollten sorgfältig dokumentiert und besprochen werden, wobei zu berücksichtigen ist, dass Menschen mit Demenz solche Veränderungen möglicherweise nicht selbst melden **(7.)** - Für Menschen mit Demenz, die an COVID-19 erkranken, sollte anstelle der Isolation in einem eigenen Zimmer die Einrichtung von COVID-19-Kohorten in Betracht gezogen werden, die Bewegungsfreiheit ermöglichen **(7.)**   Psychologische Aspekte der Pflege   - Beachtung der Tatsache, dass Regelungen zur sozialen Isolation und Distanzierung, gefolgt vom erlebten Verlust sozialer Beziehungen und dem Anblick der persönlichen Schutzausrüstung des Personals bei Menschen mit Demenz Gefühle von Angst, Depression, Trauer und Trauma auslösen können **(7.)** - Es sollte versucht werden, die erhöhte Angst vor COVID-19 bei Menschen mit Demenz zu verringern, durch: - Zugang und Exposition gegenüber Medieninformationen minimieren **(7.)** - Demenzerkrankten Menschen sollten Informationen und Erklärungen gegeben werden, wenn sie Bedenken äußern **(7.)** - Einfache Erinnerungshilfen und visuelle Anweisungen verwenden, um die aktuelle Situation zu erklären **(7.)** - Verwenden einer beruhigenden Sprache und Gesten, um Menschen mit Demenz zu helfen, die Sicherheitsvorschriften zu befolgen **(7.)** - Es sollte häufig mit demenzerkrankten Menschen gesprochen werden und gefragt, wie es ihnen geht, und es sollte sich Zeit genommen werden ihnen zuzuhören. Die Gefühle der Person sollten bestätigt werden und sie beruhigt werden **(7.)** - Negative Ausdrücke wie „Krise“, „Pandemie“, „Abriegelung“ oder „Isolierung“ sollten vermieden werden und es sollten mit Kolleg*innen nicht über COVID-19-Vorschriften in Gegenwart von Personen mi Demenz diskutiert werden **(7.)** - Ausreichend Zeit mit Demenzkranken verbringen und sie ermutigen, sich an alltägliche Aktivitäten und Routinen zu beteiligen, um Langeweile und Verwirrung zu vermeiden **(7.)** - Verhaltensweisen, die eine Gefahr für die Betroffenen selbst oder für andere darstellen können, sollten gemeldet werden (z.B. das Ignorieren oder Widersetzen von Sicherheitsvorkehrungen) **(7.)** - Es sollte nach psychosozialen Interventionen zur Bewältigung solcher Verhaltensweisen gesucht werden: - Verwendung alter Fotos, Gegenstände oder Lieder aus der Vergangenheit, um sich abzulenken **(7.); (6.)** - Förderung der Bewegung, Sport und (kreative oder haushaltsnahe) Tätigkeiten (z.B. Malen, Kochen, Handtücher falten) **(7.); (6.)** - Einhaltung eines regelmäßigen Zeitplans und Routine **(7.)** - Suche nach Familienmitgliedern, die (Video-)Anrufe tätigen können, um durch Interaktion die Ängste und die Verwirrung ihres demenzkranken Angehörigen zu verringern **(7.); (6.)** - Pflegepersonal sollte mit psychiatrischen Fachkräften und Sozialarbeitern zusammenarbeiten, um eine angemessene psychiatrische Versorgung zu gewährleisten **(7.); (6.)**   Soziale Aspekte der Pflege   - Reduktion sozialer Isolation und Einsamkeit durch: - Ermutigung von Familien und Freunden, Briefe, Zeichnungen oder andere Pakete vorbeizubringen **(7.); (6.)** - Ermutigung der Demenzkranken zur Teilnahme an alltäglichen Aktivitäten (z.B. Musik oder Hörbücher hören) **(7.); (6.)** - Ermutigung (individuell, aus der Ferne) zum Singen, Spazierengehen im Freien oder zu sportlichen Aktivitäten **(7.); (6.)** - Für soziale Unterstützung sorgen, indem regelmäßige Besuche von Familienmitgliedern arrangiert werden **(7.); (6.)** - Pflegen (kleiner) Gruppenaktivitäten, wenn möglich; spielen interaktiver Spiele über Overhead-Lautsprecher, abwechselnd zu den Mahlzeiten essen (7.); (6.) - Förderung des Einsatzes von Technologie, wie z.B. Tablets oder Smartphones, um das soziale Online-Engagement (mit Angehörigen) zu erleichtern. Beachten dabei, dass der Einsatz von Technologie möglicherweise zusätzliche Erklärungen und Unterstützung bedarf und möglicherweise nicht für jeden geeignet ist **(7.); (6.); (4.)** - Investition in kreativen Lösungen und Alternativen für die eingeschränkten Besuchsmöglichkeiten **(7.); (6.)** - Erwägung des Einsatzes fortschrittlicher Technologien wie Virtual Reality, um Menschen mit Demenz in Isolation die Möglichkeit zu bieten, sich mit ihrer Familie an einem simulierten, vertrauten Ort zu treffen oder besondere Umgebungen zu besuchen (z.B. Musikkonzerte, Naturexpeditionen) **(7.); (6.); (4.)** - Pflegepersonal kann sich dafür einsetzen, den pflegenden Angehörigen die Möglichkeit zu bieten, während der COVID-19-Periode einzuziehen **(7.); (6.)**   Spirituelle Aspekte der Pflege   - Es ist ratsam, bei Gesprächen über die Vorausplanung der Pflege auf persönliche Werte, gewünschte Rituale oder spirituelle Praktiken im Zusammenhang mit dem Lebensende zu achten (was auch Pläne für die Beerdigung oder das Gedenken einschließen kann) **(7.); (8.)** - Erwägung der Hinzuziehung eines*r Seelsorger*in oder spirituellen Betreuer*in, um Menschen mit Demenz am Lebensende und ihre Familie während des Sterbeprozesses zu unterstützen **(7.); (6.)**   Pflege von Sterbenden   - Pflegepersonal kann bei Person mit Demenz am Lebensende dafür plädieren, den Familien die Möglichkeitder persönlichen Abschiednahme zu geben, trotz möglicher Besuchsverbote aufgrund von COVID-19 **(7.); (9.)** - Wenn Familienmitglieder nicht in der Lage sind, Sterbenden persönlich zu besuchen, sollte digitale Technologien genutzt werden, um Verbindung zwischen dem Sterbenden und seiner Familie herzustellen **(7.); (4.)** - Familie in jeden Schritt einbeziehen und fragen nach Ideen, wie ein friedlicher, auf die sterbende Person zugeschnitter Tod gestaltet werden kann **(7.); (9.)** - Auf Rituale achten, die es Menschen mit Demenz und ihren Familien ermöglichen, sich angemessen zu verabschieden: **(7.); (9.)** - Rituale für den Abschied am Ende des Lebens sollten auf die Person zugeschnitten und sich an der spezifischen Lebensauffassung oder Religion der sterbenden Person orientieren **(7.); (9.)** - Sprechen mit der sterbenden Person, auch wenn diese kognitive Beeinträchtigungen hat und sich der Situation nicht bewusst ist. Z.B. der Person erklären, dass der Moment gekommen ist, sich zu verabschieden und versuchen dabei, ein Gefühl der Ruhe und des Friedens zu bewahren **(7.); (9.)** - Anzünden einer Kerze und eine Schweigeminute einlegen **(7.); (9.)** - Mit sterbender Person über ihre Familienmitglieder und andere Personen sprechen, mit denen sie in Verbindung stand. Bitten, dass die sterbende Person z.B. an diese Person denken soll oder sich diese Person vorstellen soll und fragen, was sie ihnen sagen möchte. Wenn eine Person nicht mehr sprechen kann, sollte Pflegepersonal eigene Gedanken und Gefühle mitteilen -> dadurch kann das Gefühl der Verbundenheit gefördert werden, auch wenn keine Angehörigen anwesend sind **(7.); (9.)** - Wenn Familienmitglieder anwesend sind, entweder persönlich oder per Videoanruf, können diese gemeinsames Gefühl der Verbundenheit fördern (z.B. durch das Lesen eines Gedichts, gemeinsames Schweigen, gemeinsame Gebete, singen oder summen, Musik hören) **(7.); (9.)**   Sterbebegleitung   - Information von Familienmitgliedern, falls sie zum Zeitpunkt des Todes nicht anwesend waren, über den Sterbeprozess und die Rituale, die zur Förderung eines friedlichen Abschieds eingesetzt wurden. Erzählen von bedeutungsvollen letzten Worten oder Gesten ihres Angehörigen -> dies kann Angehörigen helfen, Verlust auf gesunde Weise zu betrauern **(10.)** - Erwägen, ein würdiges Foto des Demenzkranken nach dem Tod mit den Hinterbliebenen zu teilen (mit deren Erlaubnis) -> da Angehörige zum Zeitpunkt des Todes nicht anwesend waren, kann dies der einzige visuelle Beweis für den Tod der Person sein und eine emotionale Entlastung ermöglichen **(10.)** - Pflegepersonal sollte Familien erklären, dass Trauer normal ist und im Allgemeinen Zeit braucht: **(10.)** - Familienmitgliedern sagen, dass sie an das Wesentliche denken sollen und sich gesund ernähren, ausreichend trinken, regelmäßig Sport treiben und versuchen, ihren gewohnten Tagesablauf beizubehalten **(10.)** - Familienmitgliedern sagen, dass sie (digital) in Kontakt bleiben sollten und mit anderen darüber sprechen, wie es ihnen geht **(10.)** - Familienmitgliedern sagen, dass sie sich erlauben sollten, negative Gefühle zu erleben und dass es Zeit braucht, einen Verlust zu verarbeiten **(10.)**   Ethische Aspekte der Pflege   - Um mit Stress und Verlust während der COVID-19-Pandemie umzugehen, sollten die Pflegekräfte die folgenden Tipps zur Selbstfürsorge beachten: - Trennen vom Katastrophenereignis, indem man sich gelegentlich Pausen (und tiefe Atemzüge) gönnt **(6.)** - Vergewissern, dass man lokale Ressourcen und Dienste kennt, an die man sich für zusätzliche Unterstützung wenden kann **(6.)** - Man sollte sich einer Gruppe von Gleichgesinnten anschließen und um angemessene Betreuung bitten **(6.)**   Struktur und Prozesse der Pflege   - Bewohner*innen mit COVID-19-Symptomen müssen sofort in einem separaten Zimmer oder auf einer COVID-19-Kohorteneinheit isoliert werden **(1.)** - Pflegepersonal kann sich für Umsetzung strenger Maßnahmen zur Infektionsprävention in seiner Einrichtung einsetzen (z.B. Tragen von persönlicher Schutzausrüstung wie Masken, Schutzbrillen, Kittel und Handschuhen) **(1.)** - Es ist ratsam, dass sich Pflegekräfte, die mit infizierten Personen in Kontakt waren, bei Auftreten von Symptomen testen lassen und sich selbst in Quarantäne begeben, falls sie positiv getestet werden **(1.)** - Um die Gefahr einer Ausbreitung des Virus zu verringern, ist es ratsam, dass das Pflegepersonal nicht in mehreren Einrichtungen arbeitet und die Bewegung zwischen den Einrichtungen einschränkt **(1.); (2.)** - Das Pflegepersonal kann mit spezialisierten (mobilen) Palliativpflegeteams zusammenarbeiten, falls vorhanden, um die notwendige Unterstützung und Pflege für Menschen mit Demenz zu gewährleisten **(7.)** - Das Pflegepersonal kann sich für die Anpassung von Informationsmaterialien (wie Leitlinien und Informationsblätter), die für die Allgemeinbevölkerung entwickelt wurden, an Menschen mit kognitiven und verhaltensbedingten Beeinträchtigungen einsetzen oder persönlich dazu beitragen **(7.)** - Das Pflegepersonal kann sich für die Einrichtung einer 24-Stunden-Hotline für Palliativpflege einsetzen, um Menschen mit Demenz und ihren Familien Ratschläge und Antworten zu geben **(7.)** |
| Boufkhed S et al.; September 2020 | Afrika | Empirische Arbeit | Online-Querschnittsbefragung | Bewertung der Bereitschaft und Fähigkeit afrikanischer Palliativdienste, auf die COVID-19-Pandemie zu reagieren | - Umgang mit Personalstress durch: Verfügbarkeit eines Programms zur Unterstützung des Personals, Beratung oder Diskussionen in Teamsitzungen **(6.)** - Schulung der Mitarbeiter*innen im Umgang mit hochinfektiösen Krankheiten wie COVID-19, wobei eine Hälfte vor der Pandemie und eine Hälfte als Reaktion auf COVID-19 geschult worden ist **(5.)** - Reinigungspersonal wurde in den Informationsaustausch und die Schulung zum Umgang mit COVID-19 einbezogen **(5.)** - In überwiegender Mehrheit der Dienste wurden zusätzliche Handwaschvorrichtungen eingeführt **(1.)** - Von 28 Diensten gaben 19 an, einen Isolierraum für COVID-19-Fälle eingerichtet zu haben **(2.)** - Am häufigsten genannten Mittel für den Informationsaustausch mit dem Personal waren WhatsApp/Viber und Telefonanrufe sowie Telefongespräche mit Patient*innen und Angehörigen oder Besucher*innen **(4.)** - Andere Kommunikationsmittel: persönliche Gespräche, Plakate oder Aushänge in der Einrichtung sowie Radio oder andere Medien **(4.)** |
| Boufkhed S et al.; Februar 2021 | Afrika und Naher Osten | Empirische Arbeit | Online-Querschnittsbefragung | Bewertung der Bereitschaft und Kapazität von Palliativdiensten im Nahen Osten und in Nordafrika zur Reaktion auf die COVID-19-Pandemie | Maßnahmen zur Infektionskontrolle und Ressourcen   - Mit einer Ausnahme verfügten alle Teilnehmer*innen über Handwaschvorrichtungen an den Eingängen **(1.)** - Vier von fünf verfügten über zusätzliche persönliche Schutzausrüstung für Palliativpflegepersonal und Reinigungspersonal und wiesen einen Isolierraum aus **(1.)** - Die Befragten hatten Personal, das im Umgang mit hochinfektiösen Erkrankungen geschult war, von denen die Hälfte vor COVID-19 geschult wurde **(5.)**   Informationssysteme, Kommunikation und Technologie   - Fast alle Dienste verfügte über aktuelle Patienten- und Mitarbeiterlisten und erfassten Symptome, Ergebnisse und Behandlung der Patient*innen **(4.)** - Zwei von fünf Diensten erfassten jedoch keine Kontaktdaten oder Besuchsdaten von Besucher*innen und Angehörigen **(4.)**   Palliativpflegepersonal und Fachwissen zur Unterstützung der Pandemiebekämpfung   - Etwa die Hälfte der Dienste verfügte über Pläne zur Umschichtung von Personal **(2.)** |
| Cherniwchan HR; August 2021 | Kanada | Übersichtsarbeit |  | Sammlung relevanter Informationen über die virtuelle stationäre Palliativversorgung und dessen Einsatz während der COVID-19-Pandemie, um eine bessere operative Anleitung für Palliativpflegedienste bei Pandemien zu entwickeln | 1. Allgemeine Akzeptanz der virtuellen stationären Palliativversorgung während der Pandemie  - Meisten Mitarbeiter*innen und Patient*innen bestätigen in Artikeln, dass die virtuelle stationäre Palliativversorgung eine akzeptable Alternative zu persönlichen Diensten ist (Akzeptanz zwischen 70 und 100%) **(4.)** - Denn die virtuelle stationäre Palliativversorgung verbessert nachweislich die Sterbe- und Todeserfahrung von Patient*innen und ihren Familien während der Pandemie, indem sie die Isolation sicher überbrückt und das Gefühl der Selbstbestimmung erhöht **(4.)** - Die meisten Patient*innen waren jedoch nach wie vor der Ansicht, dass die Technologie nicht die tatsächliche Anwesenheit eines Familienmitglieds bei der klinischen Visite ersetzen könnte **(4.)** - Die virtuelle Palliativversorgung ermöglicht auch einen leichteren Zugang zu den Patient*innen für das Personal. Mitarbeiter*innen, die sonst durch Kinderbetreuungspflichten eingeschränkt wären, können von zu Hause aus arbeiten **(4.)** - Virtuelle Palliativpflegeplattformen schaffen auch Raum für virtuelle Selbsthilfegruppen für trauernde Familien **(4.)** - Virtuelle Palliativpflege spart PSA ein **(4.)**  1. Wichtige logistische Überlegungen bei der Entwicklung einer Plattform für virtuelle Palliativversorgung  - Psychosoziale und spirituelle Angebote wie geführte Meditationen, Chöre und Yoga, die zusätzlich zu regelmäßigen Arzt- und Familiengesprächen angeboten werden **(4.); (6.)** - Ehrenamtliche Aktivitäten wie Live-Geschichtenerzählen, Freundschaftsanrufe und Bastelsitzungen sowie Trauerbegleitung in Gruppen sollten für die virtuelle pädiatrische Hospizversorgung in Betracht gezogen werden **(4.); (6.)** - Es wird empfohlen, dass Kliniker bei der Durchführung von E-Meetings aus Gründen der Qualitätssicherung einheitliche Rahmenbedingungen einhalten, z.B. wichtigsten Fertigkeiten bei der Durchführung von Gesprächen für schwere Krankheiten: **(4.)** - Richtiger Aufbau: Führung des Gesprächs in einem gut beleuchteten, privaten Raum, wobei der Kopf und das obere Drittel des Oberkörpers auf dem Bildschirm zu sehen sein sollten, Blickkontakt gehalten werden sollte und ein professioneller Hintergrund verwendet werden sollte **(4.)** - Aufrechterhaltung des Gesprächsrhythmus: Vermeidung überflüssigen Redens **(4.)** - Reaktion auf Emotionen: Einfühlungsvermögen und aufmerksames Zuhören zeigen, indem Hand auf das Herz gelegt wird, sanft genickt und sich zur Kamera geneigt wird **(4.)** - Abschluss des Besuchs: Zusammenfassung des Besuchs und Klärung von letzten Fragen **(4.)** - Festlegung einer einzigen Kontaktperson in der Familie, mit der die Meetings geplant werden und die Bereitstellung direkter Meeting-Anweisungen per E-Mail **(4.)**  1. Häufig verwendete Technologien für die Bereitstellung virtueller Plattformen  - Beschaffung von Tablets mit Installation von Video-Telehealth-Software **(4.)** - Tablets wurden am häufigsten verwendet, um videofähige virtuelle klinische Konsultationen und Familienbesuche durchzuführen. Die Patient*innen wurden entweder mit ihren eigenen Tablets ausgestattet oder es wurden gemeinsame Tablets zwischen den Zimmern getragen **(4.)** - Schulung des klinischen Personals in Bezug auf Humanisierung von Videobehandlungen **(5.)** - Smartphone-gestützte Telepalliativpflege-Modelle: i.d.R. Verwendung der eigenen Smartphones der Patient*innen **(4.)** - In einigen Studien wurden zwar gängige kommerzielle Lösungen wie WhatsApp, FaceTime und Zoom verwendet, aber diese Plattformen sind möglicherweise nicht kompatibel mit den Cybersicherheitsbereichen von Krankenhäusern. Spezielle HC-Plattformen sind erforderlich, wie z.B. Attend Anywhere. Diese Plattform verhindert die Aufzeichnung des Bildschirms durch den*die Empfänger*in und bietet mehrere programminterne Sprachübersetzungen, um schwierige Gespräche mit Patient*innen zu erleichtern, deren Muttersprache nicht die gleiche ist wie die des Pflegepersonals **(4.)** - Einsatz virtueller Realität (VR), um eine friedliche oder vertraute Umgebung zu erleben oder zu Reisezielen reisen, die auf der „Bucket List“ stehen **(4.)** - Projizierung der Patient*innen in einem hologrammähnlichen Format für trauernde Familien in der Ferne **(4.)** - Ausrüstung ist jedoch teuer und benötigt eine stabile Breitband-Internetverbindung **(4.)**  1. Strategien für die Nutzung von Personalressourcen zur Bewältigung des gestiegenen Patientenaufkommens  - Unterstützung durch Fachkräfte des Gesundheitswesens aus weniger betroffenen Gebieten des Landes **(2.)** - Rekrutierung Freiwilliger für die Telepalliativversorgung über Social-Media **(2.)** |
| Chomton M et al.; Februar 2021 | Frankreich | Bericht eines Kinderkrankenhauses |  | Beschreibung der Erfahrungen bei der Umwandlung einer pädiatrischen Intensivstation in eine Erwachsenen-Intensivstation für COVID-19-Patient*innen | - Erhöhung der Bettenkapazität für Erwachsene innerhalb einer Woche von acht auf zwölf und dann auf 18 **(2.)** - Aufrechterhaltung eines Unterdrucks in allen Zimmern, Ausstattung jedes Zimmers mit einer Luftschleuse und Einrichtung getrennter sauberer und schmutziger Kreisläufe **(2.)** - Es kamen sechs Anästhesisten zu den zehn leitenden Intensivmediziner*innen dazu, die normalerweise auf der pädiatrischen Intensivstation tätig waren. Außerdem kamen zwölf Assistenzärzte für Pädiatrie und Anästhesiologie zu den zehn Mitarbeiter*innen der pädiatrischen Intensivstation dazu **(2.)** - Über eine Videoverbindung konnten die Mitarbeiter*innen Ratschläge von Ärzt*innen aus der Erwachsenen-Medizin einholen **(2.)** - Vier vollzeitbeschäftigte Physiotherapeut*innen boten Massagen, passive und aktive Bewegungsübungen, Atemphysiotherapie, Unterstützung der Patient*innen beim Aufstehen und Aufsitzen aus dem Betten und Schluckuntersuchungen an **(6.)** - Das mobile pädiatrische Palliativpflege- und Betreuungsteam des Krankenhauses kümmerte sich persönlich um die Patient*innen und ihre Angehörigen, denen der Besuch der Intensivstation untersagt war. Ein spezieller Raum außerhalb des Krankenhausgebäudes wurde genutzt, um die Angehörigen in einer sicheren und beruhigenden Umgebung zu empfangen **(2.)** - Die Angehörigen erhielten eine sichere E-Mail-Adresse und sie schickten Kinderzeichnungen, Familienfotos, Musikstücke und Gebete, was das Personal an die Patient*innen übermittelte **(4.); (6.)** - Ein*e Mitarbeiter*in rief die Familie einmal täglich an, um ihr medizinische Informationen über ihren Angehörigen zu geben **(4.)** - Es wurden Arbeitsgruppen gebildet, die sich mit verschiedenen Themen wie Protokollen für die persönliche Schutzausrüstung (PSA) und die Bauchlage beschäftigten. Innerhalb von 7 Tagen wurde diese Protokolle sowie Video-Tutorials, Checklisten und schnelle Simulationssitzungen dem gesamten Personal zur Verfügung gestellt **(5.)** - Einer der Ärzt*innen führte dreimal wöchentlich eine einstündige Videokonferenz mit einem*r Ärzt*in der Intensivstation für Erwachsene, um sich über die neusten Empfehlungen auf dem Laufenden zu halten. Die Ergebnisse dieser Gespräche wurden an das gesamte Personal weitergeleitet **(4.); (5.)** |
| Cohen-Mansfield J; Dezemeber 2020 | Israel | Positionspapier |  | Darstellung der Probleme von Pflegeheimen während der COVID-19-Pandemie und Vorschläge zur Verbesserung der Pflege | - Personal kann aus der arbeitslosen Bevölkerung oder anderen Freiwilligen rekrutiert werden. Dabei sollte es sich um Personen ohne Risikofaktoren handeln, die mit Schutzausrüstung ausgestattet werden **(2.)** - Das neue Personal muss vom vorhandenen Personal angeleitet werden **(5.)** - Erstellung eines Aktionsplans in Zusammenarbeit mit den Mitarbeiter*innen zum Schutz des Personals und der Bewohner*innen vor einer Ansteckung: Ein solches Programm muss beispielsweise Maßnahmen beinhalten, die sicherstellen, dass nicht dasselbe Personal sowohl gesunde als auch infizierte Bewohner*innen behandelt und dass das Personal, das infizierte Bewohner*innen behandelt, vollständig geschützt ist. Das Programm müsste so konzipiert werden, dass die physische Trennung zwischen infizierten und nicht-infizierten Bewohner*innen bis zum Zeitpunkt der Genesung maximiert wird **(2.)** - Sowohl für das Personal als auch für die Bewohner*innen sollten regelmäßige Tests und eine tägliche Infektionsüberwachung durchgeführt werden **(1.)** - Dem gesamten Personal und den Bewohner*innen, für die dies angemessen und notwendig ist, muss eine geeignete Schutzausrüstung zur Verfügung gestellt werden **(1.)** - Die Öffentlichkeit, das Heimpersonal und die Behörden müssen verstehen, dass nicht alle älteren Menschen in ihren Zimmern isoliert werden können. Personen mit Demenz z.B., die die meiste Zeit umherwandern, müssen die Möglichkeit haben, dies zu tun und dürfen nicht physisch zurückgehalten werden **(7.)** - In Langzeitpflegeeinrichtungen sollte Personal eingestellt werden, das die Kommunikation zwischen den Bewohnern und ihren Familien mit technischen Mitteln (WhatsApp, Zoom, Hangouts, Skype, usw.) ermöglicht **(2.); (4.)** - Nicht-infizierten Familienmitgliedern sollte der Besuch gestattet werden, v.a. wenn der Angehörige krank ist. Solche Besuche erfordern geeignete Schutzmaßnahmen für Besucher*innen, Personal und Bewohner*innen **(3.)** - Es sollten neue Wege gefunden werden, um Gruppenaktivitäten, die für das Wohlbefinden von Menschen mit Demenz entscheidend sind, sicher zu gestalten **(7.)** - Ein spezieller Fernsehkanal mit Inhalten, die für ältere Erwachsene geeignet sind, insbesondere für diejenigen, die ihr Langzeitgedächtnis behalten, sollte in verschiedenen Sprachen rund um die Uhr an sieben Tagen in der Woche eingerichtet werden **(6.)** |
| Costantini M et al.; April 2020 | Italien | Empirische Arbeit | Telefonische Querschnittsbefragung | Untersuchung der Vorbereitung auf und der Auswirkungen der COVID-19-Pandemie auf Hospize in Italien, um andere Länder zu informieren und deren Umgang mit der Pandemie zu erleichtern | - Alle Hospize haben ihre Besucherpolitik geändert: - Zwölf haben Regel verfolgt, nur einen Angehörigen pro Patient*in zuzulassen. Zwei dieser Hospize waren bereit, diese Politik zu lockern, wenn Patient*innen im Sterben lagen, während eines nur Besucher*innen zuließ, wenn Patient*innen im Sterben lagen **(3.)** - Ein Hospiz verlangte, dass Besucher*innen Tag und Nacht im Hospiz bleiben mussten und nicht zurückkehren durften, wenn sie das Hospiz verlassen hatten und zwei waren für Besucher vollständig geschlossen **(3.)** - In zwei Hospizen wurden Verwandte vor dem Betreten des Hospizes auf Symptome untersucht **(3.)** - Die Betreuung nach dem Tod war unterschiedlich. Vier Hospize schränkten die Zahl der Angehörigen, die den Leichnam des*r verstorbene*n Patient*in besichtigen konnten, ein. Ein Hospiz hatte allen Angehörigen den Zutritt zur Leichenhalle verboten und ein anderes hatte ein System eingeführt, bei dem Angehörige den Leichnam des Verstorbenen durch ein Fenster betrachten konnten **(3.); (9.)** - Änderungen in der Praxis: Ein Hospiz, in dem die Besuche stark eingeschränkt waren, hatte ein System eingeführt, bei dem der*die Hospizpsycholog*in täglich mit den Angehörigen der Patient*innen telefonierte, um sie auf den neuesten Stand zu bringen und ihnen psychologische Unterstützung zukommen zu lassen **(4.); (6.)** - Andere Hospize hatten alle internen Sitzungen sowie den Jahresurlaub gestrichen **(2.)** |
| Davies A, Hayes J; Mai 2020 | Vereinigtes Königreich | Übersichtsarbeit |  | Ausgabe von Vorschlägen für die Bereitstellung von Palliativpflege während einer Pandemie | - Patient*innen am Lebensende benötigen Einfühlungsvermögen und „sichere“ persönliche Interaktionen (insbesondere in Abwesenheit der Familie) **(6.)** - Es sollte sich so viel wie möglich auf den*die Patient*in eingelassen werden: **(6.)** - Verbale Kommunikation, d.h. Erklärungen, allgemeines „Geplauder“ **(4); (6.)** - Nonverbale Kommunikation, z.B. Daumen hoch/runter **(4); (6.)** - Angemessene (sichere) Berührung, z.B. Hand halten **(6.)** - Sicherstellen, dass grundlegende Pflegebedürfnisse erfüllt werden **(6.)** - Erleichterung des Fernkontakts mit der Familie, z.B. durch Mobiltelefone **(4.)** - Erleichterung des direkten Kontakts mit der Familie bei Patient*innen am Lebensende **(6.)** - Regelmäßige Kommunikation mit der Familie **(4.)** - Änderungen der Umgebung in Betracht ziehen (um die Erfahrung des*der Patient*in zu verbessern) **(6.)** - Erwartungen steuern, z.B. bevorzugter Ort der Pflege/des Sterbens **(8.)** |
| Delis S et al.; März 2020 | Deutschland | Empfehlung der Deutschen Gesellschaft für Palliativmedizin |  | Ausgabe von Handlungsempfehlungen zur Sicherstellung der Palliativversorgung in Zeiten der COVID-19-Pandemie | Maßnahmen zum Erhalt der Arbeitsfähigkeit der Teams:   - Regelmäßige Anpassung des Hygienefahrplans nach RKI **(1.)** - Vermeidung der Versammlung des Teams in engen Räumen **(1.)** - Nutzung von Telemedizin, Telefon **(4.)** - Einrichtung spezifischer Telefonsprechstunden für Angehörige, die aufgrund von Isolations- und Quarantänemaßnahmen nicht bei Besuchen/Kontakten präsent sein können **(4.)** - Reduktion auf eine Begleitperson bei Visiten/Kontakten und eine*n Repräsentant*in bei Familienkonferenzen/Angehörigengesprächen **(3.)** - Regelung der Besuchszeiten auf Palliativstation entsprechend den Vorgaben bzw. Klärung, welche Ausnahmeregeln es geben kann **(3.)**   Behandlungsplan um COVID-19 spezifische Aspekte erweitern:   - Überprüfung der Therapiezieldefinition **(8.)** - Klärung der Indikationen und des Einverständnisses für spezifische Maßnahmen **(8.)** - Festlegung des geeigneten/gewünschten Behandlungsorts **(8.)** - Anleitung zur Umsetzung des Behandlungsplans/vorausschauende Verschreibung für zu erwartende Symptome **(8.)** - Absprachen mit Angehörigen zu telefonischer Beratung und Auskunft als Kompensation für nicht stattfindende persönliche Kontakte **(4.); (8.)** |
| Dhahri A et al.; Februar 2021 | Vereinigtes Königreich | Bericht eines Krankenhauses |  | Ermittlung der Auswirkungen des virtuellen Besuchs auf das Personal und Angehörige von Patient*innen | - Der virtuelle Besuch wurde in Form eines Videoanrufs zwischen einem*r COVID-19-Patient*in und einem Familienmitglied oder einer nahestehenden Person im Beisein eines*r Mitarbeiter*in unter Verwendung einer cybersicheren, benutzerfreundlichen Software, Attend Anywhere (AA), auf einem digitalen Gerät (iPad) durchgeführt **(3.); (4.)** - Familienangehörige, die ihren Verwandten noch am selben Tag sehen wollten, konnten über eine von einem Verwaltungsteam geleitete E-Mail einen virtuellen Besuch anfordern **(3.); (4.)** - Freiwillige aus einem Pool von Krankenhausmitarbeiter*innen und Medizinstudierenden wurden rekrutiert **(2.)** - Nutzung von Krankenhaus-iPads für Videogespräche **(4.)** - Die Mitarbeiter*innen erhielten ein Skript, in dem ihre Rolle und die Durchführung der Anrufe beschrieben waren **(5.)** - Die Freiwilligen waren verpflichtet, sich über den Zustand des*der Patient*in zu informieren, bevor der*die Patient*in zum virtuellen Besuch kam **(3.)** - Im Anschluss an das Gespräch trugen die Freiwilligen die Daten des virtuellen Besuchs in die integrierten Gesundheitsakten ein **(3.)** - Die Besucher*innen und das Personal wurden aufgefordert, nach jedem Anruf ein E-Mail-Feedback in Form einer Reflexion ihrer Erfahrungen zu geben **(4.)** |
| Dunleavy L et al.; März 2021 | Vereinigtes Königreich | Empirische Arbeit | Multinationale Online-Querschnittsbefragung von Anbietern spezialisierter Palliativversorgung | Darstellung und Verständnis von innovativen Ansätzen und Praxisänderungen spezialisierter Palliativdienste als Reaktion auf COVID-19 | - 27,6% der Befragten meldeten eine Erhöhung der Bettenanzahl **(2.)** - Einige Einrichtungen haben zwei Zonen eingerichtet: Zonen, in denen Menschen mit vermuteter oder bestätigter COVID-19-Infektion betreut werden und eine Zone für Menschen ohne Verdacht auf COVID-19 **(2.)** - Erstellung von Leitfäden unter der Leitung des Oberarztes zur Kommunikation mit Angehörigen am Telefon **(4.)** - 83,7% der Dienste berichteten, dass sie virtuelle Technologie mit Patient*innen und Angehörigen während des Zeitraums der COVID-19-Pandemie sehr viel mehr oder etwas mehr einsetzten als vor der Pandemie. Für die Kommunikation wurden allgemeine digitale Plattformen wie Zoom, Skype, WhatsApp und Facebook genutzt **(4.)** - IPads wurden durch Wohltätigkeitsspenden über die Facebook-Seite der Einrichtung aufgetrieben und ermöglichen es vielen Familien, mit ihren Angehörigen zu sprechen oder sie auch nur zu sehen **(4.)** - Verwendung von Postkarten für E-Mail und Telefonnachrichten von Angehörigen, die den Patient*innen übergeben oder vorgelesen werden können **(4.); (6.)** - Anwendung verschiedener Strategien, um das Wohlbefinden des Personals zu fördern: kostenlose Parkplätze; kostenlose Mahlzeiten; Hilfe bei der Kinderbetreuung; virtuelles Yoga; Umfunktionierung des Hospizklinikraums in Personalraum mit weichen Möbeln und Handcremes, handgefertigten Peeling-Taschen, handgefertigten Halterungen für Masken, um Hautreizungen zu vermeiden, Schokolade usw. **(6.)** |
| Etkind SN et al.; Juli 2020 | Vereinigtes Königreich | Übersichtsarbeit |  | Zusammenfassung der Erkenntnisse über die Rolle und die Reaktion von Palliativpflege- und Hospizteams auf Pandemien/Epidemien | - Beschränkung der Besucherstunden/Zahlen **(3.)** - Änderung der Zulassungskriterien **(2.)** - Einführung von Systemen der täglichen telefonischen Unterstützung für Familien **(4.); (6.)** - Beendigung von Freiwilligendiensten **(2.)** - Einführung von Palliativpflegeprotokollen für nicht spezialisiertes Personal zur Behandlung von Symptomen und psychologischer Unterstützung **(2.); (6.)** - Schulung für Standortleiter in der Anwendung der Protokolle **(5.)** - Aus- und Fortbildung für nicht spezialisiertes Personal in den Grundlagen der Palliativpflege, einschließlich Kommunikation und Trauerbegleitung **(5.)** - Identifizierung eines*r Entscheidungsträger*in zur Verbesserung der Kommunikation **(4.)** - Einschränkung des Kontakts mit Freiwilligen zur Infektionskontrolle **(1.)** - Einbeziehung von Seelsorger*innen in die Reaktion auf die Pandemie **(6.)** - Maßnahmen zur Verbesserung der Verbundenheit unter den Mitarbeiter*innen **(6.)** - Verstärkte Maßnahmen zur Unterstützung des Gesundheitspersonals im Umgang mit Stress **(6.)** - Virtuelle Technologie zur Ermöglichung der Kommunikation, dort wo der Besuch eingeschränkt ist, z.B. Bereitstellung eines täglichen Updates für Familien **(4.)** |
| Florencio R et al.; Oktober 2020 | Brasilien | Übersichtsarbeit |  | Analyse der wissenschaftlichen Erkenntnisse über die Umsetzung der Palliativmedizin während der COVID-19-Pandemie | - Um sozialer Isolation entgegenzuwirken, sollte das Personal den Patient*innen Videoanrufe zur Verfügung stellen, die eine Kommunikation mit den Angehörigen ermöglichen **(4.); (6.)** - Viele ziehen es vor, in ihrem familiären Umfeld zu sterben, statt auf einer Intensivstation. In solchen Fällen sollte der Respekt vor den Wünschen der Patient*innen und ihrer Familie im Vordergrund stehen **(8.)** |
| Flores S et al.; November 2020 | USA | Bericht einer Notaufnahme |  | Beschreibung des Einsatzes von Telepalliativmedizin in einer Notaufnahme, um Familienmitgliedern die Möglichkeit zu geben, während einer kritischen Krankheit und sogar am Lebensende mit ihren Angehörigen zu kommunizieren | - Angesichts des zunehmenden Bedarfs an Palliativmedizin während der COVID-19-Pandemie bei gleichzeitiger Begrenzung des Einsatzes von PSA führte die Notaufnahme Telemedizin-Dienste ein **(4.)** - Ein COVID-19-Palliativteam, das sich aus einem*r behandelnden Ärzt*in, einem*r Sozialarbeiter*in, einer Krankenschwester und einem*r Krankenhausseelsorger*in zusammensetzte, war unter der Woche 12 Stunden am Tag persönlich und in der Nacht und am Wochenende per Telefon oder Telemedizin-Beratung erreichbar **(4.)** - Nutzung von Telemedizin durch Notärzt*innen und Palliativmediziner*innen für Gespräche mit Familienmitgliedern. Alle Mitglieder des Pflegeteams hatten Möglichkeit, Telemedizin über spezielle iPhones und iPads/Tablets zu nutzen **(4.)** - Einsatz von Videokommunikation ermöglicht es Familienmitgliedern, Angehörige zu sehen **(4.); (6.)** - Durch Telemedizin konnte COVID-19-Palliativteam Fernkonsultationen durchführen, insbesondere außerhalb der Arbeitszeiten, v.a. an Wochenenden und während der Nachtschichten. Dies ermöglichte ein geringeres Infektionsrisiko bei gleichzeitiger Aufrechterhaltung einer hochwertigen Versorgung **(4.)** - Verkürzung der Zeitspanne der sozialen Distanz: Telemedizin ermöglicht Kontakt mit Familie und in einigen Fällen einen Abschluss für schwerkranke Patient*innen. Patient*innen am Ende ihres Lebens mit Angehörigen in Kontakt treten **(4.); (6.)** - Um Kommunikation zu verbessern, sollte die eingesetzte Technologie mit Audio-Dolmetscherdiensten integriert werden **(4.)** - Am wichtigsten ist, sicherzustellen, dass der Empfang oder die Internetverbindung während einer Ferninteraktion nicht unterbrochen wird und der Ton klar ist **(4.)** |
| Gergerich E et al.; November 2020 | USA | Empirische Arbeit | Qualitative Studie | - Erforschung der Erfahrungen von Hospiz-Sozialarbeiter*innen und Patient*innen in der Frühphase der COVID-19-Pandemie in den USA - Ausgabe von Empfehlungen für die Praxis der Sozialarbeit | Vorschläge für die Praxis der Sozialarbeit   - Ergebnisse der Studie zeigen, dass einige Hospizpatient*innen keinen Zugang zu der für die Teilnahme an der Telemedizin erforderlichen Technologie haben. Dazu können ein Smartphone, ein Tablet, ein Computer mit Webcam, eine zuverlässige Internet- oder Mobilfunkverbindung gehören. Sozialarbeiter*innen sollten eine Bewertung des technologischen Zugangs, der Fähigkeiten und der Präferenzen der Familien vornehmen **(4.)** - Der*die Sozialarbeiter*in sollte Ressourcen ermitteln, um Patient*innen und Familien bei der Beschaffung von Technologien für die Telemedizin zu unterstützen, wenn sie keinen Zugang haben und dies als notwendig erachtet wird **(4.)** - Für Krankenhäuser und Pflegeheime wird es wichtig sein, ein System für die Bereitstellung von Technologie und technischer Unterstützung einzurichten, damit die Patient*innen mit Angehörigen kommunizieren können, die sie nicht in der Einrichtung besuchen können **(4.)** - Sozialarbeiter*innen sollten bei der Kommunikation mit Patient*innen und deren Angehörige klärende Fragen stellen, um sicherzustellen, dass sie diese richtig verstehen **(4.)** - Verwendung und Bereitstellung durchsichtiger Gesichtsmasken kann Kommunikation zwischen Patient*in und Fachkraft erleichtern **(4.)** - Wenn Patient*innen mit Fachkräften konfrontiert werden, die unter der PSA nicht zu erkennen sind, kann Erstellung von Profilen der Fachkräfte auf der Website der Agentur oder die Bereitstellung von Einführungsunterlagen für Patient*innen dazu beitragen, die Beziehung und den Kontakt zu verbessern **(4.)** - Hospiz-Sozialarbeiter*innen sollten die Patient*innen und ihre Familien bei der Erstellung individueller Notfallpläne unterstützen und diese Pläne in regelmäßigen Abständen mit der Familie überprüfen **(6.)** |
| Griffin KM et al.; April 2020 | USA | Erfahrungsbericht eines Krankenhauses |  | Beschreibung der Verfahren und Prozesse, die während eines einmonatigen Zeitraums entwickelt wurden, während die Notfallplanung lief und der Bedarf an Intensivpflegekapazitäten exponentiell anstieg. Diese Ansätze bieten einen potenziellen Fahrplan für Zentren, die sich schnell an die enormen Herausforderungen dieser und potenzieller künftiger Pandemien anpassen müssen | - Epidemiologisches Personal des Krankenhauses klärte das Personal in den COVID-19-Abteilungen rasch über das optimale Format für das An- und Ablegen der PSA auf **(5.)** - Krankenhausweite Verteilung von Online-Videos und Aufklärungsmaterialien für Mitarbeiter*innen **(5.)** - In jedem Patientenzimmer befinden sich spezielle Smartphones mit Ladegeräten, so dass das Personal innerhalb des Zimmers zusätzliche Mitarbeiter*innen außerhalb des Zimmers kontaktieren kann, ohne die PSA ablegen zu müssen **(4.)** - Durch die eingeschränkten Besuchsmöglichkeiten ist die Kommunikation mit den Angehörigen komplex und bruchstückhaft geworden. Um diesen Aspekt der Patientenversorgung zu berücksichtigen, wird eine begrenzte Anzahl von Patientenvertreter*innen benannt, die mit den Ärzt*innen kommunizieren. Darüber hinaus wird jedem Familienmitglied ein zweiter unterstützender Anruf von einem Mitglied des Palliativteams angeboten. Bei drohendem Tod oder unmittelbar nach dem Tod wird einem einzelnen Familienmitglied in PSA die Möglichkeit eines Besuchs gegeben. Wenn ein Besuch nicht möglich ist, kann das Personal Videokonferenzen einsetzen, um den Angehörigen die Möglichkeit zu geben, ihr sterbendes Familienmitglied zu sehen und mit ihm zu sprechen **(3.); (4.)** - Um die Personalbesetzung zu erleichtern, wurden Krankenpfleger*innen, die zuvor in der Einrichtung gearbeitet haben, kontaktiert und ihnen eine beschleunigte Neuzulassung angeboten, sofern sie dazu bereit und verfügbar waren **(2.)** - Dem gesamten Personal wurde eine angemessene PSA zur Verfügung gestellt **(1.)** - In Zusammenarbeit mit der Abteilung für Infektionskontrolle und -prävention haben das Krankenhaus und die medizinische Fakultät für eine angemessene Aufklärung über das An- und Ablegen der PSA gesorgt **(5.)** - Kolleg*innen in der Notfall- und Krankenhausmedizin wurden dazu ermutigt, frühzeitig und ehrlich mit Patient*innen und Familien über die Ziele der Versorgung und die Klärung von Patientenverfügungen zu sprechen. Ein Programm, das 24 Stunden am Tag Zugang zu Palliativmediziner*innen und Ethikberater*innen bietet, hat dazu beigetragen, diese Gespräche zu erleichtern **(8.)** - Einsatz psychologischer Fachkräfte, die den Mitarbeiter*innen Einzel- und Gruppenunterstützung bei der Bewältigung von Traumata und akutem Stress anbieten **(6.)** - Je nach Bedarf können Überweisungen an psychologische Unterstützungsdienste gegeben werden **(6.)** |
| Haire E et al.; Juli 2021 | Vereinigtes Königreich | Empirische Arbeit | Quantitative Studie | Erfassung der Erfahrungen und Aktivitäten von Sterbebegleiter*innen während der COVID-19-Pandemie sowie Skizzierung von Empfehlungen für diese Rolle in künftigen Diensten und Schulungen | - Der Begleitdienst bestand aus Fachkräften des Gesundheitswesens, die für den Trust tätig waren und sich freiwillig für diese Aufgabe gemeldet hatten, nachdem ihr normales Arbeitspensum aufgrund von COVID-19 reduziert worden war **(2.)** - Die Freiwilligen bekamen gezielte Schulung durch den*die leitende*n Seelsorger*in und das spezialisierte Palliativversorgungsteam des Trusts **(5.)** - Jeder Tag wurde in drei vierstündige Schichten aufgeteilt, die auf freiwilliger Basis vergeben wurden **(2.)** - Das spezialisierte Palliativversorgungsteam wählte geeignete Patient*innen aus und ein Mitglied des Teams leitete jede Übergabe **(2.)** - Die Begleitpersonen übernahmen eine Vielzahl von Aktivitäten: - Kommunikation (Über Erinnerungen und Interessen sprechen, virtuelle Besuche ermöglichen, Angehörige auf dem Laufenden halten oder Briefe von geliebten Menschen vorlesen) **(4.); (6.)** - Spiritualität und Literatur (Lesen eines Buches oder einer Zeitung, Abspielen von Musik oder Ermöglichen eines Besuchs des Kaplans) **(6.)** - Gesellschaft leisten (Reden, die Hand halten oder in geselliger Stille bei ihnen sein) **(6.)** |
| Halek M und Holle D; Oktober 2020 | Deutschland | Empfehlungen der Deutschen Gesellschaft für Pflegewissenschaft e.V. |  | Ausgabe von Empfehlungen zur sozialen Teilhabe und Lebensqualität in der stationären Altenhilfe in Zeiten von COVID-19 | - Die Einrichtung sollte einen Pandemieplan erstellen, der die Wahrung der Würde der Person mit Pflegebedarf in den Mittelpunkt stellt **(1.)** - Den Bewohner*innen sollte frühzeitig die Gelegenheit gegeben, ihren medizinischen Behandlungspräferenzen zu äußern **(8.)** - Gesundheitliche Versorgungsplanung (Advance Care Planning (ACP) **(8.)** - Zu einer Vorsorgeplanung gehört eine individuell zugeschnittene Beratung zu medizinisch-pflegerischen, psychosozialen und/oder seelsorgerischen Angeboten in der letzten Lebensphase **(8.)** - Liegt bereits eine Verfügung/Beschreibung der Behandlungspräferenzen vor, die vor COVID-19-Erkrankung verfasst wurde, ist die Relevanz für diesen Fall zu prüfen. Verfügung ist hinsichtlich der Gültigkeit mit Betroffenen bzw. Interessenvertreter*innen zu diskutieren **(8.)** - Die inhaltliche Ausgestaltung und Dauer der Quarantäne sollte auf Basis einer individuellen Risikoeinschätzung erfolgen **(1.)** - Dauer und Ausprägung von Quarantänemaßnahmen sind vor dem Hintergrund der Risiken und Nachteile individuell zu bestimmen - Die Bewohner*innen erhalten individuell abgestimmte Angebote zur Beziehungsgestaltung - Einrichtungen sollten dauerhaft alternative Kontaktmöglichkeiten für Bewohner*innen anbieten, um negative Folgen (z.B. soziale Isolation) zu reduzieren **(4.)** - Falls keine erhöhte Gefahr besteht (z.B. Symptomfreiheit bei Pflegebedürftigen und ihre Angehörigen) ist Berührung bei Besuchen mit Angehörigen zu gestatten. Hier ist neben Bereitstellung geeigneter Schutzausrüstung nach alternativen Lösungen zu suchen. Stellt z.B. Berührung in Handschuhen Hürde dar, kann u.U. sorgfältige Handhygiene Alternative sein, um körperliche Berührung zu ermöglichen **(1.); (3.)** - Kontakt der Bewohner*innen mit Personen außerhalb der Einrichtung sollte aufrechterhalten werden und virtuelle Besuche von Angehörigen per Telefon oder Videokonferenz organisiert werden **(4.); (3.)** - In Zusammenarbeit mit Kirchengemeinden regelmäßige Gottesdienste, religiöse Angebote via Videostream oder durch Videokonferenzen gemeinsame Gebete mit Seelsorger*innen organisieren **(4.); (6.)** - Übertragung von Angeboten (u.a. Konzerte, Vorstellungen) auf die Fernsehgeräte in den Zimmern **(4.)** - Personal der einzelnen Wohnbereiche voneinander trennen und kleinere feste Bewohnergruppen bilden, die in konstanter Zusammensetzung und Einhaltung von Hygienemaßnahmen gemeinsame Aktivitäten durchführen, damit bei Nachweisen von SARS-CoV-2 nur eine kleine Gruppe von Personen als Kontakte entsteht **(4.)** - Für den Einzelfall alternative Lösungen anbieten, sodass sich auch Bewohner*innen untereinander unter Einhaltung der Schutz- und Hygienemaßnahmen besuchen und gemeinsam Zeit verbringen **(2.)** - Besuche von Familie, Freunden, wichtigen Bezugspersonen, ehrenamtlichen Hospizhelfern etc. ermöglichen **(3.)** - Einheitliche Dauer und Anzahl der Besuche ist nicht gerechtfertigt **(3.)** - Besucher*innen mit Erkältungssymptomen sollen Einrichtung aber fernbleiben **(1.); (3.)** - Die Kontaktpersonen sollten ihre verbale und nonverbale Kommunikation aufgrund des Mund-Nasenschutzes anpassen **(4.)** - Deutlicher, langsamer und/oder lauter sprechen, gestische Kommunikation verstärkter anwenden oder mit Symbolkarten arbeiten **(4.)** - Kurzfristiges Abnehmen des MNS unter Wahrung des 1,5m Abstands, um z.B. Gesicht zur Begrüßung zu zeigen, einer Emotion Ausdruck zu verleihen oder wichtige Informationen deutlicher zu kommunizieren, kann eine angemessene Lösung sein **(4.)** - Die Bewohner*innen sollten Angebote zur sinnstiftenden Alltagsgestaltung erhalten **(6.)** - Digitale Angebote für Unterhaltung und Ablenkung **(4.); (6.)** - Gottesdienste, Konzerte vor dem Gebäude stattfinden lassen, an dem Bewohner*innen am Fenster oder Balkon teilnehmen können **(6.)** - Kommunikation innerhalb der Einrichtung: Bewohner*innen, Mitarbeiter*innen und Angehörige sollten Informationen zur Bewältigung der Pandemie erhalten **(4.)** - Leicht verständliche, leicht zugängliche, klare Informationen **(4.)** - Ggf. in unterschiedlichen Sprachen **(4.)** - Kommunikationsinhalte: aktuelle Informationen über das SARS-CoV-2-Virus und COVID-19-Erkrankung; Information und Beratung zu aktuellen Schutzmaßnahmen innerhalb der Einrichtung; aktuelle Lageberichte der Einrichtung (z.B. aktuelle Infektionsfällen); Informationen zu Möglichkeiten der Kommunikation innerhalb der Einrichtung; Informationen zum aktuellen Tagesablauf innerhalb der Einrichtung (z.B. Essenszeiten, Angebote an sozialen Aktivitäten, Möglichkeiten der Bewegungsförderung); Information im Kontext existenzieller Lebenssituationen (z.B. Pflege von sterbenden Menschen) **(4.)** - Kommunikation ist so zu gestalten, dass die Mitarbeiter*innen motiviert werden und positive Aspekte in Bezug auf Zeit nach der Pandemie erkennbar sind **(4.); (6.)** - Mitarbeiter*innen erhalten niedrigschwellig kurzfristige, an lokale Situation angepasste Schulungs- und Informationsangebote in personenzentrierter Kommunikation **(5.)** - Kommunikation nach außen: Externe Netzwerkpartner*innen, Dienstleister*innen und Leistungserbringer*innen, Aufsichtsbehörden und Vertreter*innen des jeweiligen Quartiers sollten Informationen zur Bewältigung der Pandemie erhalten **(4.)** - Aktuelles zum Pandemieplan **(4.)** - Information zur aktuellen Versorgung, z.B. wie erfolgt Kontakt mit externen Leistungserbringern, wie erfolgt Essensaufnahme, welche sozialen Aktivitäten werden angeboten etc. **(4.)** - Information über Ansprechpartner*innen innerhalb der Einrichtung für die Kommunikation von und nach außen **(4.)** - Die Arbeits- und Verantwortungsbereiche sowie die Versorgungsprozesse für die Umsetzung des Pandemieplans sollten für die Mitarbeiter*innen einer Einrichtung klar definiert werden **(4.)** - Eindeutige Beschreibung der Arbeits- und Verantwortungsbereiche **(4.)** - Tägliches Gesundheitsscreening der Mitarbeitenden ggf. „Gesundheitstagebuch“ für Mitarbeiter*innen, um Exposition zu erfassen **(1.)** - Begrenzung des Einsatzes von Personal mit Gesundheitsrisiken **(1.)** - Führungsverantwortlichen und das Steuerungsteam sollten gemeinsam eine Atmosphäre des Vertrauens und der Wertschätzung schaffen **(6.)** - Die Führungsverantwortlichen und das Steuerungsteam sollten sensibilisiert sein für die psychischen Belastungen der Mitarbeiter*innen **(6.)** - Informationsangebot für die Mitarbeiter*innen sollte Hinweise zur Selbstfürsorge, für den Umgang mit Stress und psychischen Belastungen sowie zu verfügbaren psychosozialen Unterstützungsangeboten enthalten **(6.)** - Es wird empfohlen, die Mitarbeiter*innen auf folgende Strategien für den Umgang mit Stress hinzuweisen: - Das Erleben vermehrten Stresses während der Pandemie ist verständlich und kein Hinweis darauf, den Aufgaben nicht gewachsen zu sein **(6.)** - Es ist wichtig, auf die Einhaltung der Grundbedürfnisse (Pausen, Ernährung, körperliche Aktivität, Schlaf) und bisheriger Routinen im Alltag zu achten **(6.)** - Ungesunde Bewältigungsstrategien sollten vermieden und stattdessen auf früher bereits als hilfreich wahrgenommene Strategien zurückgegriffen werden **(6.)** - Der Austausch mit Kolleg*innen sollte gesucht und die persönlichen sozialen Kontakte (Familie und Freunde) sollten aufrechterhalten werden **(6.)** - Starke emotionale Reaktionen sollten zugelassen werden **(6.)** - Es sollte zur Einholung sozialer Unterstützung bei länger anhaltendem Stress und beeinträchtigtem psychischen Wohlbefinden ermutigt werden, verbunden mit Hinweisen auf einrichtungsinterne und -externe Ansprechpersonen und Unterstützungsangebote **(6.)** - Allen Mitarbeiter*innen sollten einrichtungsinterne und -externe Informations- und Beratungsangebote zur psychosozialen Unterstützung zur Verfügung stehen **(6.)** - Formelle einrichtungsinterne Unterstützungsangebote umfassen z.B. benannte Vertrauenspersonen, die niedrigschwellig und ggf. auch anonym von den Mitarbeitenden kontaktiert werden können sowie speziell für die psychosoziale Beratung qualifizierte Mitarbeitende **(6.)** - Formelle externe psychosoziale Unterstützungsangebote können professionelle Beratungs- und Therapieangebote sein, die niedrigschwellig zugänglich sind **(6.)** - Der Personaleinsatz sollte darauf ausgerichtet werden, Belastungsspitzen für Mitarbeiter*innen zu vermeiden und ausreichend Zeit für Erholung zu ermöglichen **(6.)** - Weniger erfahrene Mitarbeitende können durch erfahrenere unterstützt werden **(6.)** - Einhaltung von Pausenzeiten ist durch die Führungs- und Teamkommunikation zu unterstützen **(6.)** - Arbeitszeiten möglichst flexibel den Bedürfnissen der Mitarbeiter*innen anpassen und ausreichend lange arbeitsfreie Zeiträume einräumen **(6.)** - In den Einrichtungen sollte für die Bewohner*innen, die Angehörigen, die Mitarbeiter*innen sowie für alle weiteren externen Besucher*innen ausreichende Schutzausrüstung zur Verfügung **(1.)** - Die Mitarbeiter*innen sollten niedrigschwellige Schulungen zur personenzentrierten Umsetzung der Schutzmaßnahmen erhalten **(5.)** - Wissen zur Bedeutung von sozialer Isolation, Kontaktabnahme, Langeweile, Einsamkeit und Angst im Zusammenhang mit der Pandemie sowie Strategien zur Prävention und Minimierung dieser Folgen **(5.)** - Verständnis von personenzentrierter Pflege unter den Bedingungen einer Pandemie **(5.)** - Wissen und Fertigkeiten der pflegerischen Diagnostik (Erfassung, Analyse und Bewertung) von möglichen infektionsrelevanten Sachverhalten sowie von bewohnerindividuellen Bedürfnissen und Bedarfen unter Pandemie-Bedingungen **(5.)** - Wissen zu Hauptwegen und Ursachen für SARS-CoV-2-Übertragungen **(5.)** - Wissen zu Hygieneregeln und Fertigkeiten zur Umsetzung dieser **(5.)** - Wissen zur palliativen Pflege im Kontext von Pandemien **(5.)** - Fertigkeiten im Umgang mit digitalen Anwendungen und digitalen Kommunikationsstrukturen **(5.)** - Wissen und Fertigkeiten für Ethische Reflexion konflikthaltiger Situationen **(5.)** - Wissen zur Konzeption zur Prävention von Personalmangel **(5.)** - Angebot von Hands-on-Training, Nutzung audiovisueller Medien (z.B. Filme), Bereitstellung von Checklisten **(5.)** |
| Hart JL et al.; August 2020 | USA | Übersichtsarbeit |  | Beschreibung eines Rahmens für die familienzentrierte Pflege im Kontext der COVID-19-Pandemie und Darstellung eines Werkzeugkastens mit Strategien zur Umsetzung im stationären Bereich | - Der*die Patient*in und die Familie sollten über alle restriktiven Maßnahmen aufgeklärt werden, die die physische Anwesenheit von Familienmitgliedern einschränken **(5.)** - Da Familien am Eintrittspunkt oft nur begrenzten persönlichen Kontakt haben, sollte eine öffentlich zugängliche Website zusätzliche Informationen liefern. Die Erläuterung der Maßnahmen sollte eine Begründung enthalten und eine Sprache, die darauf abzielt, Konflikte zu vermeiden **(5.)** - Die Website sollte auch Links zu Ressourcen in der Gemeinde, kostenlosen oder kostengünstigen öffentlichen Internetprogrammen und Informationen über die bevorzugten Kommunikationsplattformen des Gesundheitssystems enthalten **(5.); (4.)** - Das klinische Team sollte darauf abzielen, 1) einen vom Patienten benannten Hauptansprechpartner in der Familie zu bestimmen, der idealerweise, aber nicht notwendigerweise, der*die rechtliche Entscheidungsträger*in des*r Patient*in in der Gesundheitsversorgung ist, 2) die dem*r Patient*in und der Familie zur Verfügung stehenden Kommunikationstechnologien zu dokumentieren und 3) alle Barrieren für die Kommunikation und das Engagement zu identifizieren und zu entschärfen **(4.)** - Familienmitglieder ohne Internetzugang oder videokonferenzfähiges Gerät: - Telefone und Telefonkonferenzen einsetzen **(4.)** - Bereitstellung eines vom Krankenhaus zur Verfügung gestellten Telefons mit kostenlos ausgehenden Anrufen für Patient*innen **(4.)** - Patient*in ohne videokonferenzfähiges Gerät: - Ermöglichung des Zugangs zu Videokonferenzen über ein krankenhauseigenes Gerät **(4.)** - Die Familienmitglieder sprechen nicht dieselbe Sprache wie das klinische Team: - Zugang zu Übersetzungsdiensten bei Videokonferenzen oder Telekonferenzen **(4.)** - Familienmitglieder oder Patient*innen haben nur begrenzte technische Kenntnisse - Bereitstellung von Anweisungen für die Nutzung der bevorzugten Videokonferenzplattform, die auf alle technologischen Kompetenzniveaus zugeschnitten sind **(4.)** - Einsatz von Telefon- und Telefonkonferenzen **(4.)** - Patient*in hat keine Kommunikationshilfen wie Brillen oder Hörgeräte: - Erleichterung der Übergabe wichtiger Gegenstände von der Familie an den*die Patient*in **(2.)** - Student*innen der Medizin, der Krankenpflege oder der Sozialarbeit, die nicht im klinischen Praktikum sind, können qualifizierte Unterstützung leisten und gleichzeitig ihre eigene Ausbildung und ihre Fähigkeiten verbessern **(2.)** - Ermutigung des*r Patient*in und seiner Familie, so oft wie gewünscht miteinander zu telefonieren, zu schreiben und Videokonferenzen abzuhalten **(4.)** - Erleichterung der Bereitstellung von Kommunikationsgeräten, einschließlich Ladegeräten, von der Familie für den*die Patient*in **(4.)** - Bereitstellung eines kostenlosen Internetzugangs für stationäre Patient*innen und Unterstützung dabei ihre persönlichen Geräte anzuschließen **(4.)** - Verwendung von Freisprecheinrichtungen, um die Kommunikation zwischen Familienmitgliedern und Patient*innen zu erleichtern **(4.)** - Angebot von Hilfe für Patient*innen, die Audio-, Video- oder schriftliche Nachrichten an Familienmitglieder schicken möchten **(4.)** - Nutzung von Videokonferenzen, einschließlich der Verwendung krankenhauseigener Geräte durch Fenster oder Türen für Patient*innen auf der Isolierstation, um den Familienmitgliedern ihren Angehörigen und die Umgebung zu zeigen **(4.)** - Lesen, drucken oder spielen von Nachrichten der Familie an den*die Patient*in **(4.)** - Unterstützungsangebot für Gebete oder Ermöglichung des*r Patient*in, Gebete oder Gottesdienste von externen Glaubensvertretern per Videokonferenz zu besuchen **(6.)** - Schaffung eines Systems, um begrenzte persönliche Gegenstände in das Patientenzimmer liefern zu lassen, wie z. B. Kunstwerke der Kinder, Sporterinnerungsstücke oder religiöse Gegenstände **(2.)** - Anpassung der Umgebung des*r Patient*in, nachdem von den Familienmitgliedern erfahren wurde, welche Speisen, Musik, Hörbücher und Fernseher bevorzugt werden **(6.)** - Kontaktaufnahme mit der Familie zum Zeitpunkt der Verlegung oder Aufnahme, um den*die Hauptansprechpartner*in, den*die gesetzlichen Entscheidungsträger*in im Gesundheitswesen und einen Kommunikationsplan festzulegen **(4.)** - Definition und Dokumentation des Plans für den täglichen Kontakt mit der Familie, einschließlich des zuständigen klinischen Teammitglieds **(4.)** - Tägliche Videokonferenzen (oder telefonischer Kontakt) mit einem*r Hauptansprechpartner*in der Familie als Standard, sofern nicht anders gewünscht **(4.)** - Dokumentation der täglichen Kommunikation für Transparenz, Verantwortlichkeit und Konsistenz **(4.)** - Möglichst einheitliche Ansprechpartner*innen für die Familienangehörigen **(4.)** - Bitten der Familienmitglieder, die vergangenen Zeiten und die Lebensgeschichte des*r Patient*in zu beschreiben, einschließlich wichtiger Personen in ihrem Leben, um das Gespräch zwischen dem klinischen Team und dem*r Patient*in zu erleichtern **(4.)** |
| Hofmeyer A und Taylor R; Januar 2021 | Vereinigtes Königreich | Diskussionspapier |  | Identifizierung von Strategien und Ressourcen, die Führungskräfte in der Pflege nutzen können, um die Mitarbeiter*innen mit Empathie und Umsicht durch die COVID-19-Pandemie zu führen | - Führungskräfte können ihre Teams auf drei Arten wirksam motivieren: **(6.)**  1. Richtungsanweisung: Führungskräfte müssen Zweck der Organisation klären und Schritte zur Lösung von Problemen und Herausforderungen aufzeigen **(6.)** 2. Sinnstiftung: Führungskräfte müssen erklären, welche Maßnahmen erforderlich sind, um das Ziel zu erreichen **(6.)** 3. Einfühlungsvermögen: Führungskräfte müssen eine emotionale Sprache verwenden, um die Probleme und Herausforderungen anzuerkennen, mit denen die Mitarbeiter*innen konfrontiert werden und emotionale Unterstützung und Anleitung bieten **(6.)**  - Führungskräfte müssen in offenen Gesprächen mit ihren Mitarbeitenden auf moralisch herausfordernde Themen und das Risiko einer moralischen Verletzung (moral distress) bei der Pflege von Patient*innen mit COVID-19 aufmerksam machen **(6.)** |
| Hower KI et al.; August 2020 | Deutschland | Empirische Arbeit | Online-Befragung von Leitungskräften ambulanter und stationärer Pflege- und Hospizeinrichtungen | Untersuchung der Wahrnehmung der Herausforderungen der COVID-19-Pandemie durch Leitungskräfte von Pflegeeinrichtungen und ihrer Bewältigungsstrategien im Umgang mit der Pandemie | Vorsorgemaßnahmen   - Strenge Einhaltung und Kontrolle des Kontaktverbots und der Hygienemaßnahmen **(1.)** - Schaffung von Ressourcen zur Separation und Versorgung infizierter Personen (u.a. Aufnahmestopp, Schaffung einer Isolationsstation) **(2.)** - Weitreichender Einsatz von Desinfektionsmitteln **(1.)** - Entwicklung von Maßnahmen zur Aufrechterhaltung des Betriebs bei Personalausfall **(2.)**   Materielle Ausstattung   - Ausstattung der Mitarbeitenden mit Behelfsmasken und Händedesinfektionsmitteln **(1.)** - Rationierung und kontrollierte Ausgabe von Schutzausrüstung **(2.)** - Eigenproduktion von Schutzausrüstungen (u.a. MNS, Gesichtsschutz) **(1.); (2.)** - Herstellung/(teure) Beschaffung von Desinfektionsmitteln **(1.); (2.)** - Beschaffung von Anti-Körper-Tests für die Mitarbeitenden **(1.); (2.)**   Strukturelle Strategien und Maßnahmen:   - Verringerung von Ansteckungswegen (u.a. Bildung kleinerer Teams, Umstrukturierung des Dienstplans, Verzicht auf Dienstbesprechungen und Visiten, betriebsinternes Pandemiehandbuch, Isolation verschiedener Bereiche) **(2.)** - Vermeidung von Personalengpässen (u.a. Einsatz von Zeitarbeitspersonal, Einsatz von Ehrenamtlichen, Akquise neuer Mitarbeiter*innen, Bereichsübergreifender Mitarbeitereinsatz, Einsatz nicht vollausgebildeter Mitarbeiter*innen, Urlaubssperre) **(2.)** - Bereit- und Sicherstellung arbeitsrelevanter Informationen (u.a. Installation von COVID-19-Sprechstunden, Schaffung eines Krisenstabs, Hinterlegung von Informationsmaterialien) **(2.)** - Verringerung der Mehrbelastung durch Verteilung der Verantwortlichkeiten **(2.)**   Technische Lösungen:   - Zur Vermeidung sozialer physischer Kontakte und einem gesicherten Informationsaustausch (u.a. Einsatz von Videotelefonie, E-Learning-Plattformen, Newsletter, telefonische Sprechstunden) **(4.)**   Soziales Miteinander, Kommunikation und Klima:   - Besondere Kommunikation nach innen (u.a. Beruhigung von Mitarbeiter*innen, Mitarbeiterpflege, transparente Kommunikation, dauernde Kommunikationsbereitschaft) **(6.)** - Besondere Kommunikation nach außen (u.a. Kontaktpflege zu pflegenden Angehörigen und Krankenhaussozialdiensten, Netzwerken und Pflege der Verbundenheit, transparente Kommunikation, dauernde Kommunikationsbereitschaft) **(6.)** - Strategien sozialen Miteinanders und guter Führung (u.a. Ausstrahlen von Sicherheit, Fokus auf Ruhe, Kraft und Solidarität, ständige Erreichbarkeit für Mitarbeiter*innen durch Führungskräfte, Einbindung der Mitarbeiter*innen in Entscheidungen/Probleme, Aufklärungsarbeit) **(6.)** - Motivation der Mitarbeiter*innen (u.a. zusätzliches Angebot an Getränken, Verpflegungsgeld, Einkaufsgutscheine, Supervision, Bestärkung bestehender Kompetenzen) **(6.)**   Schulung, Beratung und Aufklärung   - Sicherstellung der Schulung, Beratung und Aufklärung von Mitarbeiter*innen, Pflegebedürftigen und Angehörigen (u.a. transparente Informationspolitik, Flexibilität und Informiertheit, hohe Präsenz der Führung) **(5.)** - Stärkung der Eigenverantwortlichkeit der Mitarbeiter*innen sowie Einhaltung der Hygienevorschriften durch transparente Informationspolitik **(5.)** - Rückgriff auf digitale Medien zur Bereitstellung von Informationen **(5.)**   Mehrarbeit und Zusatzaufwand   - Mehrarbeit (u.a. Überstunden, verstärkter Zusatzaufwand zur Beschaffung von Schutzmitteln, zusätzliche Telefonangebote) **(2.)** - Intensivere Begleitung der Mitarbeiter*innen, Pflegebedürftigen und Angehörige durch Leitungskräfte (insbesondere im Rahmen von Schulung, Beratung und Aufklärung) **(6.)** |
| Hsu YC et al.; April 2020 | Taiwan | Empirische Arbeit | Datenerhebung durch Analyse der offiziellen Webseiten der Krankenhäuser. Falls notwendig, ergänzende Durchführung von Telefonaten | Untersuchung der Veränderungen in der Besucherpolitik für alle 76 Hospizstationen in Taiwan während der COVID-19-Pandemie | - Während der COVID-19-Pandemie änderten fast alle Hospizstationen in Taiwan ihre Besuchspolitik. In 9 Hospizstationen wurden Besuche ganz eingestellt **(3.)** - Mehr als 2/3 der Hospizstationen erlaubten höchstens 2 Besucher*innen pro Patient*in auf einmal und 25,4% erlaubten eine*n Besucher*in **(3.)** - Am weitesten verbreitet waren Besuchsrichtlinien, die eine Stunde der gesamten täglichen Besuchszeit vorsahen **(3.)** - Um Zugang zu Hospizstationen zu erhalten, mussten Ausweisdokumente vorgelegt werden, um die Identität der Besucher*innen zu überprüfen und die Reisegeschichte zu beurteilen **(3.)** |
| Humphreys J et al.; Juli 2020 | USA | Erfahrungsbericht eines stationären Palliativpflegedienstes |  | Beschreibung der raschen und fortlaufenden Implementierung einer telepalliativmedizinischen Beratung für stationäre Palliativpflegeteams und Diskussion der Erfahrungen und Empfehlungen für Programme, die ähnliche Versorgungsmodelle in Erwägung ziehen | - Eine gute Wi-Fi- und/oder Handy-Empfangsqualität und ein klarer Ton sind entscheidend **(4.)** - Die Videokonferenztechnologie sollte idealerweise mit Dolmetscherdiensten integriert werden **(4.)** - Es ist hilfreich, Aufklärungsmaterial zu erstellen oder wiederzuverwenden, um die Patient*innen mit Videokonferenzen vertraut zu machen und ihnen zu zeigen, wie und wann Palliativmediziner*innen sie aus der Ferne kontaktieren können **(5.)** - Viele Patient*innen haben persönliche Smartphones/Geräte, die genutzt werden können, um entfernte Teammitglieder oder Familienangehörige einzubeziehen **(4.)** - Bei einigen Konsultationen ist nach wie vor ein persönliches Gespräch erforderlich, z. B. wenn Patient*innen schwerhörig sind, die Technik nicht nutzen können oder eine körperliche Untersuchung benötigen **(4.)** |
| Janssen DJA et al.; Juni 2020 | Niederlande | Empfehlungen einer internationalen Arbeitsgruppe der European Respiratory Society |  | Erarbeitung von Konsensmpfehlungen für die Palliativversorgung von Patient*innen mit COVID-19 | - Eine ACP (vorausschauende Pflegeplanung) (Erörterung der Ziele und Präferenzen für die künftige medizinische Behandlung und Pflege) sollte von Klinikern routinemäßig mit Patient*innen und ihren Angehörigen bei der Diagnose einer schweren COVID-19 durchgeführt oder überprüft werden. Frühzeitige ACP-Gespräche zum Zeitpunkt des Krankenhausaufenthalts werden empfohlen, um unerwünschte und belastende lebenserhaltende Behandlungen zu vermeiden **(8.)** - ACP sollte vor der Entlassung von genesenen COVID-19-Patient*innen aus dem Krankenhaus neu bewertet werden **(8.)** - Das Personal, das sich um Patient*innen mit schwerem COVID-19 kümmert, sollte in der Optimierung der Kommunikation zwischen Arzt und Patient*in geschult werden, während es persönliche Schutzausrüstung trägt **(5.)** - Das Personal, das sich um Patient*innen mit schwerem COVID-19 kümmert, sollte in der Online-Kommunikation zwischen Arzt und Familie geschult werden (unter Verwendung von Telefon- oder Videokonferenzen) **(5.)** - Die Kommunikation und die Übermittlung schwieriger Nachrichten aus der Ferne erfordert andere kommunikative Fähigkeiten, in denen medizinisches Fachpersonal in der Regel nicht geschult ist **(4.)** - Angehörige von Gesundheitsberufen, die spirituelle Betreuung anbieten (z. B. Seelsorger*innen), sollten Teil des Behandlungsteams von Patient*innen mit schweren COVID-19 mit anhaltenden Symptomen und Bedenken trotz optimaler Krankheitsbehandlung sein (unabhängig von der Umgebung, also im Krankenhaus, in der Gemeinde oder in Langzeitpflegeeinrichtungen) **(6.)** - Fachkräfte des Gesundheitswesens, die psychosoziale Betreuung anbieten (wie Psycholog*innen und Sozialarbeiter*innen), sollten Teil des Behandlungsteams von Patient*innen mit schwerer COVID-19 mit anhaltenden Symptomen und Sorgen trotz optimaler Krankheitsbehandlung sein (unabhängig von der Umgebung, also im Krankenhaus, in der Gemeinde oder in Langzeitpflegeeinrichtungen) **(6.)** - Familienangehörige/Lieblinge sollten eingeladen und unterstützt werden (z. B. durch Bereitstellung von PSA, falls angezeigt), den*die sterbende*n Patient*in mit COVID-19 persönlich zu besuchen **(3.)** - Familienmitglieder/Angehörige von verstorbenen Patient*innen mit COVID-19 sollten Trauerbegleitung durch medizinisches Fachpersonal erhalten, das in Palliativmedizin oder Trauerbegleitung ausgebildet ist **(6.); (10.)** - Dem Personal, das Patient*innen mit schwerem COVID-19 betreut, sollte psychologische Unterstützung angeboten werden, um ihre Erfahrungen zu bewältigen **(6.)** |
| Kates J et al.;  Oktober 2020 | USA | Empirische Arbeit | Querschnittsbefragung von 36 Hospiz- und Palliativpflegekräften | Verständnis der Auswirkungen der COVID-19-Pandemie auf die Hospiz- und Palliativmitarbeiter*innen und die Leistungserbringung | - Die Einrichtungen reagierten auf die Pandemie mit einem verstärkten Einsatz von Telemedizin (88 %), mit dem Einsatz von Technologie zur Besprechung von Versorgungszielen (81 %), mit Routinebesuchen bei Patient*innen (75 %) und mit Familienbesprechungen (69 %) **(4.)** - Als Reaktion auf die Pandemie gaben die Befragten an, dass die Agentur den Mitarbeiter*innen folgende Dienste zur Verfügung stellte: Wellness-Aktivitäten (56 %), individuelle Beratung (53 %), spirituelle Unterstützung (44%), und Selbsthilfegruppen (42%) **(6.)** - Einige Einrichtungen ließen nur Videobesuche zu. Darüber hinaus beschränkten die Einrichtungen, die Besuche zuließen, die Zahl der Besucher*innen **(3.)** - Die Familien von COVID-19-positiven Patient*innen können diese nur durch ein Fenster sehen. Alle Patient*innen, die COVID-19-positiv sind und in den unabhängigen Bereichen leben, sind isoliert, ihre Türen sind mit Plastik abgedichtet und sie sind allein **(3.); (2.)** |
| Kluge S et al.; Mai 2021 | Deutschland | Empfehlungen von Fachgesellschaften |  | Ausgabe von Empfehlungen zur intensivmedizinischen Therapie von Patient*innen mit COVID-19 | - Unterbringung von COVID-19-Patient*innen vorzugsweise einzeln in einem Isolationszimmer, idealerweise mit Schleuse/Vorraum **(2.)** - Im Falle einer ausgeprägten Pandemie sollte eine Kohorten-Isolation angestrebt werden **(2.)** - Strikte räumliche Trennung von SARS-CoV-2-Infizierten und anderen Patient*innen, wenn möglich, 3 räumlich und personell voneinander getrennte Bereiche: **(2.)** - COVID-19-Bereich (alle Patient*innen SARS-CoV-2 positiv) - Verdachtsfall-Bereich - Nicht-COVID-Bereich (alle Patient*innen SARS-CoV-2-negativ und asymptomatisch) - Raumlufttechnische Anlagen nicht abstellen, zusätzliche regelmäßige Fensterlüftung. Luftzirkulation zwischen verschiedenen Räumen sollte vermieden werden **(1.)** - Zugang zu COVID-positiven-Patient*innen nur durch Personal, das für die Versorgung von COVID-19-Patient*innen geschult ist und von der Versorgung anderer Patient*innen freigestellt ist **(2.); (5.)** - Es sollte möglichst immer in festen Teams gearbeitet werden, damit im Falle einer neu aufgetreten Infektion beim Personal möglichst wenige Kontaktpersonen unter dem Personal vorhanden sind **(2.)** - Zahl der Personen, die das Zimmer betreten, ist auf ein Minimum zu reduzieren **(2.)** - Besuche von Angehörigen sollten auf ein Minimum beschränkt und zeitlich beschränkt sein. Stattdessen sollte der Einsatz von technischen Kommunikationsmöglichkeiten (z.B. Videotelefonie via Whatsapp, MS-Teams, Skype) angeboten werden **(3.); (4.)** - Bei der Betreuung der Patient*innen ist unbedingt auf eine konsequente Umsetzung der Basishygiene (einschließlich Händehygiene) sowie auf die korrekte Verwendung der persönlichen Schutzausrüstung (PSA) zu achten. Laut Empfehlungen des RKI besteht PSA aus Schutzkittel, Einweghandschuhen, dicht anliegender Atemschutzmaske (FFP2 bzw. FFP3) und Schutzbrille **(1.)** - Wichtig ist korrekte Verwendung der PSA, dies beinhaltet das kontrollierte Anlegen (insbesondere Dichtsitz der Maske) und das korrekte Ablegen (mit mehrfachen Händedesinfektionen). Mitarbeiter*innen sollten diesbezüglich geschult sein **(1.); (5.)** |
| Lazzarin P et al.; Juni 2020 | Italien | Erfahrungsbericht einer Einrichtung für pädiatrische Palliativmedizin |  | Beschreibung der Bewältigungsstrategien einer pädiatrischen Palliativpflegeeinheit im Umgang mit dem COVID-19-Ausbruch | - Um die Bedürfnisse der Patient*innen und ihrer Familien sowie um die Sicherheit des Personals kümmern **(6.)** - Mit persönlicher Schutzausrüstung (PPE) eindecken **(1.)** - Das Personal für die Behandlung von COVID-19 schulen **(5.)** - Änderungen des Behandlungsstandards wurden in 2 aufeinander folgenden Phasen durchgeführt: - In der ersten Phase wurden nach Rücksprache mit Expert*innen für Infektionskrankheiten die Strategien für den Umgang mit dem Notfall festgelegt. Es wurde PPE-Schulungsmaterial beschafft und dem gesamten Personal zur Verfügung gestellt. Weiterhin wurde der Infektionsstatus aller Gesundheitsdienstleister*innen bewertet und es wurde Aufklärungsmaterial zu COVID-19 für Patient*innen und ihre Familien vorbereitet. Patient*innen und Familien wurden mithilfe von Telemedizin (Audio- und Videokonferenzen mit speziellen mobilen Anwendungen oder internetbasierten Tools) überwacht und geschult. Während der ersten Phase war das Kinderkrankenhaus 7 Tage lang geschlossen **(5.); (1.); (4.)** - In der zweiten Phase wurde der Zugang zum Kinderkrankenhaus gestattet, wenn die Patient*innen eines von folgenden Kriterien erfüllten: Notwendigkeit der Pflege am Lebensende; Symptome wie starke Schmerzen, die zuhause nicht beherrschbar waren; Verschlechterung des klinischen Zustands. Die Patient*innen und ihre Familien wurden auf SARS-CoV-2 getestet, bevor sie in die Kinderklinik eingewiesen wurden. Wenn die Tests negativ waren, konnte das Kind ins Krankenhaus eingeliefert werden. Patient*innen, die positiv auf COVID-19 getestet wurden, wurden auf eine spezielle Station des Krankenhauses überwiesen und von den Mitarbeiter*innen betreut **(1.); (2.)** |
| Luckett T et al.; März 2021 | Australien | Empirische Arbeit | Online-Querschnittsumfrage | Untersuchung der Reaktion der australischen spezialisierten Palliativversorgung auf COVID-19 und seine Folgen, um Informationen für die Pandemiepraxis und -politik zu gewinnen | Personal:   - Ausrüstung des Personals mit PSA und Aufklärung über die ordnungsgemäße Verwendung von PSA **(1.); (5.)** - Aufteilung des Personals in getrennte Teams und Minimierung der persönlichen Treffen **(2.); (1.)** - Beendigung der Freiwilligentätigkeit und Umgruppierung gefährdeter Mitarbeiter*innen zu Arbeiten mit geringerem Risiko, z.B. Zuteilung älterer und/oder kranker Mitarbeiter*innen zu Aufgaben, die es ihnen ermöglichen, von zu Hause aus zu arbeiten **(2.)**   Räume:   - Umgestaltung der Büroräume, um eine räumliche Distanzierung zwischen den Mitarbeitenden zu ermöglichen **(2.)**   Systeme:   - COVID-19-Risikobewertung, Screening und Tests **(1.)** - Telefonischen Triage für persönlichen Kontakt vs. Telemedizin **(4.)** - Nutzung der Telemedizin: Schulung und laufende IT-Unterstützung für Mitarbeiter*innen und Patient*innen; Bereitstellung von Geräten (z.B. Tablets) für die Patient*innen **(4.); (5.)** - Entwicklung von Protokollen für die Betreuung von COVID-19-positiven Patient*innen **(1.)** - Entwicklung eines Dokuments mit Fragen und Antworten zu COVID-19 für Patient*innen **(5.)** - Keine Besucher*innen für Patient*innen, die auf Ergebnisse des COVID-19-Tests warten **(3.)** - Maximal zwei Besucher*innen gleichzeitig **(3.)** |
| Maaskant JM; Dezember 2020 | Niederlande | Empirische Arbeit | Qualitative Studie: retrospektive Durchsicht von Patientenakten und Durchführung halbstrukturierter Fokusgruppeninterviews | - Untersuchung, wie die Einbeziehung der Familienmitglieder in die Patientenpflege bei einer restriktiven Besuchspolitik erfolgt ist und Erforschung der Erfahrungen des Pflegepersonals mit der Einbeziehung der Familien während des COVID-19-Ausbruchs - Formulierung von Empfehlungen für die Einbeziehung der Familie | - Kontakt zwischen Familie und Personal hauptsächlich über Video- oder Telefonanrufe **(4.)** - Betreuungsteams setzten sich proaktiv mindestens einmal täglich mit der Familie in Verbindung, um sie über den aktuellen Stand des Patienten zu informieren **(4.)** - Bei Patient*innen mit Sprachbarrieren wurden entweder Familienangehörige gebeten zu übersetzen oder Kolleg*innen, die die Sprache beherrschen. Die Inanspruchnahme von professionellen Übersetzungsdiensten wurde nur selten erwähnt **(4.)** - Eine wichtige Voraussetzung für den Kontakt zwischen Patient*innen, ihren Familien und/oder medizinischem Fachpersonal war die Verfügbarkeit von (funktionierenden) Telefonen und Tablets und die Fähigkeit, diese zu benutzen. Auf allen Stationen wurden diese Geräte verteilt, aber die Krankenschwestern waren sich der Verfügbarkeit nicht immer bewusst **(4.)** - Besuchsbeschränkungen wurden in Situationen am Lebensende gelockert **(3.)**   Empfehlungen zur Beseitigung von Kommunikationshindernissen durch Telefon- und Videoanrufe:   - Kontaktaufnahme mit der Familie so bald wie möglich nach der Krankenhausaufnahme, um einen Kommunikationsplan zu erstellen. Nach Möglichkeit Einbezug des*r Patient*in **(4.)** - Festlegung des*r (gesetzlichen) Sprecher*in der Familie und Klärung, wie diese Person am besten erreicht werden kann **(4.)** - Organisation eines mindestens einmal täglichen Videotelefonats (oder Telefonkontakt) mit dem*r Sprecher*in der Familie zu einem geplanten Zeitpunkt **(4.)** - Bereitstellung von Telefonen und Tablets für Patient*innen und Angehörige der Gesundheitsberufe, um Videogespräche rund um die Uhr zu ermöglichen. Sicherstellung, dass alle Angehörigen der Gesundheitsberufe über die Verfügbarkeit der Geräte informiert sind **(4.)** - Ermutigung der Familie, Geräte (einschließlich Ladegeräte) zu organisieren, um Videogespräche zu ermöglichen **(4.)** - Bereitstellung von Patientenanweisungen für Videogespräche für verschiedene Sprachniveaus und Sprachen **(5.)** - Organisation von Schulungen für Angehörige der Gesundheitsberufe zur Nutzung der Geräte und zur Unterstützung von Patient*innen und Angehörigen bei der Nutzung der Geräte **(5.)** - Wenn die medizinische Fachkraft am Bett die Kommunikation mit der Familie nicht gewährleisten kann, Übertragung dieser Aufgabe an engagierte Pflegekräfte **(4.)** - Zeitplan für die Kontaktaufnahme mit der Familie durch das Pflegepersonal, das den*die Patient*in betreut, organisieren **(4.)** - Sicherstellen, dass die Gesundheitsfachkraft, die für den Kontakt mit der Familie verantwortlich ist, über alle erforderlichen Informationen verfügt: medizinische, pflegerische und paramedizinische Aspekte der Pflege **(4.); (5.)** - Sicherstellen, dass alle Informationen (klinischer Zustand des*r Patient*in, Situation der Familie und der Inhalt der Kommunikation) dokumentiert werden und allen beteiligten medizinischen Fachkräften zur Verfügung stehen **(4.); (5.)** - Tägliche Beschreibung und Dokumentation der Kommunikation mit der Familie in der Patientenakte **(4.)** - Anweisungen geben, wie Videogespräche am besten geführt werden können, z. B. Aufstellung einer Tagesordnung, Beschränkung des Gesprächs auf die wichtigsten Themen, Ermutigung zum Erzählen von Geschichten **(5.)** - Erwägung der Verwendung eines Kommunikationsrahmens, z.B. CALMER (Check in, Ask, Lay out issues, Motivate, Emotions, Record) **(4.)** - Videoanruf nach Möglichkeit von einem Ort aus durchführen, an dem das Tragen einer Maske nicht erforderlich ist **(4.)** - Wenn Schutzmaterial erforderlich ist, Verwendung von Bildern und Namensschildern, damit die Familie eine Vorstellung davon bekommt, mit wem sie spricht **(4.)** - Inanspruchnahme von Übersetzungsdiensten **(4.)** - Zurückhaltend sein, wenn es darum geht, Familienmitglieder als offizielle Übersetzer einzusetzen **(4.)** - Videoanruf außerhalb der Sichtweite des*r Patient*in beginnen und Familie auf die Situation des*r Patient*in vorbereiten **(4.)** - Emotionalen Zustand der Familie während des Gesprächs überprüfen **(4.); (6.)** - Familienmitglieder darum bitten, das Leben des*r Patient*in, Lebensereignisse und wichtige Personen zu beschreiben **(4.); (6.)** - Informationen über das Lieblingsessen, die Lieblingsmusik und die Fernsehvorlieben des*r Patient*in einholen **(4.); (6.)** - Videoanrufe oder Bilder verwenden, um der Familie die Umgebung des*r Patient*in zu zeigen **(4.)** - Erkundigung nach den Wünschen der Familie und versuchen, sie so weit wie möglich in die Pflege einzubinden **(4.); (6.)** - Ermutigung der Familie, persönliche Gegenstände des*r Patient*in mit ins Krankenhaus zu bringen: Kinderzeichnungen, religiöse Gegenstände, Bilder **(6.)** - Patient*innen helfen und Familie ermutigen, sich gegenseitig Audio-, Video- oder schriftliche Nachrichten zu schicken **(4.); (6.)** - Unterstützung der Familie dabei, religiöse Momente zu teilen, z. B. gemeinsame Gebete **(6.)** - Beachtung kultureller Rituale **(6.)** - Erforschung der Wünsche der Familie, wenn Patient*in im Endstadium ist **(6.)** - Erleichterung der Wünsche so weit wie möglich, ohne die Sicherheitsmaßnahmen zu verletzen **(6.)** - Wenn die Familie auf die Station kommen darf, sicherstellen, dass sie Schutzmaßnahmen strikt einhält **(1.)** - Das Wohlergehen der Familie in die Kommunikation einbeziehen und Bedenken besprechen **(4.); (6.)** - Beurteilung der psychologischen Situation der Familie und Diskussion über Stress und Angst als Teil der Kommunikation. Bei Bedarf Unterstützung organisieren z. B. durch ein Familienunterstützungsteam **(4.); (6.)** - Organisation täglicher Momente der Reflexion, Intervision oder Supervision **(6.)** - Pflegekräfte ermutigen, über ihre Erfahrungen des Tages zu sprechen **(6.)** - Dafür sorgen dass sich die Pflegekräfte unterstützt fühlen, und Bedarf professionelle Hilfe oder Unterstützung durch Gleichaltrige organisieren **(6.)** |
| Mitchell S et al.; Juli 2020 | Vereinigtes Königreich | Übersichtsarbeit |  | Synthese vorhandener Evidenz hinsichtlich der Bereitstellung von Palliativpflege durch primäre Gesundheitsdienste bei Epidemien und Pandemien | - Die gemeindenahe Palliativversorgung wurde als Möglichkeit zur Verbesserung der Lebensqualität und zur Verringerung der Isolation vorgeschlagen **(6.)** - Bei mangelnden Personalressourcen: Beschäftigung von Gemeindegesundheitshelfer*innen und Bereitschaft einzelner Allgemeinärzt*innen, während Pandemien länger zu arbeiten und persönlich zusätzliche Kapazitäten bereitzustellen **(2.)** - Notwendigkeit persönlicher Schutzausrüstung, Distanzierung, regelmäßiger Reinigung der Sprechzimmer, Zugang zu Geräten und rechtzeitige diagnostische Tests, einschließlich Bluttests und Röntgenaufnahmen **(1.)** |
| Morris SE et al; August 2020 | USA | Übersichtsarbeit |  | Darstellung von Vorschlägen, wie Instrumente der Palliativpflege und psychologische Strategien in die Trauerbegleitung für Familien während der COVID-19-Pandemie integriert werden können | Betreuung der Familien vor dem Tod des*r Patient*innen   - Erleichterung von Gesprächen mit dem*r Patient*in und seiner Familie oder mit dem Anbieter, dem* Patient*in und seiner Familie. Bei Bedarf sollten virtuelle Plattformen genutzt werden **(4.)** - Beauftragung eines*r Kliniker*in oder eines anderen Teammitglieds, sich regelmäßig bei der Familie zu melden. Dies bietet Orientierung und Beruhigung und hilft, Angstgefühle abzubauen **(4.)** - Untersuchung der Familienmitglieder auf Stress und Risikofaktoren für einen schlechten Verlauf des Trauerfalls und Angebot von Unterstützung. Dies hilft, eine schwierige Trauerreaktion zu mildern **(6.)** - Versorgung der Familienmitglieder mit aktuellen Informationen, insbesondere in der Endphase des Lebens. Dies hilft dabei, die Erwartungen mit der Realität in Einklang zu bringen und sich auf den Tod des geliebten Menschen vorzubereiten **(6.)** - Wenn Familie zum Zeitpunkt des Todes nicht anwesend ist, sollte der behandelnde Arzt sie sofort anrufen, um sie zu informieren, Fragen zu beantworten und Beileid zu bekunden **(4.); (10.)** - In der Krankenakte nach Hinweisen auf den*die Patient*in vor seiner Erkrankung (Beruf, Familie, Hobbys) suchen und in Gesprächen mit Familie darauf beziehen. Dies fördert die Verbindung und personalisiert die Pflege **(4.); (6.)** - Familien um Fotos bitten, damit die Teams sehen können, wer sie waren, bevor sie krank wurden. Dies fördert die Verbindung und personalisiert die Pflege **(6.)** - Angehörige fragen, ob der*die Patient*in eine Lieblingsmusik hat, und Abspielen der Musik in seinem Krankenzimmer. Dies hilft der Familie, sich in die Pflege des geliebten Menschen einbezogen zu fühlen **(6.)** - Anbringen eines Kennenlern-Posters an der Tür des*r Patient*in, das von einem*r Mitarbeiter*in mit einem Familienmitglied am Telefon erstellt wurde. Dies fördert die Verbindung und personalisiert die Pflege **(6.)** - Teamfoto zusammen mit dem Kennenlern-Poster machen, das an die Familie geschickt wird. Dies fördert die Verbundenheit und personalisiert die Pflege und kann ein wichtiges Erinnerungsstück während der Trauerphase im Rahmen der fortlaufenden Beziehungen sein **(6.)** - Foto des*r Patient*in machen und Familien bereitstellen. Dies hilft dem Familienmitglied, sich verbunden zu fühlen, und kann ein wichtiges Erinnerungsstück während des Trauerfalls im Rahmen der fortbestehenden Bindungen sein **(6.)** - Familien vorschlagen, eine Audioaufnahme zu machen, die das Personal dem*r Patient*in vorspielen kann, um ihm die Dinge zu sagen, die sie ihm persönlich sagen würden. Dies hilft, Schuldgefühle oder Bedauern im Trauerfall zu lindern, insbesondere wenn sie zum Zeitpunkt des Todes nicht anwesend sein konnten **(6.)** - Je nach Infektionsstatus Handabdrücke des*r Patient*in anfertigen. Dies hilft den Hinterbliebenen die Verbindung mit dem geliebten Menschen nach dessen Tod aufrechtzuerhalten **(9.); (10.)** - Herzbild der letzten Tage des*r Patient*in anfertigen, um es der Familie zukommen zu lasse. Dies hilft den Hinterbliebenen die Verbindung mit dem geliebten Menschen nach dessen Tod aufrechtzuerhalten **(9.); (10.)**   Betreuung der Familien nach dem Tod des*r Patient*in   - Auf Gefühle reagieren: „Können Sie mir mehr darüber erzählen?“. Dies hilft den Hinterbliebenen, sich unterstützt zu fühlen, und ermöglicht es ihnen, Informationen zu verarbeiten, sobald die Emotionen anerkannt werden **(10.)** - Auswirkungen der Pandemie anerkennen: „Dies sind beispiellose Zeiten, die Pandemie hat uns alle überrascht“. Dies hilft, das Problem zu externalisieren und realistische Erwartungen in Bezug auf soziale Distanzierung und andere Einschränkungen zu setzen **(10.)** - Traueranruf tätigen. Dies hilft den Hinterbliebenen zu wissen, dass man sich an den*die Patient*in und seine Familie erinnert. Der Traueranruf sollte idealerweise in der ersten Woche nach dem Tod des*r Patient*in stattfinden **(10.)** - Team-Sympathiekarte verschicken. Dies hilft den Hinterbliebenen zu wissen, dass man sich an den*die Patient*in und seine Familie erinnert. Es sollte erwägt werden, dem Team ein Foto des Kennenlern-Posters mitzugeben **(10.)** - Bereitstellung von psychoedukativen Informationen über Trauer. Dies hilft den Hinterbliebenen, einen Plan zu erstellen, was sie erwarten können (z. B. dass die Trauer in Wellen verläuft) **(10.)** - Überweisung zur Trauerbegleitung **(10.)** - Darstellung von Strategien, die den Hinterbliebenen helfen, einschließlich der Anpassung von Ritualen. Dies bietet Struktur und Unterstützung während des Trauerfalls **(10.)** - Ermutigung der Hinterbliebenen, einer Routine zu folgen, auf ihre Selbstfürsorge zu achten und soziale Kontakte mithilfe von Technologien zu pflegen **(10.)** - Familie vorschlagen, eine virtuelle Lebensfeier mit Familie und Freunden zu veranstalten, um in Erinnerungen zu schwelgen, oder dem geliebten Menschen einen Brief zu schreiben, in dem sie ihm sagen, was sie ihm gerne gesagt hätten, vor allem, wenn sie nicht in der Lage waren, sich angemessen zu verabschieden **(10.)** - In Erwägung ziehen, eine Gedenkveranstaltung zu planen **(10.)** - Nicht hilfreiches Denken hinterfragen, ohne die Emotion zu verwerfen. Dies hilft bei der Umstrukturierung nicht hilfreicher Gedanken **(10.)** - Hinterbliebene ermutigen, ihre Gedanken und Gefühle auszudrücken und die Gedanken zu identifizieren und zu hinterfragen, die zu Schuldgefühlen, Schuldzuweisungen oder Wut führen. Eine nützliche Frage, die dabei hilft, die Perspektive zu wechseln, lautet: „Was würden Sie einem Freund in der gleichen Situation sagen?“ **(10.)** - Selbsthilfegruppen vorschlagen. Diese helfen, soziale Kontakte zu knüpfen und den Trauerprozess zu normalisieren **(10.)** - Erwägen, sich zu einem späteren Zeitpunkt mit den trauernden Familien zu treffen, um Fragen zu beantworten, oder einen Gedenkgottesdienst des Teams zu veranstalten, bei dem die Familien zusammenkommen können, um die Kliniker kennenzulernen, die sich um ihre Angehörigen gekümmert haben **(10.)** |
| Münch U et al.; Juni 2020 | Deutschland | Empfehlungen von Fachgesellschaften |  | Ausgabe von Empfehlungen zur psychosozialen und spirituellen Unterstützung von belasteten, schwerstkranken, sterbenden und trauernden Menschen in Zeiten von COVID-19 | Empfehlungen zur psychosozialen und spirituellen Begleitung:   - Patient*innen bedürfen trotz der Isolation ein Gefühl der sozialen Verbundenheit mit ihren Zugehörigen und anderen Personen **(6.)** - Patient*innen benötigen eine individuelle und patientenzentrierte Beratung zu Therapiewünschen und zur Unterstützung bei der Entscheidungsfindung in Bezug auf mögliche Behandlungsmaßnahmen **(8.)** - Patient*innen bedürfen der Unterstützung **(6.)** - beim Umgang mit der Isolation (die möglicherweise. als traumatisch erlebt wird oder alte Traumata reaktiviert) - mit der Möglichkeit, nicht direkt Abschied nehmen zu können - mit der Begrenztheit des eigenen Lebens und der Konfrontation mit der eigenen Sterblichkeit - mit durch die Situation bedingt möglichen Reaktionen wie Angst, Demoralisierung, Stress und Depressivität - bei spirituellen und existenziellen Nöten - Patient*innen bedürfen der Beratung **(6.); (8.)** - zu ihrer sozialen Situation und Unterstützungsmöglichkeiten - zur Patientenverfügung, Vorsorgevollmacht und Testament (falls noch nicht vorhanden oder nicht für die neue Situation ausreichend aktuell) - Es ist sicherzustellen, dass trotz der Einschränkungen durch COVID-19 psychosoziale und spirituelle Unterstützung und Hilfen angeboten werden **(6.)** - Bei Besuchsverboten prüfen, ob für Palliativpatient*innen Ausnahmeregelungen eingeführt werden können **(3.)** - Alternative Möglichkeiten der Kommunikation für isolierte Patient*innen mit COVID-19 und Patient*innen, die unter Besuchsverboten leiden, überprüfen (Telefon, Videotelefonie oder soziale Medien) **(4.)** - Smartphones oder Tablets mit entsprechenden Apps nutzen (Tablets, deren Tastatur angemessen desinfiziert werden kann) **(4.)** - Auch für Psycholog*innen, Sozialarbeiter*innen, Psychotherapeut*innen, Seelsorger*innen durch Telefon oder Videotelefone Kontakt mit Patient*innen einrichten **(6.)** - (Möglichst datenschutzkonforme) Videotelefonie oder Apps für Smartphones, Tablets oder Notebooks nutzen, wie z.B. Skype, Zoom oder anderen kostenlose Angeboten. Dadurch können Patient*innen und Angehörige sich zum Kaffee verabreden, gemeinsam essen, beten, singen oder Gottesdienst feiern. Sie können Bilder und Lieblingslieder schicken oder Videos miteinander teilen **(4.); (6.)** - Andere Möglichkeiten für Zugehörige Kontakt zu halten: Karten/Briefe, Bilder malen oder den Patient*innen Fotos in Erinnerung an die gemeinsame Zeit senden. Solche Gegenstände können **(6.)** - von Patient*innen immer wieder in die Hand genommen, betrachtet und gelesen werden oder von Mitarbeiter*innen vorgelesen werden **(6.)** - Festlegung eines*r festen Ansprechpartner*in für Angehörige, der regelmäßig einfühlsam über den Zustand des erkrankten Menschen informiert **(4.); (6.)** - In die Begleitung der Zugehörigen können neben Psycholog*innen, Psychoonkolog*innen, Psychotherapeut*innen, Sozialarbeiter*innen und Seelsorger*innen insbesondere ambulante Koordinator*innen oder ehrenamtliche Mitarbeiter*innen der ambulanten Hospizdienste oder anderer Dienste eingebunden werden und per Telefon oder Videotelefonie Informationen und Unterstützung bieten **(6.)** - Evaluieren, ob Möglichkeit besteht, von an COVID-19 Verstorbenen in der Pathologie unter Einhaltung der Schutzmaßnahmen Abschied zu nehmen oder Unterstützung der Zugehörigen dabei, wie sie auch ohne Leichnam Abschied nehmen können, z.B. mit einem Foto des Verstorbenen, durch Schreiben eines Briefes an den Verstorbenen, durch Gespräche über unerfüllte Bedürfnisse oder über das Beste, was man dem verstorbenen Menschen auf den Weg geben möchte **(9.)** - Hilfestellung der trauernden Angehörigen dabei, wie sie zu Angeboten der Trauerberatung (telefonisch oder videogestützt) finden können, wie sie mit ihren Kindern so kommunizieren, dass Krankheit und Verlust ohne direkte Abschiedsmöglichkeit auch von diesen möglichst ohne ungünstige Nebenwirkungen verarbeitet werden können, wie sie diejenigen, die von Verlust am stärksten betroffen sind, sensibel und einfühlsam auch in diesen Zeiten unterstützen können **(9.); (10.)** - Es wäre hilfreich, wenn sich die Behandlungsteams nach dem Versterben der Patient*in um die Zugehörigen sorgten. Liegt das Einverständnis der engeren Bezugspersonen/der Klinik für eine Nachsorge vor, dann könnten Mitarbeitende aus dem Team spezifisch damit beauftragt werden, diese Zugehörigen spätestens 2 bis 4 Wochen nach dem Versterben der Patient*in zu kontaktieren. Sie könnten ihnen in dem Gespräch z. B. einfühlsam zuhören oder Informationen vermitteln, wo sie Unterstützung für ihre möglichen spezifischen Probleme erhalten können **(10.)** - Sollten Fachkräfte merken, dass Hinterbliebene darunter leiden, dass die Teilnahme an der Bestattung nur wenigen Personen vorbehalten ist, dann könnten sie Betroffene dazu anregen, dies später nach dem Abklingen der Corona-Pandemie nachzuholen, beispielsweise durch eine persönlich gestaltete Gedenkfeier zu Hause oder an einem für die verstorbene Person bedeutungsvollen Ort mit allen Freunden und Nahestehenden **(10.)** - Es können regelmäßige Kontakte via Telefon oder soziale Medien mit Mitarbeiter*innen der ambulanten Hospizdienste, der Seelsorge oder anderer Trauerberatungsstellen hilfreich sein **(10.)** - Trauernde können Erinnerungsstücke (zusammen mit anderen Zugehörigen) real an einem Ort (Erinnerungskiste, Fotobuch) oder auch virtuell sammeln (Bilder, Texte und Geschichten; siehe zum Beispiel gedenkseiten.de) **(10.)**   Entlastung der Mitarbeiter*innen   - Klare Kommunikationsstrukturen vorhalten: Informations- und Kommunikationskonzept, nach welchem in regelmäßigen Abständen konkrete Informationen zur aktuellen Lage, Regelungen zu Abläufen, Zuständigkeiten, Maßnahmen zur Bewältigung der Lage und mögliche Szenarien, deren Handhabung und den Implikationen für die Behandlungsteams gegeben werden **(5.)** - Kurzfristige Schulung der Mitarbeiter*innen in hilfreicher Kommunikation für besonders herausfordernde Situationen **(5.)** - Schutzkleidung, Rückzugsräume, verlässliche Pausen und zuverlässig gute Verpflegung für Mitarbeiter*innen **(6.)**   Kommunikation   - Videogestützte Kommunikation mit Laptop, Tablet oder Smartphone **(4.)** - Telefonische Kommunikation **(4.)** - Direkte Kommunikation in Schutzkleidung **(4.)**   Einbindung von hospizlich- palliativ qualifizierten Mitarbeiter*innen der psychosozialen und spirituellen Versorgung   - Es wird der kurzfristige Aufbau einer klinischen psychosozialen Notfallversorgung im Rahmen der COVID-19-Pandemie empfohlen **(6.)** - Sowohl für die Versorgung der Patient*innen mit COVID-19, die nicht intensivmedizinisch versorgt und beatmet werden, wie auch für Palliativpatient*innen ohne COVID-19 und kritisch kranke Patient*innen auf Intensivstationen bedarf es der Einbeziehung von Expert*innen für die psychologische, soziale und spirituelle Versorgung dieser besonders vulnerablen Personengruppen und ihrer Zugehörigen **(6.)** - In der Hospiz- und Palliativversorgung qualifizierte Fachkräfte verfügen über Wissen und Kompetenzen im Umgang mit existenziellen Situationen wie Sterben, Tod und Trauer **(6.)** - Vor allem bei komplexer und intensiv ausgeprägter Problematik ist je nach Schwerpunkt des Unterstützungsbedarfs das Hinzuziehen von Sozialarbeiter*innen, Psycholog*innen, Psychoonkolog*innen, Psychotherapeut*innen, Seelsorger*innen, Hospizkoordinator*innen und Trauerberater*innen/-begleiter*innen angezeigt. Sie verfügen über Möglichkeiten und Techniken, **(6.)** - dem Gegenüber würdewahrend mit Präsenz, Mitgefühl und Achtsamkeit zu begegnen **(6.)** - Emotionen auszuhalten, aufzufangen und in einen möglichst erträglichen Bereich zu führen **(6.)** - Ressourcen zu aktivieren, eigene Kraftquellen zu finden und zugänglich zu machen **(6.)** - Menschen zu unterstützen, einen eigenen Umgang mit der Situation zu finden **(6.)** - zwischen verschiedenen Gruppen (z. B. verschiedenen Berufsgruppen untereinander bzw. Zugehörigen und Mitarbeiter*innen) zu vermitteln und Verständnis füreinander auch in schwierigen Zeiten zu finden **(6.)** - Menschen dabei zu unterstützen, sich selbstwirksam zu erleben, aber auch Dinge annehmen zu können, die nicht veränderbar sind **(6.)** - andere nicht hospizlich-palliativ qualifizierte und/oder erfahrene Kolleg*innen zu schulen, damit diese ebenfalls in diesen Bereichen eingesetzt werden können **(6.)** - interkulturelle Kompetenzen anzuwenden **(6.)** - Allen Mitarbeiter*innen, die an der Behandlung von Patient*innen mit COVID-19 beteiligt sind, sollte zur Selbstfürsorge und Psychohygiene die Möglichkeit von Supervision bzw. Intervision angeboten werden **(6.)** |
| Ong KJ et al.; August 2020 | Singapur | Letter to the Editor |  | Vorstellung der Erfahrungen und Empfehlungen eines Palliativpflege-Beratungsteams in einem Allgemeinkrankenhaus während der COVID-19-Pandemie | Strategien zur Verbesserung der Kommunikation zwischen Patient*innen und Familienangehörigen   - Elektronische Geräte wie Mobiltelefone, elektronische Tablets und Laptops sind nützliche Hilfsmittel für die Kommunikation im Krankenhaus **(4.)** - Krankenhäuser sollten mit einer guten Wi-Fi-Verbindung ausgestattet sein, damit technisch versierte Patient*innen und ihre Familien diese Geräte nutzen können, und einen Vorrat an Ladegeräten und Kabeln bereithalten, falls die Patient*innen sie ausleihen müssen **(4.)** - Das Krankenhauspersonal kann sich freiwillig melden, um weniger versierten Patient*innen und ihren Familien den Umgang mit diesen Geräten zu erleichtern und so die Lebensqualität der Patient*innen zu verbessern **(4.)**   Schulung des medizinischen Personals und der Familien, wie man trotz Maske kommunizieren kann   - Angehörige sehen im Tragen einer Maske ein Hindernis für die Kommunikation mit dem*r sterbenden Patient*in. Außerdem wird ein Mangel an Empathie seitens des maskierten Gesundheitspersonals als negativ empfunden, deshalb sollten andere Kommunikationsmittel genutzt werden, und zwar durch Körpersprache, ausdrucksstarken Augenkontakt, körperliche Berührung mit sauberen Händen (mit Ermahnungen, das eigene Gesicht nicht zu berühren, gefolgt von gründlichem Händewaschen) und tonale Veränderungen des verbalen Ausdrucks **(4.)** - Familienmitglieder von Patient*innen mit verändertem Geisteszustand, wie z.B. demenzerkrankten Patient*innen, können bei ihren Kontakten einen durchsichtigen Gesichtsschutz tragen. Die Möglichkeit, ein vertrautes Gesicht zu sehen, hilft dem*r Patient*in, sich neu zu orientieren und die Kommunikation mit seinen Angehörigen zu verbessern **(4.)**   Komplizierte Trauer   - Angehörige von Patient*innen, die in diesen schwierigen Zeiten im Krankenhaus sterben, neigen zu komplizierter Trauer. Palliativpflegeteams können die Familien durch frühzeitige Gespräche über die Sterbebegleitung unterstützen und sie mit dem Wissen ausstatten, was sie bei einem*r sterbenden Patient*in erwarten können **(8.)** |
| Pearce C et al.; März 2021 | Vereinigtes Königreich, Irland | Empirische Arbeit | Online-Querschnittsbefragung von Gesundheits- und Sozialeinrichtungen | Untersuchung der Erfahrungen und Ansichten von Fachkräften des Gesundheits- und Sozialwesens im Vereinigten Königreich und in Irland in Bezug auf Veränderungen in der Sterbebegleitung während der COVID-19-Pandemie | - Bereitstellung und Einsatz von Telefon, Video und andere Formen der Fernbetreuung für die Trauerbegleitung **(4.)** - Frühere telefonische Kontaktaufnahme mit Familien von Patient*innen, die im Krankenhaus sterben, um eine ausführlichere „Nachbesprechung“ durchzuführen **(4.); (10.)** - Möglichkeit der Mail von Bildern durch Angehörige, die in Patientenzimmer aufgehängt werden **(4.); (6.)** - Versorgung der Hinterbliebenen mit Erinnerungsstücken, wie gestrickte Herzen, das Versenden von Beileidskarten und die Organisation der Rückgabe von Eigentum des Verstorbenen **(10.)** - Ermöglichung regelmäßiger Kommunikation mit den Familien vor und nach einem Todesfall, um sicherzustellen, dass die Familien die Möglichkeit haben, Fragen zu stellen und sich zu beruhigen **(4.); (10.)** - Wenn möglich, Wege für Familien finden, um mit sterbenden geliebten Menschen zusammen zu sein **(6.)** - Die Bedürfnisse der trauernden Familien in die Kommunikation einbeziehen, um diejenigen zu identifizieren und anzusprechen, die weitere Unterstützung benötigen **(4.); (6.)** - Anerkennung der Herausforderungen für das Personal und Förderung kurzer Schulungen für diejenigen, die sich nicht in der Lage fühlen, mit den Bedürfnissen trauernder Familien umzugehen **(5.); (6.)** |
| Pickell Z et al.; Dezember 2020 | USA | Meinungsartikel |  | Darstellung der Einführung virtueller Freiwilligenarbeit während der COVID-19-Pandemie | - Freiwilligenprogramme sollten versuchen, virtuelle Alternativen zu entwickeln **(4.)** - Einführung virtueller Freiwilligenarbeit zur Unterstützung von Patient*innen und Mitarbeiter*innen während der COVID-19-Pandemie **(4.)** - Freiwillige könnten mit dem Health Insurance Portability and Accountability Act (HIPAA) konforme, verschlüsselte Videoplattformen wie Zoom und BlueJeans nutzen **(4.)** - Vor allem Überangebot an Bildschirmen und Technologien macht Krankenhäuser gut gerüstet, um virtuelle Freiwilligenprogramme für Patient*innen unabhängig von ihrem COVID-19-Infektionsstatus umzusetzen **(4.)** - Die virtuelle Freiwilligenarbeit im Krankenhaus kann die physischen Aufgaben, die Freiwillige normalerweise in Krankenhäusern übernehmen, nicht ersetzen, aber sie kann wichtige Patientenkontaktdienste übernehmen, die Freiwillige vor der Pandemie geleistet haben **(4.)** - Die virtuelle Freiwilligenarbeit im Krankenhaus ermöglicht den Patient*innen, wieder die lebenswichtige pädagogische Unterstützung zu erhalten, die sie vor COVID-19 hatten **(4.); (6.)** - Virtuelle Freiwilligenarbeit bietet emotionale Unterstützung und Trost für Patient*innen, die keine Freunde oder Familienangehörigen haben, die sie kontaktieren können, oder die ihre Angehörigen nicht erreichen können, weil sie keinen Zugang zur Technologie haben **(4.); (6.)** - Die virtuelle Freiwilligenarbeit minimiert das Risiko der Virusausbreitung und bietet Patient*innen und Familien wichtige psychosoziale und pädagogische Entwicklungsmöglichkeiten **(4.); (6.)** |
| Powell V, Silveira MJ; Juli 2020 | USA | Empfehlungen von Expert*innen |  | Skizzierung der Herausforderungen, denen sich Palliativkonsiliardienste in der COVID-19-Pandemie stellen müssen und Vorschlag einiger Alternativen | - Begrenzung der Anzahl der Palliativmediziner*innen, die mit COVID-19-Patient*innen direkten Kontakt haben. Es sollte idealerweise nur ein Leistungserbringer das Zimmer betreten **(1.)** - Palliativmediziner*innen sollten alle üblichen Vorsichtsmaßnahmen befolgen und körperliche Berührungen unterlassen, auch bei nicht infizierten Familienmitgliedern der Patient*innen **(1.)** - Wann immer es möglich ist, sollte in Erwägung gezogen werden, Patient*innen oder Familien telefonisch statt persönlich zu kontaktieren **(4.)** - Teammitglieder, die aufgrund ihres Gesundheitszustands einem höheren Risiko ausgesetzt sind (z. B. fortgeschrittenes Alter, Diabetes, Immunsuppression, Schwangerschaft), sollten die Zimmer der COVID-19-Patient*innen nicht betreten **(2.)** - Nutzung von VitalTalk, einem evidenzbasierten Lehrplan für die Kommunikationsausbildung, für die Anleitung von Klinikern zu schwierigen Gesprächen **(5.); (4.)** |
| Reinhard MA et al.; Juli 2021 | Deutschland | Übersichtsarbeit |  | Beschreibung der Entwicklung des Konzeptes Psychosoziale Versorgung COVID 19 am LMU-Klinikum in München | - Aufbau eines effizienten neuen Moduls zur psychosozialen Versorgung von COVID-19-Patient*innen, Angehörigen und Klinikpersonal **(6.)** - Das Konzept wurde von einem interdisziplinären Team bestehend aus Psychiater*innen, Psycholog*innen, Seelsorger*innen, Psychoonkolog*innen und Palliativmediziner*innen mit fünf Bausteinen entwickelt: **(6.)**  1. Patientenhotline #WirSindFürSieDa 2. Psychosoziale Unterstützung stationärer COVID-19-Patient*innen mittels Tablet #GemeinsamGegenDieKrise 3. Psychiatrischer Konsiliardienst 4. Psychosoziale Unterstützung für Angehörige 5. Mitarbeiter*innen-Hotline #HelpTheHelper   Patientenhotline #WirSindFürSieDa   - Die Patient*innen-Hotline wurde für alle Patient*innen des LMU-Klinikums eingerichtet, da auch Nicht-COVID-19-Patient*innen durch die Besuchseinschränkungen erheblich belastet waren **(6.); (4.)** - Die Betreuung der Hotline durch die Klinikseelsorge, die bereits über die etablierte Struktur einer 24/7-Telefonbereitschaft verfügte, ermöglichte eine schnelle und zuverlässige Umsetzung mit 24-stündiger, klinikweiter Erreichbarkeit unter einer zentralen, leicht zu merkenden Durchwahl **(6.); (4.)** - Die hauptamtlichen Seelsorger*innen waren durch ihre Expertise zeitnah in der Lage, den individuellen Versorgungsbedarf zu erheben und wo nötig und angebracht, weitere psychosoziale Anlaufstellen einzuschalten **(6.)** - Auch die Möglichkeit einer längerfristigen telefonischen Begleitung durch den Stamm der erfahrenen ehrenamtlichen Seelsorger*innen wurde vorbereitet **(6.); (4.)**   Psychosoziale Unterstützung von COVID-19-Patient*innen mittels Tablet #GemeinsamGegenDieKrise   - Auf den COVID-19-Stationen des Klinikums wurde für stationäre Patient*innen mittels Videosprechstunde ein spezielles psychotherapeutisches Angebot mit supportiven Kurzinterventionen geschaffen **(6.); (4.)** - Inhaltlich wurde als Gesprächsleitfaden das BELLA-Konzept für psychologische Kriseninterventionen mit einer ressourcenorientieren Grundhaltung herangezogen **(4.)** - Neben dem Beziehungsaufbau, der mittels Videosprechstunde erfahrungsgemäß schneller gelingt als am Telefon, konnte durch Validierung von Emotionen und Ressourcenaktivierung bereits in vielen Fällen eine emotionale Entlastung der Patient*innen erreicht werden **(6.)** - Neben Achtsamkeits- und Imaginationsübungen zur Entspannung wurde der*die Patient*in zu eigenständigen Übungen wie beispielsweise Anti-Grübel-Strategien oder dem „butterfly hug“, einer Technik zur Selbstberuhigung, angeleitet **(6.)** - Termine mit Psycholog*innen und Ärzt*innen der Klinik für Psychiatrie und Psychotherapie, die sich zeitweise selbst im Homeoffice befanden **(6.)** - Klinik erhielt Anfang April 2020 eine Spende von 60 Tabletgeräten, die von Mitarbeiter*innen Münchner Unternehmen zur Verfügung gestellt und auf COVID-19-Stationen verteilt wurden **(2.); (4.)** - Die Tablets konnten auch für die Kommunikation der Patient*innen mit ihren Angehörigen eingesetzt werden und enthielten vorinstallierte Podcasts mit Entspannungsübungen und Informationsmaterial **(4.); (6.)**   Psychiatrischer Konsiliardienst   - Beim Vorliegen von „red flags“ (z. B. Suizidalität, Delir, Notwendigkeit psychopharmakologischer Behandlung) konnte der psychiatrische Konsiliardienst in die Behandlung von COVID-19-Patient*innen einbezogen werden (**6.)** - Bei den strengen Infektionsschutzmaßnahmen erleichterten die Tablets auch hier die interdisziplinäre Kommunikation zwischen Psychiater*innen, Patient*innen und den COVID-19-Behandlungsteams **(4.)**   Psychosoziale Unterstützung für Angehörige   - Das Beratungsangebot für Angehörige von COVID-19-Patient*innen beinhaltete lösungsorientierte, supportive Gespräche **(6.)** - Auch eine Weitervermittlung an interne und externe Hilfsangebote (z. B. Sozialdienst, Seelsorge, Familiensprechstunde, niedergelassene Psychotherapeut*innen) fand nach Bedarf statt **(6.)** - Das Angebot für Angehörige baute auf die bestehenden Strukturen der Psychoonkologie auf und wurde wochentags vom psychologischen Team der Klinik für Anästhesiologie, Klinik und Poliklinik für Orthopädie, Physikalische Medizin und Rehabilitation sowie der Psychoonkologie in Kooperation mit lebensmut e. V. gewährleistet **(2.)** - Außerhalb dieser Zeiten bestand eine notfallmäßige Rufbereitschaft, sodass Angehörige 24/7 versorgt werden konnten **(4.)**   Psychosoziale Hotline für Mitarbeiter*innen #HelpTheHelper   - Ergänzend zu bereits bestehenden Maßnahmen der Gesundheitsförderung (Teambesprechungen, Mitarbeiter*innen-Gespräche, Angebote zur Kinderbetreuung usw.) wurde mit der #Help eHelper-Hotline ein niederschwelliges und anonymes Angebot eingerichtet **(6.)** - Ein nach dem BELLA-Konzept geschultes Team psychologischer und ärztlicher Mitarbeiter*innen war täglich von 8 bis 20 Uhr auf einer zentralen Nummer auch von außerhalb des Klinikums erreichbar **(6.); (4.)** - In wöchentlichen videobasierten Supervisionen wurden die geführten Telefonate strukturiert anhand eines anonymisierten Dokumentationsbogens nachbesprochen und der Versorgungsbedarf evaluiert **(6.)** - Neben der direkt-supportiven Funktion diente die Hotline der Identifikation von Mitarbeiter*innen mit interventionsbedürftigen psychischen Beschwerden **(6.)** - In Zusammenarbeit mit der Ambulanz der Klinik für Psychiatrie und Psychotherapie fand die Vermittlung einer regelmäßigen (evtl. videobasierten) psychotherapeutischen und/oder psychiatrischen Versorgung statt **(6.)** - Als Investitionskosten fielen die Beschaffung von Tablets und Smartphones an, auf denen die kostenlose Videosprechstunde installiert wurde **(4.); (2.)** - Zusätzlich wurde über das Klinische Arbeitsplatzsystem die Möglichkeit eingerichtet, klinikweit Aufträge an das psychosoziale Modul zu stellen **(6.)** - Das Angebot wurde mittels verschiedener Werbemaßnahmen bekannt gemacht (Flyer, Intranet, Informationsvideos, E-Mail, persönliche Vorstellung und technische Unterstützung auf den COVID- 19-Stationen) **(4.)** - Das inhaltliche Konzept wurde in zwei webbasierten Schulungen vermittelt (à 90 min) und eine wöchentliche Supervisionsmöglichkeit angeboten (à 60 min) **(5.)** - Personell wurde das Angebot durch vorhandenes Personal und ehrenamtliche Helfer*innen bereitgestellt, welches aufgrund der mit der Pandemie einhergehenden reduzierten Belegung der Kliniken über zeitliche Kapazitäten verfügte **(2.)** |
| Roser T et al.; März 2020 | Deutschland | Empfehlungen von Expert*innen |  | Empfehlungen für Seelsorger*innen im Rahmen der COVID-19-Pandemie | Zur Begleitung und Betreuung von Patient*innen:   - Seelsorger*innen sollten sich in den behandelnden Teams für die Wahrnehmung der psychosozialen und spirituellen Bedürfnisse und eine entsprechende Betreuung von Patient*innen einsetzen **(6.)** - Bei allen Patient*innen, insbesondere Sterbenden, ist eine Betreuung gemäß ihrer Religionszugehörigkeit und ihren Wünschen und Bedürfnissen zu ermöglichen **(6.)** - Bei sterbenden Patient*innen sollte für die Angehörigen Möglichkeit zum Abschied bestehen **(9.)** - Eventuell können Pflegende stellvertretend einen Segen für die Sterbenden sprechen („Der Herr behüte dich und segne dich“) **(9.)** - Bei verstorbenen Patient*innen sollte eine Aussegnung/Abschniednehmen möglich sein, ggf. durch digitale Medien („Telechaplaincy“) **(9.)** - Wenn eine Abschiednahme im direkten Kontakt nicht möglich ist, sollte Angehörigen ein symbolisches Abschiedsritual in der Klinikkapelle o.A. ermöglicht werden. Zu beachten ist dabei die zulässige Größe von Gruppen **(9.); (1.)** - Es ist hilfreich, wenn die Seelsorge über ihr spezifisches Angebot für Angehörige und Patient*innen auf geeignete Weise aufmerksam macht (z.B. auf Website der Institution) **(6.); (5.)**   Zur Begleitung und Betreuung von Angehörigen:   - Seelsorger*innen sollten sich im Gespräch mit den Stations- und Einrichtungsleitungen als „Anwalt der Familie“ für humane Regelungen der Besuchsmöglichkeiten oder „Ausnahmen von der Regel“ einsetzen und evtl. Angehörige begleiten **(3.)** - Seelsorger*innen sollten aber auch Risiken ansprechen, die ein Besuch für die Familie mit sich bringt und sich die Bedürfnisse des Personals und der Klinik anhören **(1.); (6.)** - Es können digitale Botschaften von den Angehörigen/von den Patient*innen durch die Seelsorger*innen überbracht werden (z.B. Aufnahme von Fotos oder kurzen Grußbotschaften mit Handykamera). Das Handy sollte dabei nicht aus der Hand gegeben werden **(6.); (4.); (1.)** - Wo kein persönliches Abschiednehmen möglich ist, sollten Fotografien der Verstorbenen gemacht und in der Klinik aufbewahrt werden **(9.)** - Dies kann später ein wichtiges Dokument für Trauernde sein, die sich unsicher sind, ob ihr*e Patient*in wirklich gestorben ist **(10.)** - Seelsorger*innen sollten Familien anbieten, in der Klinikkapelle ihrer Beziehung zum*r Patient*in Ausdruck zu geben und in Kontakt mit dem Trost und der Unterstützung des Glaubens zu kommen. Dabei könnte eine Kerze angezündet werden, ein Gebet gesprochen oder in ein Gebetsbuch eingetragen werden **(6.); (10.)**   Zur Begleitung von belasteten Mitarbeitenden:   - Seelsorger*innen und Mitarbeiter*innen der psychosozialen Berufsgruppen können proaktiv auf Mitarbeiter*innen zugehen, sie nach Belastungen fragen und Unterstützung anbieten **(6.)** - Solche Gespräche sind am ehesten in Pausen oder nach Dienstende bzw. am Ende der Belastungszeit angebracht **(6.)** - Einzelgespräche und Angebote wie Achtsamkeitsübungen, Gebete und Gedenken an Verstorbene können unterstützend und stabilisierend wirken **(6.)** |
| Schwartz J et al.; Mai 2021 | Deutschland | Erfahrungsbericht eines Universitätsklinikums |  | Vorstellung der Erfahrungen und Best-Practice-Beispiele aus der Palliativversorgung in einem Universitätsklinikum mit besonderem Fokus auf onkologische Patient*innen | Besuchsregelungen:   - Ausnahmeregelungen sollten mindestens den Besuch und die Begleitung sterbender Patienten*innen ermöglichen **(3.)** - Eine 24h-Begleitung durch ein bis zwei nächste Angehörige sollte auf allen Stationen im Einzelfall umsetzbar sein **(3.)** - Festlegungen sollten Personenzahl und Besuchsdauer umfassen, aber auch z.B. die Begleitungsmöglichkeit minderjähriger oder (hoch)betagter Angehöriger **(3.)** - Einzuhaltende Hygienemaßnahmen und logistische Abläufe, z.B. Anmeldung eines Besuchs an zentraler Besucherkontrollstelle, müssen bekannt sein **(1.)** - Einsatz von Antigenschnelltests, um Besucher*innen, aber auch Personal Sicherheit zu vermitteln **(1.)** - Es sollten Aufklärungsgespräche oder Therapiezielgespräche mit Patient*innen und Angehörigen gemeinsam mit dem Arzt durchgeführt werden **(5.); (8.)** - Es sollte eine möglichst rasche Entlassung der Patient*innen ermöglicht werden, wodurch eine soziale Isolation durch Besuchseinschränkung vermieden wird **(6.)**   Kommunikation   - In der Regel sind über private Smartphones der Patient*innen Apps zur Videotelefonie verfügbar oder können eingerichtet werden **(4.)** - Klinik sollten vor allem in Zeiten einer Pandemie WLAN kostenlos zur Verfügung stellen **(4.)** - Gegebenenfalls kann bei der Bedienung der digitalen Geräte Personal vor Ort unterstützen **(4.)** - Alternativ sollten Kliniken eigene Tablets vorhalten und diesen Patient*innen bereitstellen **(4.)** - Kommunikationstrainings für Mitarbeiter*innen in der anspruchsvollen Überbringung schlechter Nachrichten per Telefon unterstützen **(5.)**   Abschied und Trauer   - Bereitstellung zusätzlicher Abschiedsräume, wo mehrere Angehörige die Gelegenheit haben, sich von Patient*innen zu verabschieden **(9.)** - Umsetzbar sind z. B. mehrere zeitlich versetzte Besuche von Kleingruppen **(9.)** - Häufig sind Abschiedsräume, wenn vorhanden, am Ende eines Stationsflurs lokalisiert, sodass sich Publikumsverkehr vermeiden bzw. reduzieren lässt **(9.)** - Können Angehörige, z. B. bei eigener Erkrankung oder wegen großer Entfernung, nicht zum Abschiednehmen kommen, können durch das Personal Fotos der Verstorbenen gemacht und an die Hinterbliebenen versendet werden **(9.)** - Es sollten möglichst die Mitarbeiter*innen, die den*die Patient*in in den letzten Lebenstagen betreut haben, für Gespräche im Nachgang zur Verfügung stehen, um speziell die Angehörigen aufzufangen, die nicht persönlich Abschied nehmen konnten **(9.)** |
| Selman LE et al.; August 2020 | Vereinigtes Königreich | Übersichtsarbeit |  | Überblick über die Risikofaktoren für Trauer bei COVID-19, Vorstellung evidenzbasierter Empfehlungen für die Unterstützung trauernder Angehöriger und Hinweis auf zusätzliche Ressourcen | Vor dem Tod eines*r Patient*in:   - Frühzeitige Gespräche zur Vorausplanung der Versorgung mit Patient*innen und Familien **(8.)** - Rechtzeitige, proaktive und einfühlsame Information und Kommunikation mit den Angehörigen: Wertschätzung der Aussagen von Familienmitgliedern; Anerkennung der Emotionen von Familienmitgliedern; Anhören ihrer Sorgen; Verstehen, wer der*die Patient*in im aktiven Leben war, indem Fragen gestellt werden; auf Fragen der Familienmitgliedern eingehen **(4.); (6.)** - Es sollte, wenn möglich, für jede*n Patient*in eine bestimmte Kontaktperson benannt werden, um die Kontinuität der Pflege und die rechtzeitige Kommunikation mit den Familien vor und nach dem Tod zu gewährleisten **(4.)** - Einem Familienmitglied, wenn möglich, Besuch eines*r sich verschlechternden Patient*in ermöglichen **(3.)** - Erleichterung virtueller Kommunikation mit Smartphones, Tablet-Computern und anderen Technologien **(4.)** - Es sollte um Spenden für die Beschaffung von Tablets, Smartphones und Ladegeräten gebeten werden **(4.); (2.)** - Sicherstellen, dass Patient*innen und Familien Zugang zu emotionaler, psychologischer und spiritueller Unterstützung haben, einschließlich des Zugangs zu Seelsorger*innen **(6.)**   Nach dem Tod des*r Patient*in   - Einige Familien möchten vielleicht Erinnerungsstücke oder Andenken (z. B. Haarlocken, Handabdrücke usw.) **(10.)** - Solche Andenken sollten in einem versiegelten Beutel aufbewahrt und frühestens nach sieben Tagen geöffnet werden **(10.)** - Sicherstellen, dass ein*e beteiligte*r Mediziner*in nach dem Tod zur Verfügung steht, um mit den Familienmitgliedern zu sprechen und ihnen zuzuhören, die Geschehnisse zu besprechen und Fragen per Telefon zu beantworten **(10.)** - Angehörige identifizieren, bei denen ein besonderes Risiko für einen schlechten Verlauf des Trauerfalls besteht (z. B. aufgrund sozialer Isolation), damit sie besser betreut und unterstützt werden **(10.)** - Fachkräfte aus anderen Fachbereichen des Krankenhauses hinzuziehen, deren Arbeitsbelastung während der Pandemie möglicherweise abgenommen hat, um die psychosoziale Betreuung der trauernden Familien zu unterstützen **(2.); (6.)** - Erstellung einer COVID-19-Trauerbroschüre, die Angehörige auf lokale Trauerhilfe per E-Mail, Telefon, mobile Apps, Webforen, Webchats und virtuelle Peer-Unterstützung hinweist und ihnen zeigt, wo sie glaubensspezifische Beratung erhalten **(10.)** - Diese sollte der Familie so bald wie möglich nach dem Tod des*r Patient*inn ausgehändigt werden **(10.)** - Versendung eines persönlichen Kondolenzschreibens **(10.)** - Der beste Zeitpunkt für ein Kondolenzschreiben ist derzeit nicht bekannt, es sollte jedoch persönlich gestaltet sein, keine Verpflichtungen enthalten, die nicht eingehalten werden können, und Informationen über weitere Unterstützung enthalten **(10.)** - Bei Bedarf eine Liste örtlicher Hilfsdienste zur Verfügung stellen, die Menschen, die aufgrund eines Trauerfalls plötzlich verletzlich sind und sich möglicherweise selbst isolieren, praktische Hilfe und Unterstützung bieten können **(10.)** - Aktuelle Informationen und Anleitungen für die Organisation einer Beerdigung oder einer anderen religiösen Zeremonie und für die Registrierung eines Todesfalls zur Verfügung stellen und Anregungen und Ressourcen für künftige Zeremonien geben **(10.)** - Armut bei der Beerdigung kann für viele Angehörige ein Problem sein, so dass es hilfreich sein kann, auf Organisationen hinzuweisen, die in dieser Frage beraten können **(10.)** |
| Van Oorschot B et al.; März 2020 | Deutschland | Empfehlungen der Deutschen Gesellschaft für Palliativmedizin |  | Ausgabe von Empfehlungen für die Arbeitsweise von Palliativdiensten in Zeiten der COVID-19-Pandemie | - Sofortige Umstrukturierung der Arbeitsabläufe: offene Kommunikation mit Primärbehandler*innen sowie mit Patient*innen und Angehörigen, dass die Arbeitsweise des Palliativdienstes pandemiebedingt angepasst werden muss **(2.)** - Bei täglichen Teambesprechungen Mindestabstand (1,5m) einhalten und Gruppe möglichst klein halten **(1.)** - Weitere teaminterne Abstimmung möglichst telefonisch abhalten **(1.)** - Möglichst telefonische Erfassung der Symptomlast und Bedürfnisse **(1.); (4.)** - Einrichtung von Telefonsprechstunden für Angehörige aufgrund von Besuchsbegrenzungen und/oder Isolations- bzw. Quarantänemaßnahmen der Angehörigen **(4.)** - Im Einverständnis mit Patient*innen auch proaktive Telefonate mit Angehörigen zur Informationsweitergabe anbieten **(4.)** - Teambesprechungen auf ein Mindestmaß reduzieren, evtl. Skype-/Video-oder Telefonkonferenzen durchführen **(4.)** - Weitere Face-to-Face-Meetings wie z.B. Supervisionen und Fortbildungen soweit wie möglich aussetzen **(1.)** - Basishygienemaßnahmen für Mitarbeiter*innen und Patient*innen berücksichtigen **(1.)** - Mund-Nasen-Schutz nutzen **(1.)** - Möglichkeiten zur Unterstützung von Mitarbeiter*innen in besonderen Belastungssituationen bei Netzwerkpartner*innen (z.B. Psychoonkologie, Seelsorge, Sozialdienst) erfragen und organisieren **(6.)** - Ambulante Unterstützungsangebote für Angehörige einbeziehen (z.B. Pfarrer*innen in Kirchgemeinden) **(6.)** |
| Wallace CL et al.; Juli 2020 | USA | Diskussionspapier |  | - Beschreibung des Zusammenhangs zwischen der COVID-19-Pandemie und Trauer für Einzelpersonen, Familien und ihre Betreuer*innen - Diskussion über die Bewältigung der Trauer durch Kommunikation, Vorsorgeplanung und Selbstfürsorgepraktiken | - Vorbereitung von Patient*innen/Familien auf einen wahrscheinlichen Todesfall **(8.)** - Schwierige Gespräche sollten direkt angesprochen werden und es sollte sich nicht davor gescheut werden, Emotionen, Trauer und die allgemeine Not des*r Patient*in und der Familie in Gesprächen zur Pflegeplanung anzusprechen **(8.)** - Im Rahmen der Vorsorgeplanung sollte auch über gewünschte Rituale oder spirituelle Praktiken und Bestattungs-/Gedenkpläne gesprochen werden **(8.)** - Die Patient*innen/Familien sollten auf Ressourcen verwiesen werden, die ihnen helfen, die Bedürfnisse nach dem Tod zu berücksichtigen und es sollte zusätzliche Trauerunterstützung durch telemedizinische Dienste angeboten werden bzw. an diese vermittelt werden **(10.)** - Zu den Selbstfürsorgestrategien, die Einzelpersonen bei der Stressbewältigung helfen, gehören: **(6.)** - die Möglichkeit, Pausen einzulegen und sich vom Katastrophenereignis abzulenken **(6.)** - das Gefühl, vorbereitet und informiert zu sein, wenn es darum geht, ihre Rolle als Helfer*innen zu übernehmen **(6.)** - das Wissen um lokale Ressourcen und Dienste, an die sie Patient*innen für zusätzliche Unterstützung bei der Genesung verweisen können **(6.)** - eine angemessene Aufsicht und Unterstützung durch Gleichaltrige, während sie die Hilfe leisten **(6.)** |

***Tabelle 4: Anzahl der Publikationen zu den einzelnen Kategorien***

| **Kategorien** | **Publikationen** | **Gesamtzahl der Publikationen** |
| --- | --- | --- |
| 1. **Maßnahmen zur Infektionskontrolle** | Apoeso et al. 2021; Bausewein und Simon 2021; Bolt et al. 2021; Boufkhed et al. 2020; Boufkhed et al. 2021; Cohen-Mansfield 2020; Delis et al. 2020; Etkind et al. 2020; Griffin et al. 2020; Halek und Holle 2020; Hower et al. 2020; Kluge et al. 2021; Lazzarin et al. 2020; Luckett et al. 2021; Maaskant 2020; Mitchell et al. 2020; Powell und Silveira 2020; Roser et al. 2020; Schwartz et al. 2021; Van Oorschot et al. 2020 | *N=20* |
| 1. **Strukturelle Maßnahmen** | Abdihamad et al. 2020; Adams 2020; Apoeso et al. 2021; Bausewein und Simon 2021; Blinderman et al. 2021; Bloomer et al. 2021; Bolt et al. 2021; Boufkhed et al. 2020; Boufkhed et al. 2021; Cherniwchan 2021; Chomton et al. 2021; Cohen-Mansfield 2020; Dhahri et al. 2021; Dunleavy et al. 2021; Etkind et al. 2020; Griffin et al. 2020; Haire et al. 2021; Halek und Holle 2020; Hart et al. 2020; Hower et al. 2020; Kates et al. 2020; Kluge et al. 2021; Lazzarin et al. 2020; Luckett et al. 2021; Mitchell et al. 2020; Powell und Silveira 2020; Reinhard et al. 2021; Selman et al. 2020; Van Oorschot et al. 2020 | *N=29* |
| 1. **Besuchsregelungen** | Bausewein und Simon 2021; Berthold et al. 2020; Blinderman et al. 2021; Bloomer et al. 2021; Cohen-Mansfield 2020; Costantini et al. 2020; Delis et al. 2020; Dhahri et al. 2021; Etkind et al. 2020; Griffin et al. 2020; Halek und Holle 2020; Hsu et al. 2020; Janssen et al. 2020; Kates et al. 2020; Kluge et al. 2021; Luckett et al. 2021; Maaskant 2020; Münch et al. 2020; Roser et al. 2020; Schwartz et al. 2021; Selman et al. 2020 | *N=21* |
| 1. **Kommunikationsstrukturen** | Adams 2020; Apoeso et al. 2021; Bains et al. 2020; Bausewein und Simon 2021; Beneria et al. 2021; Berthold et al. 2020; Blinderman et al. 2021; Bolt et al. 2021; Boufkhed et al. 2020; Boufkhed et al. 2021; Cherniwchan 2021; Chomton et al. 2021; Cohen-Mansfield 2020; Davies und Hayes 2020; Delis et al. 2020; Dhahri et al. 2021; Dunleavy et al. 2021; Etkind et al. 2020; Florencio et al. 2020; Flores et al. 2020; Gergerich et al. 2020; Griffin et al. 2020; Haire et al. 2021; Halek und Holle 2020; Hart et al. 2020; Hower et al. 2020; Humphreys et al. 2020; Janssen et al. 2020; Kates et al. 2020; Kluge et al. 2021; Lazzarin et al. 2020; Luckett et al. 2021; Maaskant 2020; Morris et al. 2020; Münch et al. 2020; Ong et al. 2020; Pearce et al. 2021; Pickell et al. 2020; Powell und Silveira 2020; Reinhard et al. 2021; Roser et al. 2020; Schwartz et al. 2021; Selman et al. 2020; Van Oorschot et al. 2020 | *N=44* |
| 1. **Schulung und Aufklärung** | Abdihamid et al. 2020; Apoeso et al. 2021; Bains et al. 2020; Bausewein und Simon 2021; Blinderman et al. 2021; Boufkhed et al. 2020; Boufkhed et al. 2021; Cherniwchan 2021; Chomton et al. 2021; Cohen-Mansfield 2020; Dhahri et al. 2021; Etkind et al. 2020; Griffin et al. 2020; Haire et al. 2021; Halek und Holle 2020; Hart et al. 2020; Hower et al. 2020; Humphreys et al. 2020; Janssen et al. 2020; Kluge et al. 2021; Lazzarin et al. 2020; Luckett et al. 2021; Maaskant 2020; Münch et al. 2020; Pearce et al. 2021; Powell und Silveira 2020; Reinhard et al. 2021; Roser et al. 2020; Schwartz et al. 2021 | *N=29* |
| 1. **Psychosoziale Unterstützungsmaßnahmen** | Adams 2020; Apoeso et al. 2021; Bausewein und Simon 2021; Beneria et al. 2021; Berthold et al. 2020; Blinderman et al. 2021; Bloomer et al. 2021; Bolt et al. 2021; Boufkhed et al. 2020; Cherniwchan 2021; Chomton et al. 2021; Cohen-Mansfield 2020; Davies und Hayes 2020; Dunleavy et al. 2021; Etkind et al. 2020; Florencio et al. 2020; Flores et al. 2020; Gergerich et al. 2020; Griffin et al. 2020; Haire et al. 2021; Halek und Holle 2020; Hart et al. 2020; Hofmeyer und Taylor 2021; Hower et al. 2020; Janssen et al. 2020; Kates et al. 2020; Lazzarin et al. 2020; Maaskant 2020; Mitchell et al. 2020; Morris et al. 2020; Münch et al. 2020; Ong et al. 2020; Pearce et al. 2021; Pickell et al. 2020; Reinhard et al. 2021; Roser et al. 2020; Schwartz et al. 2021; Selman et al. 2020; Van Oorschot et al. 2020; Wallace et al. 2020 | *N=40* |
| 1. **Spezifische Überlegungen für Menschen mit Demenz** | Bolt et al. 2021; Cohen-Mansfield 2020 | *N=2* |
| 1. **Vorausschauende Pflegeplanung** | Bausewein und Simon 2021; Blinderman et al. 2021; Bolt et al. 2021; Davies und Hayes 2020; Delis et al. 2020; Florencio et al. 2020; Griffin et al. 2020; Halek und Holle 2020; Janssen et al. 2020; Münch et al. 2020; Ong et al. 2020; Schwartz et al. 2021; Selman et al. 2020; Wallace et al. 2020 | *N=14* |
| 1. **Abschiednahme** | Bausewein und Simon 2021; Beneria et al. 2021; Berthold et al. 2020; Bolt et al.2021; Costantini et al. 2020; Morris et al. 2020; Münch et al. 2020; Roser et al. 2020; Schwartz et al. 2021 | *N=9* |
| 1. **Maßnahmen nach dem Tod** | Bausewein und Simon 2021; Beneria et al. 2021; Berthold et al. 2020; Blinderman et al. 2021; Bolt et al. 2021; Janssen et al. 2020; Morris et al. 2020; Münch et al. 2020; Pearce et al. 2021; Roser et al. 2020; Selman et al. 2020; Wallace et al. 2020 | *N=12* |

1. https://www.dgpalliativmedizin.de/images/stories/Was_ist_Palliativmedizin_Definitionen_Radbruch_Nauck_Sabatowski.pdf [↑](#footnote-ref-1)
